# We need to talk about purpose: a critical interpretive synthesis of health and social care professionals’ approaches to self‐management support for people with long‐term conditions

**DOI:** 10.1111/hex.12453

**Published:** 2016-04-14

**Authors:** Heather May Morgan, Vikki A. Entwistle, Alan Cribb, Simon Christmas, John Owens, Zoë C. Skea, Ian S. Watt

**Affiliations:** ^1^ Health Services Research UnitUniversity of AberdeenAberdeenUK; ^2^ Centre for Public Policy ResearchKing's College LondonLondonUK; ^3^ Department of Health Sciences/Hull York Medical SchoolFaculty of ScienceUniversity of YorkHeslington, YorkUK

**Keywords:** chronic conditions, diabetes, long‐term conditions, patient empowerment, patient participation, professional–patient relations, support for self‐management

## Abstract

**Background:**

Health policies internationally advocate ‘support for self‐management’, but it is not clear how the promise of the concept can be fulfilled.

**Objective:**

To synthesize research into professional practitioners’ perspectives, practices and experiences to help inform a reconceptualization of support for self‐management.

**Design:**

Critical interpretive synthesis using systematic searches of literature published 2000–2014.

**Findings:**

We summarized key insights from 164 relevant papers in an annotated bibliography. The literature illustrates striking variations in approaches to support for self‐management and interpretations of associated concepts. We focused particularly on the somewhat neglected question of the *purpose* of support. We suggest that this can illuminate and explain important differences between narrower and broader approaches. Narrower approaches support people to *manage their condition(s) well* in terms of disease control. This purpose can underpin more hierarchical practitioner–patient communication and more limited views of patient empowerment. It is often associated with experiences of failure and frustration. Broader approaches support people to *manage well with their condition(s)*. They can keep work on disease control in perspective as attention focuses on what matters to people and how they can be supported to shape their own lives. Broader approaches are currently less evident in practice.

**Discussion and conclusion:**

Broader approaches seem necessary to fulfil the promise of support for self‐management, especially for patient empowerment. A commitment to enable people to live well with long‐term conditions could provide a coherent basis for the forms and outcomes of support that policies aspire to. The implications of such a commitment need further attention.

## Background

The idea that health professionals can usefully advise people how to look after their health and manage their health conditions is not new, but a concept of ‘support for self‐management’ among people with long‐term conditions has been given renewed emphasis in formal health service provision within many countries in recent decades.

Enthusiasm for support for self‐management has developed around several ostensibly promising ideas, for example that people with long‐term conditions have to manage somehow as they go about their daily lives, and support helps them to manage better; technological developments mean people can take on condition‐monitoring and treatment tasks that were previously the domain of health professionals; support for self‐management recognizes, develops and harnesses people's assets (empowering and working with them as partners rather than emphasizing their deficits and reinforcing dependency); support for self‐management reduces people's needs for health services and thus renders those services more sustainable; and support for self‐management offers people more control over their lives, empowering them and enhancing their well‐being as well as their health.^1^, ^2^, ^3^, ^4^

It is not clear how all these promising ideas hang together, or whether and how they can be co‐achieved. In practice, while initiatives to promote (or provide) support for self‐management by health and social care practitioners in routine care have generated some positive effects, they have not fulfilled all the policy aspirations.^5^, ^6^ There are several possible reasons for this. For example, the appropriateness of some interventions has been questioned because they do not seem to pursue what people with long‐term conditions themselves strive for, or use the kinds of strategies they have found supportive.^7^, ^8^, ^9^ Poor theorization of support for self‐management may also be a factor. Although there have been developments with attention to social learning theory and concepts of self‐efficacy and patient activation,^10^, ^11^ a strong psychological focus has perhaps led to the neglect of socio‐economic considerations.^12^ Some influential descriptions of support for self‐management are prone to problematically reductionist interpretations and arguably fail to reflect what skilful practitioners do to generate valued experiences of support.^13^

As part of a larger project to develop a conceptualization of support for self‐management that can recognize and encourage ‘good’ forms and experiences of support, we sought to understand how support for self‐management and associated ideas has been interpreted by health and social care practitioners and in research intended to support practice improvement.

## Methods

### Study design

We undertook a configurative review of literature, using a critical interpretive synthesis approach because this is oriented to conceptual or theoretical development and allows for critical consideration of diverse studies and the research traditions and assumptions that have influenced them.^14^

We had been sensitized to the idea that high aspirations for support could be understood in terms of ‘enabling people to live well’,^13^ and our guiding question was as follows:

What can we learn from existing research about health or social care practitioners’ perspectives, practices and experiences of supporting adults with long‐term conditions to manage and/or live well with those conditions?

### Sources and selection

An information specialist designed and executed searches for papers indexed on MEDLINE, CiNAHL, SCI and ASSIA databases. The search strategies combined terms relating to long‐term conditions with terms relating to self‐management, patient involvement or professional–patient relationships and practitioners’ perspectives. They prioritized sensitivity over specificity. The search was run initially for papers published between 2000 and 2013. Four authors worked in rotating pairs to screen first titles and abstracts then the full texts of potentially relevant papers. All authors met to discuss the relevance of particular topics and papers and to consider possible interpretations.

When selecting papers, we were primarily concerned with conceptualizing the support health and social care practitioners give in the context of routine care provision (as contrasted with ‘additional’ external education or support programmes for patients – although we were interested in how practitioners working in routine care viewed and complemented these programmes). We took an inclusive view of long‐term conditions, considering any health‐related problem that could last over 6 months. We excluded studies focusing on support for people with severe mental illness or advanced dementia, or in the context of care at the end of life, judging these to warrant separate and specific consideration. We prioritized papers offering useful conceptual insights.

### Data extraction, development of annotated bibliography and synthesis

We developed a structured form to summarize bibliographic information, study design, key findings and original authors’ discussion points and to note our own critical and interpretive comments on relevant papers. Forms were prepared by one author then checked (for accuracy) and added to by another. The completed forms and full texts of papers judged to offer particularly useful insights were shared among all authors to support critical discussions and the development of our interpretive synthesis.

To facilitate consideration of the literature as a whole, we produced an annotated bibliography that summarized relevant insights from the papers we found most useful in developing our synthesis and from a selection of others that illustrated a range of interpretations of support for self‐management.

### Search update

We updated our search of bibliographic databases to cover papers published in 2014. Two authors screened titles and abstracts then full‐text papers, assessing relevance in the light of the initial synthesis.

### Findings

Our primary bibliographic search (to 2013) identified 4566 titles/abstracts (deduplicated). A total of 3517 of these were judged not relevant to our initial guiding question, 707 were put aside as having only tangential relevance, and the full‐text papers for 342 were obtained and further assessed. Fourteen additional relevant papers were identified from the authors’ collections and by following up links between ideas and references as the review progressed. Some data were summarized from 227 of the 356 papers considered. A total of 153 papers were included in the annotated bibliography with summaries of the points we judged key for this review.

The update search identified 680 titles/abstracts, and entries for 11 papers were added to the annotated bibliography, bringing the total to 164. The new papers tended to confirm or extend, rather than challenge, the initial synthesis.

Studies with a diverse range of aims and methods have generated *some* data relating to practitioners’ practices, experiences and views of supporting people with long‐term conditions. They considered a range of practitioners (health and social care professionals from different disciplines) and their work with people with diverse long‐term conditions (although diabetes was the focus of 61 of 164 studies in the annotated bibliography) in various countries (although mainly UK and north‐west Europe, North America and Australia/New Zealand) and service settings (with different disciplinary mixes, across primary and secondary care, and in urban and rural areas).

The literature reflects a significant interest in improving the support health services offer people with long‐term conditions, but the question of what constitutes good support has received relatively little critical attention. Many papers noted practitioners’ concerns about what could be considered well‐documented ‘operational obstacles’ to the provision of adequate support, particularly short consultation times, staff shortages and organizational structures or systems that impede coordination of care.^15^ A large proportion of papers reported the development and/or evaluation of interventions intended to enhance the support provided within and/or beyond conventional health‐care consultations.

Taken as a whole, the literature indicates significant diversity in the ways that practitioners go about supporting people with long‐term conditions. Several papers that were particularly reflective about this diversity and some of its implications provided a useful stimulus to our analysis. These included reports from qualitative research that identified important variations among practitioners in terms of (i) how they worked with people with long‐term conditions, why they did what they did, and what significance they attached to their actions and experiences^16^, ^17^, ^18^ and/or (ii) how they interpreted ideas about ‘individualizing’ care and ‘involving’ or ‘empowering’ patients.^19^, ^20^ By considering particularly what was said or implied about the purpose of support and the scope for people with long‐term conditions to be influential, we developed a characterization and critique of narrower and broader approaches to support for self‐management.

### Outline of synthesis

In outline, our critical interpretive synthesis is as follows:

A variety of practices and ideas are associated with the concept of support for self‐management. Practitioners’ approaches to support can be considered to be narrower or broader in several respects, including the purpose to which the support is oriented, the views taken of people with long‐term conditions, the forms of support offered and considered appropriate, the typical features and perceived value of professional–patient relationships, and the criteria used to judge the success of support (see Table 1).

**Table 1:** Classification of practitioners’ approaches to support

	Narrower approaches and interpretations	Broader approaches and interpretations
Purpose of support	Help patient to manage their conditions well Biomedical ‘end’ goals: control symptoms; reduce risk of disease progression and complications Behavioural ‘means’ goals: lifestyle, self‐monitoring and medication taking oriented to biomedical goals	Help person to manage well (live well) with their conditions Quality of life goals including maintaining social roles and valued identities, finding meaning in life, developing and exercising autonomy as well as biomedical health
Indicators of success	Disease‐control and biomedical markers of this Compliance with behavioural regimes oriented to disease control	Reports or measures of various aspects of living well (criteria for this can be person‐specific and dynamic)
Forms of support	Tend to be transactional, controlling Two main strategies: didactic education and persuasive motivation	Tend to be more relational, responsive More diverse and flexible strategies Practitioner–patient relationship can be constituent of support
Practitioner–patient relationship	Hierarchy of expertise and authority regarding condition‐ management	Collaborative alliance, more mutually respectful partnership
View of patient empowerment	Patient enabled to carry out condition‐management tasks Patient granted permission to take responsibility for condition‐control tasks	Person enabled to influence agenda setting and decision‐making in discussion with practitioners, to express critical opinions, to find and act on ways to manage and live well with conditions
Scope of professional interest	Patient's conditions and what can be done to manage them Patient's understanding and motivation (and perhaps other features of their situation) as these can affect condition control What can be done within a consultation (or by referral) to promote compliance	Person, their life, what matters to them and how their conditions impact on these (as well as *vice versa*) Person's abilities to solve problems relating to their conditions, to exercise autonomy etc. What can be done within a consultation (or referral), and how that relates to other forms of support that contribute to the person's scope to live well
We identified two main purposes to which support tends to be oriented, although we note that these are not always explicitly articulated or discussed. First, support can be somewhat narrowly oriented to helping people to *manage their conditions well* in biomedical or disease‐control terms. Second, support can be more broadly (and usually more flexibly) oriented to helping people to *manage well (or live well) with their conditions*.
We suggest that the view of purpose that practitioners (perhaps implicitly) adopt can help explain other features of their approaches to support for self‐management. Efforts to support people to work on the management *of* their conditions tend to be associated with narrower versions of the other dimensions and particularly with less radical scope for patient empowerment. Efforts to support people to live well *with* their conditions tend to be associated with broader versions of the other dimensions, including more radical scope for patient empowerment.
Of course, these are not neat dichotomies, and attention to the management of conditions can be incorporated within efforts to support people to live well with conditions. In general, however, the distinction between narrower and broader seems to hold across multiple elements.
Our reflections lead us to suggest that an important explanation for the clustering of narrower features lies in the way that an orientation to disease control can limit what is seen of a person with long‐term conditions, of what matters in people's lives, and of how people can be supported to shape their own lives. An orientation to living well, in contrast, invites careful attention to these and thus to broader and more responsive approaches to support for self‐management.

We now present a fuller account, with illustrative references, of what we take to be the main features of narrower and broader approaches to support for self‐management. We include our reflections on the significance of ideas about the purpose of support (which are not always explicit in particular papers) for understanding these.

### Narrower approaches: supporting people to manage their conditions

The (often implicit) starting point for thinking about support for self‐management is the recognition that clinical and epidemiological research indicates there are, at least in principle, actions that people can take to moderate the course of their long‐term conditions and that health professionals have expertise that can inform and otherwise help them with these. This recognition is particularly evident in the context of conditions such as diabetes. From a biomedical perspective, an initial idea emerges that the purpose of support for self‐management is to help people to contribute to the effective management *of* their health conditions – to improve control of their symptoms and to reduce the risks of disease progression, exacerbations or complications.

The disease‐control view of purpose is evident in the use of biomedical indicators (and/or assessments of people's adoption of behaviours that can improve these) to judge the success of support for self‐management and interventions to promote it. (In the literature, and perhaps in practice, the purpose of support is perhaps more readily inferred *from* these indicators and assessments: beyond the general assumption that support for self‐management should improve health, the question of its overall purpose is not often discussed).

The merit of this starting point is reflected in enthusiasm for strengthening support for self‐management in situations where people have not previously been well informed or encouraged to engage in effective strategies for disease control. Approaches to support for self‐management associated with a strong emphasis on disease control are, however, open to a number of critiques.

On narrower interpretations, people with long‐term conditions are viewed as (potential) contributors to the biomedical management of those conditions. Support is typically geared to encourage them to adopt the condition‐monitoring, treatment and/or lifestyle behaviour regimes that in general terms contribute to disease control (and that may previously have been undertaken by practitioners). The term ‘self‐management’ can sometimes seem to be equated to compliance with professional recommendations.^21^

The forms of support typically associated with an orientation to the effective management of conditions reflect concerns to ensure that people know what is recommended for disease control, have the skills and confidence needed to use any equipment and medicines prescribed to monitor and treat their conditions and are motivated to comply. The repertoire of forms of support that practitioners offer seems limited to didactic education and motivation, and practitioner–patient communication tends to be considered in task‐oriented, transactional and somewhat instrumental terms.^22^, ^23^, ^24^ Attention to emotional issues might feature,^25^ but practitioners’ engagement with patients’ lived experiences appears limited, perhaps because attention to emotional issues is valued primarily as a means to encourage behaviours recommended for disease control.

The idea that practitioners have a role to play in encouraging patients to monitor and respond to biomedical indicators of their conditions features strongly within narrower approaches. This idea is sometimes overlaid with a view that practitioners with biomedical expertise are needed to authorize or permit such activities. Particularly,when they control the development of care plans and access to condition‐management technologies (e.g. anticoagulant titres, insulin pumps), some practitioners decide whether and what kinds of condition‐monitoring and medication adjustment particular patients should (or might be allowed to) do for themselves, and limit access to those they deem sufficiently knowledgeable and committed.^26^, ^27^, ^28^, ^29^, ^30^ Some appear disinclined to trust or engage with data that patients generate in self‐monitoring^31^ and some discourage any self‐adjustment of medications.^32^

When the focus is on disease control, practitioners sometimes seem to assume positions of superior authority from which they monitor and judge patients and their progress.^23^, ^33^ They perhaps interpret biomedical indicators as ‘signalling the truth’ and allow these to dominate conversations.^34^ An orientation to disease control seems to allow (although does not require) professional–patient relationships to be viewed and enacted as hierarchies of expertise and authority.

Within narrower approaches, practitioners might talk of ‘individualizing’ support, ‘empowering’ patients and ‘involving’ them in decision‐making or goal setting, but in practice, they tend to restrict the scope for patients to participate and influence decisions.^18^, ^20^, ^35^, ^36^ They might encourage patients to take responsibility for particular condition‐management tasks, but only in accordance with professional direction.^37^, ^38^ The language of empowerment is thus sometimes used in a very weak sense and made consistent with the preservation of a strong practitioner–patient hierarchy.^17^ In these circumstances, patients are only considered partners in the sense that they work co‐operatively with the professionals in authority.

#### Perceptions of and responses to challenges to patients’ self‐management

Optimism about what support for self‐management *could* achieve seems fairly widespread in the literature, including in the outcome measures used in evaluative study designs. Many examples show, however, that didactic education and persuasion‐oriented motivation do not reliably ensure patients adopt recommended self‐management behaviours or achieve biomedical disease‐control targets.^23^, ^39^, ^40^

Narrower approaches can seem to prompt quite negative judgements of patients. Some practitioners apparently assume that ‘non‐compliant’ patients either have not understood their advice or have wilfully chosen not to take responsibility for their health.^22^, ^41^ Some recognize that patients’ health condition(s), perhaps particularly depression, can impair their potential to self‐manage,^42^, ^43^ but practitioners can also tend to view non‐compliant patients as difficult, dishonest, and even as barriers to the provision of good care.^23^, ^39^, ^40^, ^44^, ^45^, ^46^, ^47^

When narrow approaches to support focus on people's knowledge, skills and motivation, they can tend to neglect the psycho‐social and socio‐economic circumstances that make it hard for some people to prioritize and act on professional advice about condition management.^13^

They also seem to offer practitioners few options for dealing with non‐compliant patients beyond: persisting with didactic strategies, hoping that eventually these will win patients round to their point of view; accepting a need to settle, at least temporarily, for less or slower progress than they would consider ideal (e.g. setting smaller, intermediate goals); referring patients to other sources of education or support (although perhaps only those known to work to the same biomedical goals); and/or regarding the non‐compliance as a matter of patient choice that they can do little or nothing about.^28^, ^48^

Adopting a negative view of patients can help practitioners maintain a sense of superiority and can be understood as a strategy for reinforcing a sense of professional identity when that identity is primarily associated with a hierarchy of expertise and authority.^23^ But practitioners do sometimes experience a sense of failure on their own part and can be prone to disillusionment when their practice is unsuccessful in terms of disease control or of changing patients’ behaviours for the sake of this.^49^ Several studies report feelings of frustration among practitioners,^22^, ^50^ and sometimes, both patients and practitioners end up blaming themselves for their failure.^40^, ^49^

Studies that examine patients’ perspectives alongside practitioners’ indicate that practitioners can tend to underestimate: patients’ understanding and motivation relating to condition management, the practical difficulties patients can face when trying to implement and achieve what they have been asked to, people's experiences of condition‐related distress and treatment side‐effects, and what patients might achieve if given more personally responsive emotional or practical support.^32^, ^51^, ^52^, ^53^, ^54^ They also suggest that practitioners often have very limited awareness of patients’ views about how their conditions recommended management regimes and professional support affect them, and about what it would mean to them to live well (or even to have a normal life) with their conditions.^55^, ^56^, ^57^ There is an additional sense of shortfall in the extent to which practitioners working with narrower approaches to support for self‐management take seriously patients’ experiences of life and opinions,^58^, ^59^ foster meaningful forms of participation^38^ or self‐reliance,^60^ and/or recognize and support people as agents or actors of their own lives. Some studies observe a tight boundary around what is considered relevant for patients and practitioners to discuss in consultations.^35^, ^57^, ^61^

Overall, a focus on disease control seems likely to foster a rather contained view of scope of the interest practitioners need to take in patients***.*** On narrower approaches, there is often little sense that practitioners could or should usefully engage in any serious way with patients’ lives beyond the clinic, or indeed to liaise with other potentially supportive services.

### Broader approaches: supporting people to live well with conditions

Broader approaches to support for self‐management are more evident in comments about their desirability than in accounts of practice. Our characterization draws on both, as well as comparisons between the perspectives of practitioners and of people with long‐term conditions from papers that reported both. Broader approaches can diverge from narrower ones to greater or lesser extents.

In terms of purpose, practitioners working with broader interpretations tend to (i) be oriented towards supporting people to achieve a better quality of life (or a richer view of health than disease control) and/or (ii) put more emphasis on supporting the development of patients’ autonomy, self‐determination or similar.^62^, ^63^, ^64^, ^65^ Some focus on developing patients’ self‐efficacy and promoting patient‐led goal setting, but we note these can either be tied back to, or liberated from a disease‐control orientation (see ‘Discussion’). On the broadest interpretations, ideas about quality of life cover both the present and the future, incorporate people's own views of what is important in their particular lives, and take seriously the idea that it matters that people can shape their own lives rather than have them shaped by others. The concept of ‘living well’ seems to accommodate all the broadest senses of purpose.

When working with a broader idea of purpose, the question of what constitutes success is somewhat open‐ended. Biomedical indicators can still have significance (and do so particularly in some contexts), but may be poor proxies when practitioners’ support is more broadly oriented to improving quality of life and more flexibly responsive to people's variable views of what matters for living well. Practitioners working with broader interpretations of support seem inclined to recognize progress in a number of domains, including how well people adapt to and cope with having long‐term conditions,^66^ what sense of control they have,^56^, ^67^, ^68^, ^69^, ^70^ and to what extent they can think critically and respond or develop their own solutions to health‐related problems.^37^, ^71^

Diverse forms of support can seem relevant within broader interpretations. Crucially, however, in order to be responsive to individuals, practitioners must work flexibly.^64^, ^72^, ^73^, ^74^, ^75^

More broadly supportive practices seem to incorporate careful attention to the person, their life circumstances and lived experiences, as well as to the condition.^74^, ^76^, ^77^, ^78^ They also create scope for the person to shape the agenda for discussion and action with their practitioners. They can involve practitioners: taking time to listen and get to know the person and what's important to them;^37^, ^79^ being sensitive to the context of the person's life and priorities;^18^ negotiating the form of consultations with patients;^80^ attending to the person's own situational assessment and co‐constructing understandings of the concerns/problems to be worked on;^81^ being receptive to and working with patients’ expressions of emotion;^82^, ^83^ working collaboratively to form plans and set goals, being led by what matters to the person;^84^ providing responsive and appropriate education about the condition;^85^, ^86^ setting up an expectation that the person will learn to manage and live well with the condition, and that they will be supported in doing so;^66^ positively encouraging the person to express their opinions, and engaging with them honestly when they differ from the practitioner's;^29^ and actively trying to help the person develop their knowledge, skills, confidence and autonomy so they can better take responsibility for their lives and develop their own solutions to emergent challenges.^37^, ^64^, ^71^, ^87^, ^88^

Practitioners oriented to support people to manage well *with* their conditions need not neglect the importance of supporting people with the management *of* their conditions. They continue to be interested in how people can contribute to their own health and try to encourage and reinforce health‐ or recovery‐ promoting behaviour.^66^ Rather than stick narrowly to the focus on condition management, however, those with a broader orientation apparently move *between* considerations associated with the pursuit of biomedical outcomes and considerations relating to quality of life and autonomy.^76^

Within broader approaches, more emphasis is likely to be put on the attitudes that underpin the tone and quality of practitioners’ communication with patients. The professional–patient relationship can become particularly significant, not just as an instrumental means to encourage people to act but also as somehow consti‐tutive of the support that practitioners offer.^50^, ^64^, ^79^, ^89^, ^90^, ^91^ Practitioners working with broader approaches might seek quite explicitly and strenuously to relate to patients as individuals^82^ and to build trust and develop rapport with those they work with.^16^, ^92^ Broader interpretations of support can recognize value in practitioners being present for a person and acknowledging and sharing their burden.^88^, ^93^

Broader approaches are in part characterized by less hierarchical, more equitable and mutually respectful professional–patient relations. We suggest these are more readily fostered when support is understood to have a more open‐ended purpose that includes a concern to support people's autonomy. This is more compatible with practitioners seeking to work with people in collaborative alliances or partnerships^17^, ^63^, ^64^, ^94^, ^95^, ^96^ and being willing to learn from patients as well as to teach them.^72^ It is also more compatible with interpretations of patient involvement and patient empowerment that have a deeper meaning and more radical implications than those associated with a focus on disease control. Broader interpretations of support for self‐management thus have richer implications for bolstering people's capability for self‐determination.^62^

#### Perceptions of and responses to challenges to patients’ self‐management

Within broader approaches, more attention is paid to people's own perspectives and their interests in directing their own lives. This should mean that, while practitioners can still be concerned about condition management, they are less likely to judge people who do not share their viewpoints as ‘difficult patients’.^96^, ^97^ We suggest that practitioners who adopt the kinds of responsive supportive practices outlined above (listening to people and taking their life circumstances, lived experiences, personal concerns and priorities seriously, etc.) are less likely to misunderstand people, to ‘miss’ issues relevant to condition management, to impose standardized goals and strategies inappropriately, or to act in ways that seem manipulative. They are more likely to recognize the challenges in people's lives that can make condition‐management tasks both more difficult and less of a priority,^96^ and less likely to treat condition‐management tasks as the only things that matter.^37^ On broader approaches, communication may be designed to express empathy rather than judgment^86^ and to avoid generating feelings of guilt in patients.^51^, ^98^, ^99^

#### Issues in implementing broader approaches to support for self‐management

The literature suggests that practitioners who aspire more broadly to help people live well with their conditions sometimes struggle to do so in practice. There can be substantial gaps between what practitioners want to do (or say they do, when talking in the abstract) and what they are observed to do or report having done in particular cases.^20^, ^100^, ^101^ Some practitioners (including those who have been intensively trained and supported to orient their practice to broader purposes) have found the process of transitioning complex and uncomfortable – although some also experience the shift as ultimately positive.^33^, ^72^

There are several reasons why it can be hard to adopt broader approaches. Supporting people to solve their own problems runs counter to some conventional professional training and practices.^62^, ^102^ Being responsive to people in a holistic rather than a condition‐determined sense requires nuanced and flexible interpersonal skills and may require tricky judgement calls, for example if people have reasons not to be completely honest and open with practitioners;^98^, ^103^ it is unclear how responsibility should be allocated;^72^ practitioners feel torn between their sense of professional duty to reduce the risk of harm (including disease) and their concern to recognize people as the rightful controllers of key aspects of their own lives;^104^, ^105^, ^106^ or people seem to resist efforts to empower them.^19^, ^107^, ^108^. In addition, efforts to empower people sometimes fail to achieve the intended effect.^36^, ^109^, ^110^ Not surprisingly, some practitioners lack the skills or confidence to practice in the more flexible and responsive ways associated with broader approaches to support.^71^, ^111^, ^112^

For practitioners working in routine health service settings, transitioning from an orientation to support condition management via education and persuasion to an orientation to support people to live well with their conditions can require multiple far‐reaching modifications to practice, and perhaps a passage through a sense of role conflict and questioning of the appropriate scope of service provision.^41^, ^113^ The adoption of broader approaches can be particularly difficult for practitioners whose colleagues continue to operate with narrower understandings^114^, ^115^ and who work within highly medicalized cultures and under policies that reflect and reinforce a focus on evidence and biomedical outcomes.^35^, ^104^, ^107^, ^108^, ^116^, ^117^ The tensions between competing paradigms are perhaps greater when patients are (on narrower interpretations) ‘non‐compliant’.^100^, ^105^

## Discussion

Our extensive literature search and critical consideration of a wide range of studies identified significant diversity among health and social care practitioners’ (and authors’) approaches to support for self‐management and interpretations of associated concepts. We have suggested that these approaches and interpretations can be characterized as narrower and broader in a number of different respects. We have drawn attention to the often implicit purpose of support and distinguished an orientation to help people manage their conditions well (associated with narrower approaches) from an orientation to help people manage well with their conditions (associated with broader approaches). We are not saying that the two views of purpose or the narrower and broader approaches that we have outlined represent completely clear‐cut distinctions in practice, but we do think the distinctions have practical value for discussions about the promotion and evaluation of support for self‐management, including in relation to the various ostensibly promising ideas around which enthusiasm for the concept has gathered. Our relatively simple groupings and distinctions offer a manageable way to recognize the coexistence of multiple interpretations of support for self‐management, to facilitate effective communication that can overcome the inconsistent use of key terms and to reflect critically on similar‐sounding but perhaps importantly different approaches.

Our synthesis draws on studies conducted among practitioners from a range of professional backgrounds who work with people with diverse long‐term conditions and in different settings. Although it is possible that our search strategy missed some relevant studies and that other researchers would have selected different studies for inclusion, our approach was broad ranging and our primary concern was for conceptual relevance, and we believe our synthesis has broad applicability. Numerous studies of the experiences of people with long‐term conditions tend to confirm the concerns we have highlighted with narrower approaches that stick strongly to disease‐control ideals. The high proportion of papers relating to diabetes does, however, warrant comment. The actions people with type 1 or type 2 diabetes can take (in terms of their diet, exercise, condition monitoring and medication management) often have particularly significant implications for both the shorter and longer term control of the disease (including avoidance or otherwise of hypo‐ or hyperglycemic emergencies in the shorter term, and of complications of diabetes such as blindness, neuropathy and vascular problems leading to amputations), and hence quality of life. This is reflected in widespread use of targets for biomedical control as indicators of health‐care quality in diabetes that perhaps make it particularly challenging for practitioners to move away in any thoroughgoing sense from narrower approaches to support for self‐management. In some other long‐term conditions, for example motor neurone disease, there is less people can do to control the disease and its progression. The strong focus on diabetes within the literature on support for self‐management might have tended to encourage the development and use of relatively narrow approaches.

The synthesis we developed appreciates the origins of narrower approaches within health‐care contexts and acknowledges the value of support for what people can do to improve or prevent the worsening of their conditions. It tends to confirm that narrower approaches often fall short of policy aspirations, but it also helps to explain why. A strong orientation towards disease control can foster a neglect of people's wider personal and social contexts. This can lead to important support needs being missed and the effectiveness of supportively intended interventions being reduced. A strong orientation to disease control can also lead to patients’ agency being valued only (or primarily) instrumentally, and to the adoption of very limited views of empowerment. This is not a strong basis for ensuring people can have more control over their lives or for enhancing their well‐being in any significant sense – especially when the burden of disease‐control regimes is high and practitioners’ orientations to disease control tend to undermine people's self‐evaluations and moral identities.

Broader approaches can include attention to the potential for disease control that is the focus of narrower views, but keep this in some kind of balance among other considerations. Some influential ideas within the general literature on self‐management support could perhaps have strong resonances with approaches we have considered ‘broader’. For example, patient‐led problem‐solving and goal setting, or patient ‘activation’ can seem consistent with efforts to empower and give people more control, and have been associated with demonstrable improvement in outcomes for some patients.^10^, ^11^, ^118^ However, to the extent that no further purpose is specified beyond patient‐led activity or patient activation, we suggest that attempts to adopt and assess these kinds of practice (or forms of support) will, especially in medicalized cultures, tend to be subsumed back within a frame of disease control and be prone to the limitations of narrower approaches. For example, accounts of the kinds of goals people might set often reflect progress towards (or partial adoption of) behaviours recommended for disease control rather than actions that are otherwise important to people striving to manage well with conditions (and it may be that some of the things that matter to people are not readily or appropriately articulated as explicit goals^13^).

The broader purpose of supporting people to manage well with their conditions is somewhat open‐ended. This, together with the diversity and dynamics of people and their situations, means it is probably impossible to list a set of actions that can reliably constitute effective and appropriate forms of support. We suggest that a robust sense of purpose is needed to provide an overarching and action‐guiding ‘why’ that can serve to stimulate and appraise ideas about the forms that support might take.

### Towards a reconceptualization of support for self‐management

We propose that ‘enabling people to live (and die) well with their long‐term condition(s)’ is a strong candidate statement of the purpose of support for self‐management. This ambitious and flexible expression of an orientation to support people to manage well can accommodate attention to disease control, but should help avoid the problems associated with a narrow and strong focus on that. It can also accommodate the potential value of practitioners’ efforts to develop people's self‐efficacy and sense of responsibility, but again should help avoid the limitations of unduly narrow and prescriptive interpretations of these concepts. If the purpose is to help people live well, setting disease‐control constraints on goal setting and ideas about responsibility seem less reasonable.

Living well with long‐term conditions can include coping and adjusting to those conditions, and more ambitious aspirations for human flourishing – at least to the extent that the long‐term conditions allow. And in a way that neither ‘health’ nor ‘quality of life’ can do quite so readily, the phrase ‘living well’ within our candidate purpose statement encourages recognition of each person as a uniquely positioned actor in their own life. ‘Living well’ must in some senses be done on one's own terms, and the concept can somehow integrate people's interests in their autonomy and/or shaping of their own lives with concerns about their overall well‐being or quality of life. It does not, however, reduce to ideas of preference and choice.

Our synthesis and proposed statement of purpose lead us to suggest that when concepts like empowerment and involvement are used to describe approaches to support for self‐management, questions need to be asked about the scope of what people are empowered to do (e.g. act to manage the condition, choose what behaviour they want to try, or enhance their scope to live well despite the condition) and about the scope created for people to develop, express and pursue their own values and priorities (beyond disease control).

It is important to recognize that people with long‐term conditions are likely to have varying hopes and expectations about the support they might get from health professionals and others to manage and more broadly live well with their long‐term conditions. Low socio‐economic status and experiences of unhelpful health professionals seem to foster low expectations of support.^119^, ^120^ But even if patients are not expecting their health professionals to engage positively with their broader concerns for living well, they generally value being treated with care and respect, including being informed and enabled.^121^ Unless health professionals work with a broader awareness of the ways they can impact on people's opportunities, they are in danger of undermining people's experiences of health care and potential to live well with long‐term conditions, for example with their expressions of negative judgement and distrust.

The available evidence suggests that practitioners face a number of challenges and tensions if they shift their orientation from a focus on supporting the self‐management of disease control to a focus on enabling people to live well with long‐term conditions. The challenges are likely to be particularly acute in situations where targets for biomedical control feature particularly strongly as indicators of care quality and as bases for financial reward to service units (as is often the case for diabetes). Some tensions will remain even with shifts in professional and organizational cultures because, for example, multiple things can matter for a person's living well and for different reasons, and these will not always be compatible. Both the theorization and the practical implications of ideas about living well with long‐term conditions as the purpose of support need further work, but these ideas do seem to have potential to help support for self‐management fulfil the promise associated with it.

## Conclusions

Ideas (including sometimes implicit assumptions) about the purpose of support for self‐management need careful attention in policy, practice and research contexts. When efforts to support self‐management are ultimately oriented to disease control, they are unlikely to be compatible with the broader aspirations of person‐centred practice. An intention to enable people to live (and die) well with their long‐term conditions is a more appropriately ambitious and flexible overarching purpose of support.

## Contributions

VAE initially conceived the study, and all authors discussed and refined the design. Cynthia Fraser led the design of and conducted the literature searches and facilitated document retrieval and reference management. HMM led document management. HMM, VAE, JO and ZCS screened titles and abstracts, assessed full‐text papers and prepared document summary forms. All authors contributed to discussions about the interpretation of key papers and the development of the synthesis. HMM and VAE led the drafting of the manuscript, with all authors contributing critically to revisions and agreeing the final version.

## Funding

This research was funded by The Health Foundation (Project reference 7209).

## Declaration of conflicts of interest

The authors declare no conflicts of interest.

## Supporting information


**Table S1**. Annotated bibliography.

## References

[1] Long‐term Conditions Alliance Scotland (LTCAS), The Scottish Government . Gaun Yersel: The Self Management Strategy for Long Term Conditions in Scotland. Glasgow: LTCAS, 2008.

[2] Adams K , Corrigan JM . Priority Areas for National Action: Transforming Health Care Quality. Washington, DC: National Academies Press, 2003. 25057643

[3] Riegel B , Moser DK , Anker SD *et al* State of the science: promoting self‐care in persons with heart failure: a scientific statement from the American Heart Association. Circulation, 2009; 120: 1141–1163. 19720935 10.1161/CIRCULATIONAHA.109.192628

[4] Department of Health . Supporting People with Long Term Conditions: An NHS and Social Care Model to Support Local Innovation and Integration. London: Department of Health, 2005.

[5] Coulter A , Entwistle VA , Eccles A *et al* Personalised care planning for adults with chronic or long‐term health conditions. Cochrane Database of Systematic Reviews, 2015; 3: CD010523. 10.1002/14651858.CD010523.pub2 PMC6486144 25733495

[6] Taylor S , Pinnock H , Epiphanou E *et al* A rapid synthesis of the evidence on interventions supporting self‐management for people with long‐term conditions: PRISMS – Practical systematic Review of Self‐Management Support for long‐term conditions. Health Services and Delivery Research, 2014; 2: 1–622. 25642548

[7] Bury M . Chronic illness as biographical disruption. Sociology of Health & Illness, 1982; 4: 167–182. 10260456 10.1111/1467-9566.ep11339939

[8] Carel H . Illness (the Art of Living). London: Routledge, 2008.

[9] Townsend A , Wyke S , Hunt K . Self‐managing and managing self: practical and moral dilemmas in accounts of living with chronic illness. Chronic Illness, 2006; 2: 185–194. 17007695 10.1177/17423953060020031301

[10] Hibbard JH , Stockard J , Mahoney ER *et al* Development of the Patient Activation Measure (PAM): conceptualizing and measuring activation in patients and consumers. Health Services Research, 2004; 39(4 Pt 1): 1005–1026. 15230939 10.1111/j.1475-6773.2004.00269.x PMC1361049

[11] Holman H , Lorig K . Patient self‐management: a key to effectiveness and efficiency in care of chronic disease. Public Health Reports, 2004; 119: 239–243. 15158102 10.1016/j.phr.2004.04.002 PMC1497631

[12] Sinding C , Miller P , Hudak P *et al* Of time and troubles: patient involvement and the production of health care disparities. Health: An Interdisciplinary Journal for the Social Study of Health, Illness & Medicine, 2012; 16: 400–417. 10.1177/1363459311416833 21856716

[13] Entwistle V , Cribb A . Enabling People to Live Well: Fresh Thinking About Collaborative Approaches to Care for People with Long Term Conditions. London: The Health Foundation, 2013.

[14] Dixon‐Woods M , Cavers D , Agarwal S *et al* Conducting a critical interpretive synthesis of the literature on access to healthcare by vulnerable groups. BMC Medical Research Methodology, 2006; 6: 35. 16872487 10.1186/1471-2288-6-35 PMC1559637

[15] Appiah B , Hong Y , Ory MG *et al* Challenges and opportunities for implementing diabetes self‐management guidelines. Journal of the American Board of Family Medicine: JABFM, 2013; 26: 90–92. 23288286 10.3122/jabfm.2013.01.120177

[16] Cardol M , Rijken M , van Schrojenstein Lantman‐de V . Attitudes and dilemmas of caregivers supporting people with intellectual disabilities who have diabetes. Patient Education & Counseling, 2012; 87: 383–388. 22178391 10.1016/j.pec.2011.11.010

[17] McDonald R , Rogers A , Macdonald W . Dependence and identity: nurses and chronic conditions in a primary care setting. Journal of Health, Organisation and Management, 2008; 22: 294–308. 10.1108/14777260810883558 18700586

[18] Shortus T , Kemp L , McKenzie S *et al* ‘Managing patient involvement’: provider perspectives on diabetes decision‐making. Health Expectations, 2013; 16: 189–198. 21645187 10.1111/j.1369-7625.2011.00700.x PMC5060650

[19] Asimakopoulou K , Newton P , Sinclair AJ *et al* Health care professionals’ understanding and day‐to‐day practice of patient empowerment in diabetes; time to pause for thought? Diabetes Research & Clinical Practice, 2012; 95: 224–229. 22036297 10.1016/j.diabres.2011.10.005

[20] Denford S , Frost J , Dieppe P *et al* Doctors’ understanding of individualisation of drug treatments: a qualitative interview study. BMJ Open, 2013; 3: e002706. doi:10.1136/bmjopen‐2013‐002706. 10.1136/bmjopen-2013-002706 PMC3657639 23793685

[21] Rogers A , Kennedy A , Nelson E *et al* Uncovering the limits of patient‐centeredness: implementing a self‐management trial for chronic illness. Qualitative Health Research, 2005; 15: 224–239. 15611205 10.1177/1049732304272048

[22] Bergsten U , Bergman S , Fridlund B *et al* “Delivering knowledge and advice”: healthcare providers’ experiences of their interaction with patients’ management of rheumatoid arthritis. International Journal of Qualitative Studies on Health and Well‐being, 2011; 6 DOI: 10.3402/qhw.v6i4.8473. 10.3402/qhw.v6i4.8473 PMC3206584 22053161

[23] Macdonald W , Rogers A , Blakeman T *et al* Practice nurses and the facilitation of self‐management in primary care. Journal of Advanced Nursing, 2008; 62: 191–199. 18394031 10.1111/j.1365-2648.2007.04585.x

[24] Schaevers V , Schoonis A , Frickx G *et al* Implementing a standardized, evidence‐based education program using the patient's electronic file for lung transplant recipients. Progress in Transplantation, 2012; 22: 264–270. 22951504 10.7182/pit2012366

[25] Gillibrand W , Taylor J , Hughes JG . Practice nurses’ views of their diabetes care. Practice Nursing, 2004; 15: 144–149.

[26] Hopp FP , Hogan MM , Woodbridge PA *et al* The use of telehealth for diabetes management: a qualitative study of telehealth provider perceptions. Implementation Science, 2007; 2: 14. 17475012 10.1186/1748-5908-2-14 PMC1867824

[27] Jones A , Pill R , Adams S . Qualitative study of views of health professionals and patients on guided self management plans for asthma. BMJ, 2000; 321: 1507–1510. 11118179 10.1136/bmj.321.7275.1507 PMC27554

[28] Moffat M , Cleland J , van der Molen T *et al* Poor communication may impair optimal asthma care: a qualitative study. Family Practice, 2007; 24: 65–70. 17158184 10.1093/fampra/cml062

[29] Ring N , Jepson R , Hoskins G *et al* Understanding what helps or hinders asthma action plan use: a systematic review and synthesis of the qualitative literature [Review]. Patient Education & Counseling, 2011; 85: e131–e143. 21396793 10.1016/j.pec.2011.01.025

[30] Schold AK , Ylikivela R , Lindstrom K *et al* The options of the management of self‐monitoring of blood glucose in primary health care centres by the diabetes nurses and patients. Primary Care Diabetes, 2013; 7: 143–149. 23415895 10.1016/j.pcd.2012.12.004

[31] Langstrup H . Making connections through online asthma monitoring. Chronic Illness, 2008; 4: 118–126. 18583449 10.1177/1742395308092480

[32] Jones MI , Greenfield SM , Bray EP *et al* Patient self‐monitoring of blood pressure and self‐titration of medication in primary care: the TASMINH2 trial qualitative study of health professionals’ experiences. British Journal of General Practice, 2013; 63: e378–e385. 23735408 10.3399/bjgp13X668168 PMC3662454

[33] Macneela P , Gibbons A , McGuire B *et al* “We need to get you focused”: general practitioners’ representations of chronic low back pain patients. Qualitative Health Research, 2010; 20: 977–986. 20335499 10.1177/1049732310364219

[34] Thille P , Ward N , Russell G . Self‐management support in primary care: enactments, disruptions, and conversational consequences. Social Science & Medicine, 2014; 108: 97–105. 24632054 10.1016/j.socscimed.2014.02.041

[35] Kennedy A , Rogers A , Bower P . Support for self care for patients with chronic disease. BMJ (British Medical Journal), 2007; 335: 968–970. 17991978 10.1136/bmj.39372.540903.94 PMC2071971

[36] Paterson B . Myth of empowerment in chronic illness. Journal of Advanced Nursing, 2001; 34: 574–581. 11380725 10.1046/j.1365-2648.2001.01786.x

[37] Thille PH , Russell GM . Giving patients responsibility or fostering mutual response‐ability: family physicians’ constructions of effective chronic illness management. Qualitative Health Research, 2010; 20: 1343–1352. 20530403 10.1177/1049732310372376

[38] Eldh AC , Ehnfors M , Ekman I . The meaning of patient participation for patients and nurses at a nurse‐led clinic for chronic heart failure. European Journal of Cardiovascular Nursing, 2006; 5: 45–53. 16014340 10.1016/j.ejcnurse.2005.06.002

[39] Brierley S , Eiser C , Johnson B *et al* Working with young adults with Type 1 diabetes: views of a multidisciplinary care team and implications for service delivery. Diabetic Medicine, 2012; 29: 677–681. 22375561 10.1111/j.1464-5491.2012.03601.x

[40] Jallinoja P , Absetz P , Kuronen R *et al* The dilemma of patient responsibility for lifestyle change: perceptions among primary care physicians and nurses. Scandinavian Journal of Primary Health Care, 2007; 25: 244–249. 17934984 10.1080/02813430701691778 PMC3379767

[41] Jeffrey JE , Foster NE . A qualitative investigation of physical therapists’ experiences and feelings of managing patients with nonspecific low back pain. Physical Therapy, 2012; 92: 266–278. 22173793 10.2522/ptj.20100416

[42] Jowsey T , Jeon YH , Dugdale P *et al* Challenges for co‐morbid chronic illness care and policy in Australia: a qualitative study. Australia & New Zealand Health Policy, 2009; 6: 22. 19735576 10.1186/1743-8462-6-22 PMC2745419

[43] Yohannes AM . General practitioners views and experiences in managing depression in patients with chronic obstructive pulmonary disease. Expert Review of Respiratory Medicine, 2012; 6: 589–595. 23234446 10.1586/ers.12.64

[44] Cass S , Ball L , Leveritt M . Australian practice nurses’ perceptions of their role and competency to provide nutrition care to patients living with chronic disease. Australian Journal of Primary Health, 2014; 20: 203–208. 23428207 10.1071/PY12118

[45] Chin MH , Cook S , Jin L *et al* Barriers to providing diabetes care in community health centers. Diabetes Care, 2001; 24: 268–274. 11213877 10.2337/diacare.24.2.268

[46] Ferrante JM , Piasecki AK , Ohman‐Strickland PA *et al* Family physicians’ practices and attitudes regarding care of extremely obese patients. Obesity, 2009; 17: 1710–1716. 19282824 10.1038/oby.2009.62 PMC2953252

[47] Löfman P , Pelkonen M , Pietilä A . Evaluation of self‐determination for patients with rheumatoid arthritis: starting points for the development of nursing practice [Finnish]. Hoitotiede, 2003; 15: 264–276.

[48] Peyrot M , Rubin RR . Access to diabetes self‐management education. Diabetes Educator, 2008; 34: 90–97. 18267995 10.1177/0145721707312399

[49] Beverly EA , Ritholz MD , Brooks KM *et al* A qualitative study of perceived responsibility and self‐blame in type 2 diabetes: reflections of physicians and patients. Journal of General Internal Medicine, 2012; 27: 1180–1187. 22549299 10.1007/s11606-012-2070-0 PMC3514987

[50] Lundh L , Rosenhall L , Tornkvist L . Care of patients with chronic obstructive pulmonary disease in primary health care. Journal of Advanced Nursing, 2006; 56: 237–246. 17042803 10.1111/j.1365-2648.2006.04027.x

[51] Granger BB , Sandelowski M , Tahshjain H *et al* A qualitative descriptive study of the work of adherence to a chronic heart failure regimen: patient and physician perspectives. Journal of Cardiovascular Nursing, 2009; 24: 308–315. 19465865 10.1097/JCN.0b013e3181a4be30

[52] Hunt LM , Arar NH . An analytical framework for contrasting patient and provider views of the process of chronic disease management. Medical Anthropology Quarterly, 2001; 15: 347–367. 11693036 10.1525/maq.2001.15.3.347

[53] Malone A , Davies M , Dempster M . Providing psychological services for people with diabetes. Practical Diabetes International, 2005; 22: 244–248.

[54] Ross J , Stefan H , Schauble B *et al* European survey of the level of satisfaction of patients and physicians in the management of epilepsy in general practice. Epilepsy and Behavior, 2010; 19: 36–42. 20638344 10.1016/j.yebeh.2010.06.002

[55] Carbone ET , Rosal MC , Torres MI *et al* Diabetes self‐management: perspectives of Latino patients and their health care providers. Patient Education & Counseling, 2007; 66: 202–210. 17329060 10.1016/j.pec.2006.12.003

[56] Detaille SI , Haafkens JA , Hoekstra JB *et al* What employees with diabetes mellitus need to cope at work: views of employees and health professionals. Patient Education & Counseling, 2006; 64: 183–190. 16469470 10.1016/j.pec.2005.12.015

[57] Junius‐Walker U , Voigt I , Wrede J *et al* Health and treatment priorities in patients with multimorbidity: report on a workshop from the European General Practice Network meeting ‘Research on multimorbidity in general practice’. European Journal of General Practice, 2010; 16: 51–54. 20184490 10.3109/13814780903580307

[58] Aliotta SL , Grieve K , Giddens JF *et al* Guided care: a new frontier for adults with chronic conditions. Professional case management, 2008; 13: 151–158. 18562909 10.1097/01.PCAMA.0000319968.76605.b3

[59] Kennedy A , Gask L , Rogers A . Training professionals to engage with and promote self‐management. Health Education Research, 2005; 20: 567–578. 15741189 10.1093/her/cyh018

[60] Abbott S , Burns J , Gleadell A *et al* Community nurses and self‐management of blood glucose. British Journal of Community Nursing, 2007; 12: 6–11. 17353805 10.12968/bjcn.2007.12.Sup3.23781

[61] Williams AM , Dennis S , Harris MF . How effective are the linkages between self‐management programmes and primary care providers, especially for disadvantaged patients? Chronic Illness, 2011; 7: 20–30. 20921035 10.1177/1742395310383339

[62] Day JL , Coles C , Walford S . Self‐management in diabetes: training implications for professional carers. Clinical Medicine, 2003; 3: 338–341. 12938748 10.7861/clinmedicine.3-4-338 PMC5351949

[63] Lake AJ , Staiger PK . Seeking the views of health professionals on translating chronic disease self‐management models into practice. Patient Education & Counseling, 2010; 79: 62–68. 19733460 10.1016/j.pec.2009.07.036

[64] McCann TV , Clark E . Advancing self‐determination with young adults who have schizophrenia. Journal of Psychiatric & Mental Health Nursing, 2004; 11: 12–20. 14723634 10.1111/j.1365-2850.2004.00645.x

[65] Proot IM , Abu‐Saad HH , Van Oorsouw GG *et al* Autonomy in stroke rehabilitation: the perceptions of care providers in nursing homes. Nursing Ethics, 2002; 9: 36–50. 16010896 10.1191/0969733002ne479oa

[66] Cavan DA . Structuring diabetes services to support self‐management. Practical Diabetes International, 2010; 27: 164–165.

[67] Brown A . Chronic leg ulceration in the community: changing the focus. British Journal of Community Nursing, 2010; (Suppl): (pp. S6, S8, S10 passim). 10.12968/bjcn.2010.15.sup5.78110 20852538

[68] Cleanthous S , Newman SP , Shipley M *et al* What constitutes uncertainty in systemic lupus erythematosus and rheumatoid arthritis? Psychology & Health, 2013; 28: 171–188. 22775405 10.1080/08870446.2012.701628

[69] Garrett DG , Martin LA . The Asheville Project: participants’ perceptions of factors contributing to the success of a patient self‐management diabetes program. Journal of the American Pharmacists Association: JAPhA, 2003; 43: 185–190. 10.1331/108658003321480722 12688436

[70] Nam S , Chesla C , Stotts NA *et al* Barriers to diabetes management: patient and provider factors [Review]. Diabetes Research & Clinical Practice, 2011; 93: 1–9. 21382643 10.1016/j.diabres.2011.02.002

[71] Simm R , Hastie L , Weymouth E . Is training in solution‐focused working useful to community matrons? British Journal of Community Nursing, 2011; 16: 598–603. 22413405 10.12968/bjcn.2011.16.12.598

[72] Catalano T , Kendall E , Vandenberg A *et al* The experiences of leaders of self‐management courses in Queensland: exploring Health Professional and Peer Leaders’ perceptions of working together. Health and Social Care in the Community, 2009; 17: 105–115. 19040700 10.1111/j.1365-2524.2008.00801.x

[73] Ford S , Schofield T , Hope T . Observing decision‐making in the general practice consultation: who makes which decisions? Health Expectations, 2006; 9: 130–137. 16677192 10.1111/j.1369-7625.2006.00382.x PMC5060340

[74] Gambling T , Long AF . The realisation of patient‐centred care during a 3‐year proactive telephone counselling self‐care intervention for diabetes. Patient Education and Counseling, 2010; 80: 219–226. 20006458 10.1016/j.pec.2009.11.007

[75] Rychetnik L , McCaffery K , Morton RL *et al* Follow‐up of early stage melanoma: specialist clinician perspectives on the functions of follow‐up and implications for extending follow‐up intervals. Journal of Surgical Oncology, 2013; 107: 463–468. 23090908 10.1002/jso.23278

[76] Fox A . Intensive diabetes management: negotiating evidence‐based practice. Canadian Journal of Dietetic Practice & Research, 2010; 71: 62–68. 20525416 10.3148/71.2.2010.62

[77] Kendall E , Catalano T , Kuipers P *et al* Recovery following stroke: the role of self‐management education. Social Science & Medicine, 2007; 64: 735–746. 17079060 10.1016/j.socscimed.2006.09.012

[78] Kendall E , Rogers A . Extinguishing the social?: state sponsored self‐care policy and the Chronic Disease Self‐management Programme. Disability & Society, 2007; 22: 129–143.

[79] Singleton JK . Nurses’ perspectives of encouraging clients’ care‐of‐self in a short‐term rehabilitation unit within a long‐term care facility. Rehabilitation Nursing Journal, 2000; 25: 23–30. 10.1002/j.2048-7940.2000.tb01852.x 10754924

[80] Collins S , Drew P , Watt I *et al* ‘Unilateral’ and ‘bilateral’ practitioner approaches in decision‐making about treatment. Social Science & Medicine, 2005; 61: 2611–2627. 16006027 10.1016/j.socscimed.2005.04.047

[81] Elliott N . ‘Mutual intacting’: a grounded theory study of clinical judgement practice issues. Journal of Advanced Nursing, 2010; 66: 2711–2721. 20722810 10.1111/j.1365-2648.2010.05412.x

[82] Grimaldi A . How to help the patient motivate himself? Diabetes & Metabolism, 2012; 38: S59–S64. 22541604 10.1016/S1262-3636(12)71536-7

[83] McLane L , Jones K , Lydiatt W *et al* Taking away the fear: a grounded theory study of cooperative care in the treatment of head and neck cancer. Psycho‐Oncology, 2003; 12: 474–490. 12833560 10.1002/pon.670

[84] Robinson A , Courtney‐Pratt H , Lea E *et al* Transforming clinical practice amongst community nurses: mentoring for COPD patient self‐management. Journal of Clinical Nursing, 2008; 17: 370–379. 10.1111/j.1365-2702.2008.02279.x 18557963

[85] Holmstrom I , Larsson J , Lindberg E *et al* Improving the diabetes‐patient encounter by reflective tutoring for staff. Patient Education & Counseling, 2004; 53: 325–332. 15186871 10.1016/j.pec.2003.12.013

[86] Hörnsten Å , Lundman B , Almberg A *et al* Nurses’ experiences of conflicting encounters in diabetes care. European Diabetes Nursing, 2008; 5: 64–69.

[87] Hale LA , Piggot J . Exploring the content of physiotherapeutic home‐based stroke rehabilitation in New Zealand. Archives of Physical Medicine & Rehabilitation, 2005; 86: 1933–1940. 16213234 10.1016/j.apmr.2005.04.011

[88] Holley UA . Social isolation: a practical guide for nurses assisting clients with chronic illness. Rehabilitation Nursing: The Official Journal of the Association of Rehabilitation Nurses, 2007; 32: 51–56. 17432633 10.1002/j.2048-7940.2007.tb00152.x

[89] Entwistle VA , Watt IS . Treating patients as persons: a capabilities approach to support delivery of person‐centered care. American Journal of Bioethics, 2013; 13: 29–39. 10.1080/15265161.2013.802060 PMC3746461 23862598

[90] Furler J , Spitzer O , Young D *et al* Insulin in general practice – barriers and enablers for timely initiation. Australian Family Physician, 2011; 40: 617–621. 21814661

[91] Johnson M , Newton P , Jiwa M *et al* Meeting the educational needs of people at risk of diabetes‐related amputation: a vignette study with patients and professionals. Health Expectations, 2005; 8: 324–333. 16266420 10.1111/j.1369-7625.2005.00344.x PMC5060318

[92] Kirby SE , Dennis SM , Bazeley P *et al* What distinguishes clinicians who better support patients for chronic disease self‐management? Australian Journal of Primary Health, 2012; 18: 220–227. 23069365 10.1071/PY11029

[93] Hughes JL . Chronic fatigue syndrome and occupational disruption in primary care: is there a role for occupational therapy? British Journal of Occupational Therapy, 2009; 72: 2–10.

[94] Clark M , Hampson SE . Comparison of patients’ and healthcare professionals’ beliefs about and attitudes towards Type 2 diabetes. Diabetic Medicine, 2003; 20: 152–154. 12581267 10.1046/j.1464-5491.2003.00896.x

[95] Kremer H , Bader A , O'Cleirigh C *et al* The decision to forgo antiretroviral therapy in people living with HIV compliance as paternalism or partnership? European Journal of Medical Research, 2004; 9: 61–70. 15090291

[96] Greiner KA . Patient‐provider relations–understanding the social and cultural circumstances of difficult patients. Bioethics Forum, 2000; 16: 7–12. 12528721

[97] Thomson D . An ethnographic study of physiotherapists’ perceptions of their interactions with patients on a chronic pain unit. Physiotherapy Theory & Practice, 2008; 24: 408–422. 19117232 10.1080/09593980802511805

[98] Ritholz MD , Beverly EA , Brooks KM *et al* Barriers and facilitators to self‐care communication during medical appointments in the United States for adults with type 2 diabetes. Chronic Illness, 2014; 10: 303–313. 24567195 10.1177/1742395314525647 PMC4157962

[99] Zakrisson AB , Hagglund D . The asthma/COPD nurses’ experience of educating patients with chronic obstructive pulmonary disease in primary health care. Scandinavian Journal of Caring Sciences, 2010; 24: 147–155. 19691488 10.1111/j.1471-6712.2009.00698.x

[100] Jutterstrom L , Graneheim U , Isaksson U *et al* Ideal versus real conditions for Type 2 diabetes care: diabetes specialty nurses’ perspectives. Internet Journal of Advanced Nursing Practice, 2012; 11: 1–1.

[101] Kennedy AP , Rogers AE . Improving patient involvement in chronic disease management: the views of patients, GPs and specialists on a guidebook for ulcerative colitis. Patient Education & Counseling, 2002; 47: 257–263. 12088604 10.1016/s0738-3991(01)00228-2

[102] South J , Darby F , Bagnall A *et al* Implementing a community‐based self care training initiative: a process evaluation. Health & Social Care in the Community, 2010; 18: 662–670. 20637043 10.1111/j.1365-2524.2010.00940.x

[103] Clark NM , Stoll S , Youatt EJ *et al* Fostering epilepsy self management: the perspectives of professionals. Epilepsy & Behavior, 2010; 19: 255–263. 21145491 10.1016/j.yebeh.2010.08.033

[104] Blakeman T , Macdonald W , Bower P *et al* A qualitative study of GPs’ attitudes to self‐management of chronic disease. British Journal of General Practice, 2006; 56: 407–414. 16762121 PMC1839014

[105] Bower P , Macdonald W , Harkness E *et al* Multimorbidity, service organization and clinical decision making in primary care: a qualitative study. Family Practice, 2011; 28: 579–587. 21613378 10.1093/fampra/cmr018

[106] Day M , McCarthy G , Leahy‐Warren P . Professional social workers’ views on self‐neglect: an exploratory study. British Journal of Social Work, 2012; 42: 725–743.

[107] Norris M , Jones F , Kilbride C *et al* Exploring the experience of facilitating self‐management with minority ethnic stroke survivors: a qualitative study of therapists’ perceptions. Disability and Rehabilitation, 2014; 36: 2252–2261. 24670190 10.3109/09638288.2014.904936 PMC4364271

[108] Norris M , Kilbride C . From dictatorship to a reluctant democracy: stroke therapists talking about self‐management. Disability & Rehabilitation, 2014; 36: 32–38. 23594054 10.3109/09638288.2013.776645 PMC3906249

[109] Richards DA , Lankshear AJ , Fletcher J *et al* Developing a U.K. protocol for collaborative care: a qualitative study. General Hospital Psychiatry, 2006; 28: 296–305. 16814628 10.1016/j.genhosppsych.2006.03.005

[110] Richards G , Morris M , Booker S *et al* What do people with type 1 diabetes find helpful in health professionals? Results from a focus group study. Practical Diabetes International, 2006; 23: 249–252.

[111] Brez S , Rowan M , Malcolm J *et al* Transition from specialist to primary diabetes care: a qualitative study of perspectives of primary care physicians. BMC Family Practice, 2009; 10: 39. 19500397 10.1186/1471-2296-10-39 PMC2704171

[112] Hajos TRS , Polonsky WH , Twisk JWR *et al* Do physicians understand Type 2 diabetes patients’ perceptions of seriousness; the emotional impact and needs for care improvement? A cross‐national survey. Patient Education and Counseling, 2011; 85: 258–263. 20932702 10.1016/j.pec.2010.08.019

[113] Adolfsson ET , Smide B , Gregeby E *et al* Implementing empowerment group education in diabetes. Patient Education & Counseling, 2004; 53: 319–324. 15186870 10.1016/j.pec.2003.07.009

[114] Dures E , Hewlett S , Ambler N *et al* Rheumatology clinicians’ experiences of brief training and implementation of skills to support patient self‐management. BMC Musculoskeletal Disorders, 2014; 15: 108. 24678645 10.1186/1471-2474-15-108 PMC3978089

[115] Kosmala‐Anderson JP , Wallace LM , Turner A . Confidence matters: a Self‐Determination Theory study of factors determining engagement in self‐management support practices of UK clinicians. Psychology Health & Medicine, 2010; 15: 478–491. 10.1080/13548506.2010.487104 20677086

[116] Newton P , Sasha S , Koula A . Marrying contradictions: healthcare professionals perceptions of empowerment in the care of people with type 2 diabetes. Patient Education & Counseling, 2011; 85: e326–e329. 21530141 10.1016/j.pec.2011.03.015

[117] Rohde A , Townley‐O'Neill K , Trendall K *et al* A comparison of client and therapist goals for people with aphasia: A qualitative exploratory study. Aphasiology, 2012; 26: 1298–1315.

[118] Bodenheimer T , Lorig K , Holman L *et al* Patient self‐management of chronic disease in primary care. JAMA, 2002; 288: 2469–2475. 12435261 10.1001/jama.288.19.2469

[119] Protheroe J , Brooks H , Chew‐Graham C *et al* ‘Permission to participate?’ A qualitative study of participation in patients from differing socio‐economic backgrounds. Journal of Health Psychology, 2012; 18: 1046–1055. 23104997 10.1177/1359105312459876

[120] Kennedy A , Rogers A , Chew‐Graham C *et al* Implementation of a self‐management support approach (WISE) across a health system: a process evaluation explaining what did and did not work for organisations, clinicians and patients. Implementation Science, 2014; 9: 129. 25331942 10.1186/s13012-014-0129-5 PMC4210530

[121] Entwistle VA , Firnigl D , Ryan M *et al* Which experiences of healthcare delivery matter to service users and why? A critical interpretive synthesis and conceptual map. Journal of Health Services Research and Policy, 2012; 17: 70–78. 21967821 10.1258/jhsrp.2011.011029 PMC3336938

