# Supplementary material for: We need to talk about purpose: a critical interpretive synthesis of health and social care professionals’ approaches to self‐management support for people with long‐term conditions
Source: Health Expect. 2016 Apr 14;20(2):243–59. doi: 10.1111/hex.12453 (PMC5354019; doi:10.1111/hex.12453)
Supplement: Supplementary file 1 — Table S1. Annotated bibliography. [file HEX-20-243-s001.docx]

**Annotated bibliography**

|  | **Author date** | **Professional group(s) studied (if applicable)**  **Conditions/settings of interest**  **Country** | **Study aim**  **Main method of data collection/other approach** | **What we found most useful *(and reflected on)* in the context of this project** |
| --- | --- | --- | --- | --- |
|  | Abbott 2007 a^1,^ | Community and district nurses  Diabetes (type 2)  England | To examine how nurses who treat or manage type 2 diabetes in primary care settings perceive the purposes and functions of self-testing of blood glucose levels  Interviews | Notes that nurses gave no indication of patients being involved in decisions about their treatment, suggesting that patients could only choose to comply or not comply with treatment recommendations. Reports that nurses highlighted the practical difficulties of self-testing for people with limited eyesight or dexterity, and expressed concerns about the point and effectiveness of self-testing, especially if patients were not able or confident to use the test results to inform self-care decisions, or if blood glucose control was stable and habits of daily living regular. Highlights the danger that self-testing is seen as a proxy for patient engagement, and that the incorporation of self-testing within narrow biomedically-oriented practice becomes a means to generate compliance rather than to support deeper forms of self-management or more genuine self-reliance. Acknowledges that community nurses are ‘caught … between policy imperatives of patient choice and evidence-based medicine’. Links their position to longstanding philosophical questions about whether, to be considered empowered, people must do the ‘right’ thing. |
|  | Abbott 2007b^2^ | Aboriginal health workers, GPs  Chronic disease (various)  Australia | To discuss experiences of using patient-reported health and self-management measures  Pilot study, interviews | Describes experiences of using and adapting questions that assess patients’ self-reported health and knowledge and abilities relating to self-management. Notes that while the questions were developed to facilitate communication between health professionals and patients, and to evidence changes in self-management, the team modified them and used them to try to uncover previously hidden barriers that were preventing patients from taking control of their own health. Reports that Aboriginal Health Workers initially found the questions hard to use, but thought that asking them helped them to understand the barriers that some of their clients faced to effective chronic disease self-management. Reports that this understanding motivated the Aboriginal Health Workers to provide ongoing assistance. |
|  | Adolfsson 2004^3^ | Doctors and nurses  Diabetes, primary care  Sweden | To gain insight into experiences of implementing (after training) an empowerment group education approach  Focus groups | Reports that health professionals who had run at least one educational group for patients in accordance with the empowerment approach they had been trained in experienced a conflict between their traditional roles as physicians or nurses in individual counselling and the roles required by the empowerment approach. In part this reflected a shift between an expert and a facilitator role. They considered the empowerment approach to be right (and some said it was not new to them) but difficult – there was a difference between having a theoretical understanding of an approach and being readily able/accustomed to use it in practice. They suggested the shift in practice required making multiple changes and managing many new features of practice at once. Notes that a lack of time in practice, and the need to work with colleagues who were not all following an empowerment approach, were experienced as barriers to implementation. |
|  | Albert 2008^4^ | N/A    Heart failure  USA | To summarise what is known about self-care from the perspectives of healthcare providers  ‘State of the field’ review | Recognises the evidence linking self-care (including adherence to medication, diet and exercise regimes) to reduced morbidity and mortality in heart failure, and highlights the lack of evidence about the effectiveness of health professionals’ actions to support self-care. Notes that while performance measurement usually involves assessment of health professionals’ documentation of the education they have given, this ‘may not equal systematic delivery of self-care education and skill acquisition’. Notes limitations in professional knowledge of self-care principles and advocates the development and use of tools that assess patients’ adherence to and compliance with various self-care behaviours over time. |
|  | Aliotta 2008^5^ | Nurses  Chronic conditions  (various)  USA | To develop and evaluate a ‘Guided Care’ model of case management for people with chronic conditions  Descriptive, with reference to a pilot study | Implicitly highlights potential tensions in the provision of support: some aspects of the Guided Care (GC) model seek to individualise care plans for patients (e.g. by asking them about their own priorities/values, etc.), but other aspects apparently seek to coach and motivate patients to act in specific and similar ways. Also, the authors state that because GC nurses assess the patient’s home and family caregiver(s) in detail, they often have a deep understanding of personal issues and family dynamics that may support adherence or contribute to non-adherence to the care guide and the action plan. However, they do not indicate how GC nurses might manage tensions between people’s personal issues and adherence to specific action plans. Some features of the GC program as described suggest an inflexibility and potential insensitivity to patients’ concerns (e.g. the lay version of the care plan is ‘displayed prominently in the patient’s home’). |
|  | Appiah 2013^6^ | Various health care professionals and leaders  Diabetes  USA | To examine perspectives on the implementation of diabetes self-management guidelines  Group discussions | Identifies four key challenges to implementing evidence-based self-management guidelines: (1) health professionals’ lack of electronic information re patients; (2) inadequate coordination between providers; (3) conflicting interpretation of guidelines; and (4) cost disincentives for providers to devote time to educating patients about self-management. Calls for ‘improving electronic medical record systems to allow better coordination between providers and across institutions, engaging more health educators and nurses in educating patients about diabetes self-management, mobilising community resources to disseminate the guidelines, and incorporating quality of care into incentives for providers.’ |
|  | Asimakopoulou 2012^7^ | Various health care professionals  Diabetes  UK | To examine understandings, attitudes and practices relating to ‘empowerment’  Interviews | Reports that health care professionals generally accepted the concept of empowerment positively and wished to implement it in practice, but also that they recognised it could be understood in different ways. There was some consensus that it involved educating and imparting knowledge and skills to patients, and equipping patients to ‘successfully manage’ their condition. However, health professionals varied in terms of what they took as indicators of success: some focused on biochemical targets, others added quality of life into the mix, and one saw it as dependent on the individual patient’s perspective. All participants could identify some patients who were resistant to being empowered in any traditional sense of the concept. |
|  | Bancroft 2008^8^ | N/A  Diabetes  England | Industry sponsored didactic/opinion piece | Serves as an example of how advice to health professionals can oversimplify the challenges of supporting people with long-term conditions. Outlines the size of the English population with diabetes and asserts the benefits of good glycaemic control. Offers reasons why many patients do not meet glycaemic control targets – including lack of understanding, false sense of security, lack of concern, ‘difficulty in meeting lifestyle targets’ and reluctance to take insulin. Discusses insulin therapy and new insulin analogues, highlighting features that ‘make[s] them attractive to patients who are trying to minimise disruption to their lifestyle’. Expresses confidence that ‘Putting patients in control of their insulin can help them to ... make the transition to insulin therapy a positive step’. Concludes ‘RCN guidance reminds nurses that their role is only to explain the option – ultimately the decision is the patient’s’. [We note that the only self-management options discussed in this article are different forms of insulin, and that the article neglects to consider what health professionals should do if patients don’t want to take insulin (even after an optimistic presentation of information about the latest products) and/or cannot meet glycaemic control targets.] |
|  | Bergsten 2011^9^ | Rheumatologists, occupational and physio- therapists, social workers, nurses  Rheumatoid arthritis  Sweden | To explore healthcare providers’ experiences of their interactions with patients’ management of rheumatoid arthritis  Individual interviews | Reports that healthcare providers saw their core task as the provision of knowledge and advice about the condition and appropriate treatment. They aimed to involve and motivate patients to be active in their own care, and ‘adjusted’ delivery in various ways. But they saw themselves as experts or teachers as well as person centred guides, and experienced frustration when patients did not take responsibility for [managing] their condition, or were non-compliant. Also notes that health professionals experienced frustration when they could not satisfy the high expectations/demands of some patients who did take responsibility for managing their condition. |
|  | Beverly 2012^10^ | Endocrinologists and primary care physicians  Diabetes (type 2)  USA | To explore physicians’ and patients’ views of patients’ difficulties achieving diabetes treatment goals  Interviews | Highlights important differences between physicians’ and patients’ views on patients’ difficulties achieving treatment goals. Reports that physicians are aware that patients struggle to reach treatment goals, see themselves as responsible for this perceived failure and are concerned they might not be doing enough to help, but are uncertain how to help patients better. Reports that patients, meanwhile, tend to blame themselves, seeing their inability to carry out recommended self-care behaviours as the reason why they don’t achieve treatment goals. Suggests both physicians and patients may benefit from better understanding each other’s challenges and frustrations. |
|  | Bhattacharyya 2011^11^ | Nurses, doctors and community health representatives  Diabetes,  First Nations  Canada | To explore how health care providers rate the barriers to providing optimal diabetes care  Interviews, focus groups and questionnaires | Reports that health care providers rated: (1) ‘Motivate patients to adopt healthy lifestyles’; (2) ‘Help patients to seek preventive care’; and ‘(3) Improve adherence to medication’ as the strategies most likely to have a large impact on improving diabetes care in First Nations communities. Suggests that this rating might reflect some ‘patient blaming,’ but notes too that strategies of supporting self-management (including reorganisation of clinics and training for community health representatives), may also be well-suited for diabetes care in First Nations contexts. |
|  | Bieber 2006^12^ | Hospital doctors in outpatient clinics  Fibromyalgia syndrome  Germany | To investigate the effects of a shared decision-making intervention  Combined qualitative and quantitative follow up of intervention trial participants | Describes how people with fibromyalgia syndrome and their doctors frequently complain about interaction difficulties. The shared decision-making intervention focussed on ‘building a good working alliance with the patient by analysing deliberate as well as unconscious signals of verbal and non-verbal communication, reflecting, and adequately reacting to them.’ Reports that patients and doctors in the shared decision-making intervention group reported significantly better physician-patient interaction, and patient coping. Suggests that a shared decision-making intervention can lead to an improved physician-patient relationship from both perspectives. |
|  | Blakeman 2006^13^ | GPs  Chronic conditions (various)  UK | To explore GP perspectives on their involvement in the facilitation of chronic disease self-management  Interviews | Highlights the tensions and trade-offs that GPs experienced and made regarding their role in facilitating self-management. Although GPs valued increased patient involvement and increased self-management, they reported significant barriers to the achievement of these, including: lack of effective consultation skills to facilitate patient involvement; conflict between values of professional responsibility and increased patient control; and contextual barriers, such as incentive schemes, that focus on biomedical care. Suggests GPs’ ability to facilitate self-management depends on the relationship between the content of the consultation, the values the GPs prioritise, and the context in which chronic disease management is organised. Argues that the context in which GPs work may make them more likely to prioritise the biomedical aspects of care at the expense of having space to explore the patient’s perspective. |
|  | Blakeman 2010^14^ | GPs, practice nurses, nurse practitioner, assistant health practitioner  Long-term conditions  England | To explore self-management support in primary care consultations  Consultation recordings, interviews (in which recordings were reviewed with professionals), interviews with patients | Identifies tensions in addressing self-management behaviours in consultations while not disrupting professional-patient relations. Suggests that the presentation and protection of ‘selves’ and the maintenance of self-other relations were key concerns for both patients and professionals, and that the introduction of self-management topics could threaten this process. Illustrates how concerns not to exacerbate the disruption of living with long-term conditions can discourage both professionals and patients from a focus on managing them – and how professionals can sense a need for a robust enough relationship to bring self-management behaviours into view in discussions. Shows how the caution used to introduce self-management topics, including in the course of template-structured review consultations, could minimise scope for the expansion of dialogue about self-management and create pressure to rely on biomedical considerations. Observes how a division of labour within practices that allocates self-management work to nurses does not necessarily resolve professional identity concerns for either GPs or nurses. |
|  | Bower 2011^15^ | GPs and practice nurses  Multimorbidity, primary care  England | To explore GP and nurse perceptions of multimorbidity and its influence on service organisation and clinical decision making  Interviews | Reports that the tensions health professionals experience between meeting quality targets and fulfilling patients’ agendas can be exacerbated in multimorbidity. People with multiple conditions can face particular challenges (e.g. to understand the relationships between conditions and to deal with the impact of particular behaviours on several conditions, sometimes in the face of cognitive decline and a feeling of being overwhelmed). Support for self-management needs to address problems of polypharmacy and interactions between self-management behaviours. Service fragmentation can add to patients’ burden, so efforts by providers to make service delivery easier on the patient (e.g. by co-ordinating appointments) can be helpful. |
|  | Brez 2009^16^ | Primary care physicians  Diabetes  Canada | To explore perspectives on responsibility for the care of patients referred to a diabetes specialist centre  Focus groups | Highlights the complex interdependencies associated with transitions in diabetes care and underlines the breadth of concerns primary care physicians can have when patient care is transferred back to them from specialist clinics (especially after long periods of time). Illustrates that primary care physicians can be reluctant to embrace models of self-management that move beyond a narrow model of compliance with biomedical instruction, and links this to a lack of confidence and competence. Suggests that when patients are referred between health professionals, they can experience different expectations about, and support for, what they do for themselves. Also, policies and system rules may mean that the costs to patients of accessing supplies for self-management vary across providers – so the kinds of practical support that some providers can give is more limited than that of others. |
|  | Brierley 2012^17^ | Doctors, nurses, dieticians, clinical psychologist  Diabetes (type 1)  England | To describe the challenges of working with young adults with type 1 diabetes to inform an age-appropriate intervention  Interviews | Highlights the emotional burden that staff can experience when working with young adults with type 1 diabetes. Notes that this can be associated with the low priority some young adults give to self-care and the frustration and helplessness staff feel in relation to this, as well as with the general difficulties of managing type 1 diabetes. Mentions staff uncertainty about how far to inform young adults about the possible future consequences of accepting suboptimal glycaemic control for the sake of current quality of life, and staff concern that young adults will not always be fully open with them about their self-care. Reports that some staff use pejorative terms to categorise patients as good or bad, based on the adequacy of their HbA_1c_ readings. |
|  | Brown 2010^18^ | N/A  Chronic leg ulceration  UK | To appraise support for self-management interventions for chronic leg ulceration  Literature review with reflection on policy and practice | Expresses concern that when the [sole] indicator of quality of nursing care for venous leg ulcers is ulcer healing, nurses’ other contributions to quality of life tend to be neglected, and that this may lead to a harmful service reductions, particularly for people for whom complete leg ulcer healing is not a realistic goal. Comments ‘It would be unthinkable to treat a patient with multiple sclerosis within the medical model, focusing on cure as a treatment outcome in the knowledge that a key feature of the disease trajectory is the periods of steady decline followed by episodes of temporary remission’. Suggests that chronic venous leg ulceration should be viewed as a chronic condition (in a way that it is not currently), that treatment and support for self-management should be tailored to the patient’s lifestyle, and that enablement of successful adaptation to life with an ulcer should be an alternative goal to complete healing, especially when healing isn’t occurring and/or re-ulceration occurs. |
|  | Carbone 2007^19^ | Primary care providers  Diabetes (type 2)  USA | To inform the refinement of self-management interventions tailored to Latino patients with type 2 diabetes  Focus groups (health professionals, patients) | Reports that practitioners expressed strong commitment to encourage patients’ self-management practices and acknowledged their role in the lives of their patients, especially when they had longstanding relationships. Identifies significant discrepancies between Latino patients’ and practitioners’ perspectives, including on whether and how it is possible to have ‘normal lives’ with diabetes, how manageable diabetes is, and how important family support is. Reports that practitioners did not always use a goal-setting form that was routinely given to patients on diagnosis, sometimes because they did not think patients could cope with the concept of goal setting. Also notes that when they did use the goal-setting form, they tended to focus on longer term, directive goals. |
|  | Cardol 2012^20^ | Professional caregivers  Diabetes, intellectual disability  Netherlands | To explore how caregivers in communal living arrangements support people with mild or moderate intellectual disabilities and diabetes  Semi-structured interviews | Indicates that professional caregivers’ approaches to supporting self-management were influenced by their views of the severity of diabetes and of patients’ motivation, capacity to learn and trustworthiness. Reports that, in general, ‘support was more directed towards control and prohibition than towards developing self-efficacy and autonomy’, but does identify some examples of strategies for facilitating self-management via trust and a structured developmental approach. Notes that staff experienced dilemmas, for example between pursuing good diabetes care and person-centred care (especially for food intake) or between health (indicators of diabetes) and quality of life. Suggests that ‘self-management may be too closely associated with independence’ and that this does not fit with the reality of human life, especially for people with intellectual disability. |
|  | Cass 2014^21^ | Primary care practice nurses  Chronic disease  Australia | To investigate nurses’ perceptions of their role and competency to provide nutrition care to people living with chronic disease  Interviews | Reports that nurses saw nutrition care (general advocacy of the importance of diet, plus specific nutrition-related assessment and basic advice) as an important part of their role in chronic disease management, although they were aware of professional boundaries and careful not to overstate their role in relation to other health professionals. Their interpretations of what counted as ‘basic’ advice varied. Many thought that nurses had non-judgemental and empathetic attitudes, and that patients trusted them and considered them approachable. They further thought that this meant they were well positioned to provide nutrition care. Some quotations however, suggest the nurses formed negative judgements of some patients’ honesty and willingness to listen. Highlights the range of competencies required for nutrition care and notes that a lack of nurses’ time, knowledge and confidence can also be barriers. |
|  | Catalano 2009^22^ | Health professional and (lay) peer self-management trainers  Various long-term conditions  Australia | To describe the experiences of volunteers trained to deliver the Stanford Chronic Disease Self-management Program course  Questionnaires followed by interviews | Acknowledges disparities between the philosophy of self-management and experiences of the delivery of self-management training courses, as well as parallels between what is needed for health professional leaders and peer leaders to work well together as training providers and what is needed for health professionals’ support for self-management more generally. Identifies two core themes that describe leaders’ perceptions and experiences of their working relationships: (1) the value of working together (combining the best of both world perspectives) and (2) relationship tensions (peer leaders were sometimes undervalued and experienced a lack of ownership, voice and status). Notes that being given either too much or too little responsibility could be problematic, and that responsibilities needed to be negotiated. Reports that when peer leaders described positive and equal interactions with health professionals, they were based on mutual respect and understanding, clear communication and expectations, and willingness to learn from each other. |
|  | Cavan 2010^23^ | Consultants, GPs, nurse specialists, dieticians, clinical psychologists and managers  Diabetes  England | To explore key requirements for structuring diabetes services to support self-management  ‘Think tank’ discussion | Reports practice-informed recommendations that stress a need for flexibility at several levels. These include: (1) What’s needed for patients should include: (a) effective education that is seen as part of treatment and that sets up an expectation of need to learn to manage condition and problem solve; (b) flexible access to information, support, treatment, equipment etc. when needed; and (c) support for educational and medical goal setting oriented to help them help themselves; (2) Training for health professionals should recognise that some will need to change mind-sets to think to ask patients ‘how can I help you to help yourself?’ and to ‘respect choices made without judgement’; and (3) A system that emphasises well-defined care pathways might not support the flexibility of access required. Patients might need support with service navigation from their first point of contact. |
|  | Chin 2001^24^ | Health care providers and administrators  Diabetes  USA | To identify barriers to improving care for individuals with diabetes in community health centres  Questionnaires | Reports that providers considered patients significantly less likely than health professionals to believe that some processes of care were important. Also, providers expressed more confidence in their ability to instruct patients on diet and exercise than on their ability to help them make changes in these areas. About 25% of respondents agreed the following were significant barriers to good care: affordability of home blood glucose monitoring, HbA_1c_ testing, dilated eye examination, and special diets; non-proximity of ophthalmologist; forgetting to order eye examinations and to examine patients’ feet; time required to teach home blood glucose monitoring; and language or cultural barriers. Acknowledges that ‘Diabetes care is complex because it involves both self-care by the patient and the administration of key processes of care by the provider’. Seems to regard poor compliance/adherence by patients primarily as a barrier to the quality of professionally delivered care. |
|  | Ciccone 2010^25^ | Care managers and referring general and specialist doctors  Heart failure and diabetes  Italy | To evaluate the impact of a disease and care management model and the introduction of trained ‘care manager’ nurses into a primary health care system  Feasibility and evaluation report (methods unclear) | Reports that the introduction of nurses as ‘care managers’ to act as a liaison between physician and patient, monitor measures and coordinate appointments, etc. was practical, feasible and apparently associated with improved health and self-management outcomes as well as improved patient confidence and safety. Suggests this was achieved through: (1) increasing patients’ health knowledge, self-management skills and readiness to make changes in health behaviours; (2) ongoing monitoring by the health care team (which helped to promote confidence and enhance safety of chronic patient management at home); and (3) strong ‘partnership’ between the care manager and patient, and collaboration between the care manager and physicians. Recommends that patient should be considered the most important member of the team and the care manager should be seen as a key health care collaborator to enhance and support services from physicians in primary health care settings. |
|  | Clark 2003^26^ | Nurses, GPs and diabeticians  Diabetes (type 2)  UK | To compare patients’ and healthcare professionals’ beliefs and attitudes towards diabetes  Questionnaires using a validated measure | Reports that healthcare professionals generally viewed type 2 diabetes as more serious than their patients did. Most of the healthcare professionals considered diabetes harder to treat than other chronic conditions and felt they did not have adequate time and resources to treat people with diabetes effectively. Suggests that practice needs to shift from an authoritative model in which healthcare professionals attempt to dictate to patients to a more collaborative alliance. Concludes that in forming collaborative alliances, healthcare professionals need to recognise differences between practitioner and patient perspectives. |
|  | Clark 2010^27^ | Recognised experts (clinicians, social service providers and others)  Epilepsy  USA | To determine how recognised experts view the challenges facing people with epilepsy and the potential to assist with self-management  Interviews (telephone) | Notes particular features of epilepsy and its social consequences that have important implications for patients’ ability and willingness to communicate with clinicians about their experiences. Points out that it can be hard for clinicians to get an accurate picture of a person’s epilepsy, response to particular treatments and need for different or additional treatment or support because: people who fear losing a driving license might be reluctant to report seizure activity; people might not realise they have had a seizure, or forget how many seizures they have had; epilepsy is associated with cognitive problems and people might not understand the condition well enough to know what it is relevant to report; people might not think (it appropriate) to report what they see as non-medical issues. |
|  | Cleanthous 2013^28^ | Various, including rheumatologists, specialist nurses, physiotherapist, psychologist  Systemic lupus erythematosus and rheumatoid arthritis  England | To explore the aspects of uncertainty experienced by people with systemic lupus erythematosus and rheumatoid arthritis  Interviews | Illustrates the various senses in which people with long-term conditions can experience uncertainty about those conditions and their management (including about the health professionals involved in their management), and considers the implications of uncertainty in a number of domains of life. Shows how some of the ways that patients express or deal with uncertainty can have unwanted or avoidable negative implications for condition management, professional-patient relations or quality of life issues. Suggests that support to manage uncertainty could be helpful but that not all domains of uncertainty or forms of support fall within the conventional bounds of health care. Reports that the health professionals in this study were aware of the broad kinds of uncertainty that people experienced, but does not discuss what they did (or could do) about these. |
|  | Collins 2005^29^ | GPs [and hospital consultants]  Diabetes [head and neck cancers]  England | To examine health professionals’ decision-making talk and consider the implications for patients’ participation  Conversation analysis of recorded consultations | Illustrates how health professionals’ management of communication can be more unilateral (structured somewhat independently of the patient) or more bilateral (structured in negotiation with the patient). Identifies key features of more unilateral and more bilateral approaches, including that the latter: begin from an accommodation of the patient’s perspective; invite patients to express their view; incorporate patient’s understanding into situational assessments; present decisions as ‘to be made’ (rather than as news or decisions already made of necessity); cover relatively more options; signpost and introduce options in a staged way; and separate the presentation of options from the decision point. |
|  | Coventry 2014^30^ | GP, practice nurses  Multimorbidity, primary care  UK | To explore patient and health professional perspectives on factors influencing patient engagement with self-management in the context of multimorbidity  Interviews (with patients as well as practitioners) | Identifies three main factors that influence patients’ engagement with self-management: capacity; responsibility; and motivation. Suggests that all three can be limited by both multimorbidity and socio-economic deprivation. Notes some differences in practitioner and patient perspectives. Practitioners’ concerns about capacity (a) focused on the time and energy required to deal with illness and on combinations of physical incapacity that precluded some forms of self-management and made it hard for practitioners to advise about exercise and (b) noted that social isolation and poor access to material resources including transport options could make it hard for people in deprived areas in particular to learn about self-management and get to support groups. In contrast, some patients (a) ‘had greater interpretative capacity… to spot opportunities to maximise the benefits of self-management for all their health problems’ (b) spoke about their ‘capacity to cope’ being sustained by social and emotional support and (c) highlighted how financial and other daily anxieties could limit their emotional capacity to engage in self-management [of their conditions]. Suggests improving interpretive capacity to recognise potential gains could lead to greater (patient) responsibility and motivation. Implicitly recognises different interpretations of ‘self-management’ (especially among patients) but talks in terms of ‘successful’ or ‘full’ self-management without examining what these would look like. |
|  | Day 2012^31^ | Doctors and nurses experienced in diabetes care  Diabetes  UK | To examine factors influencing patient self-management and implications for those providing care  Workshop discussion | Summarises research evidence about the importance of (ongoing) patient education to ensure understanding, and of attention to psychological aspects of self-management. Notes that the education of health professionals has led them to focus on solving other people’s problems for them rather than helping people to solve their own problems. Stresses that all health professionals working with people with diabetes need (training to acquire) knowledge of relevant psychological issues and skills of listening, negotiating etc. to support patients’ critical empowerment and self-management. |
|  | Day 2003^32^ | Social care workers  Self-neglect  Ireland | To explore the views and experiences of social care workers on self-neglect  Interviews | Explains that self-neglect is sometimes but not always associated with (other) long term health conditions. Reports that social workers find this complex and multidimensional phenomenon challenging and frustrating. Reports that all participants saw home visits and the establishment of a therapeutic relationship as essential to engage with people who self-neglect. They saw sensitivity, respect and a principle of non-maleficence as important but noted the practical and ethical challenges of balancing respecting people’s choices, autonomy and self-determination with judgement of risk and the establishment of a safe living environment. Some participants thought that if a person who self-neglected and had capacity resisted intervention by choice, they must step back and wait for a crisis to occur. |
|  | Delea 2010^33^ | Pharmacy students  Diabetes  USA | To assess students’ attitudes to diabetes and self-management before and after participation in a week-long simulation of ‘Living With Diabetes’  Questionnaires | Shows that health professionals’ attitudes can change with encouragement to engage with what their patients actually experience. Reports a number of shifts in students’ attitudes relating to the self-management of diabetes and their ability to educate patients. Most of these shifts were probably positive (e.g. there was increased recognition of the psychosocial impact of diabetes, and that patients with diabetes should have autonomy regarding treatment), but the students (who had been asked to inject normal saline and maintain a blood glucose log for a week) were less likely after the intervention than before it to think that tight glucose control ‘is too much work’. |
|  | Denford 2013^34^ | GPs, geriatricians and clinical academics  Various long-term conditions  England | To explore doctors’ understanding of the ‘individualisation’ of drug treatments, and identify how they achieved individualisation  Interviews | Illuminates the different but often limited views that doctors hold about individualisation. When asked about individualisation, few doctors talked about how patients might modify their own treatment regimens as they used their medicines outside of consultations. Doctors generally supported patients with instructions and reminders oriented to promote the use of medicines as prescribed. They saw the individualisation of prescribing decisions as a matter of *either* matching the patient to the relevant guideline *or* prescribing against the guideline according to a particular patient’s beliefs, wishes and/or willingness. The particular examples doctors described from their practice did not always match the understanding of individualisation they described in general terms. |
|  | Detaille 2006^35^ | GPs, occupational physicians, others  Diabetes  Netherlands | To explore and compare the ideas of employees with diabetes and health professionals about what support diabetic patients need in work situations  Qualitative concept mapping sessions | Illustrates how health professionals’ views about support for diabetes management can differ from those of patients. In broad terms, health professionals and employees with diabetes identified a similar range of things that people with diabetes need to be able to keep working. However, the health professionals put more emphasis on technical aspects of managing the biomedical condition (e.g. regulating blood sugar) and did not reflect the employees’ broader concerns about accepting and coping with the disease emotionally, living normally, being understood by colleagues, and knowing how to finance technical devices. The health professionals also ranked support from health professionals as more important for people in the workplace than the employees with diabetes did. |
|  | Dures 2014^36^ | Physicians, physiotherapists, nurses, occupational therapists  Arthritis  UK | To explore rheumatology clinicians’ experiences of a variety of brief training courses in skills to support self-management  Interviews | Identifies three overarching themes in rheumatology clinicians’ experiences of training: 1) Putting theory into practice: participants thought training should be brief, have an applied focus, and balance provision of theory with time to practice skills; 2) Challenging professional identity: training had led some professionals to recognise that working with people in more holistic (rather than more reductionist or medically focused) ways, and moving to more collaborative approaches that attended to patients’ self-efficacy could require shifts in their own (and colleagues’ and managers’) sense of their professional roles and identity. These challenges to role and identity could make it hard to implement the skills and approaches promoted in training; 3) Enhanced practice: although the extent to which they reported making changes after training varied, all participants perceived their consultations to be enhanced as they made some shift from a focus on the clinician’s to the patient’s agenda, talking less and listening more and, at least in some circumstances promoting more patient-led problem solving. Participants preferred condition-specific training and examples, thought it would be beneficial for whole clinical teams to train together, and noted the positive impact of clinical supervision to help embed skills in practice and develop more advanced techniques over time |
|  | Eldh 2006^37^ | Nurses  Chronic heart failure; nurse-led clinics  Sweden | To explore the phenomena of patient participation and non-participation  Observations of clinic visits, interviews (nurses and patients) | Identifies differences between nurses’ and patients’ perspectives on both participation and non-participation. Suggests that these differences can have problematic implications, particularly because there was no evidence of dialogue between patients and nurses about what patients expected or how they wanted to participate. Reports that patients associated participation with being responsible and accepting responsibility for aspects of their care, and non-participation with an unequal relationship in which they were not listened to and were controlled by the nurses. Nurses in contrast associated participation with giving patients information based on individual needs and with patients feeling secure to follow recommendations. Nurses saw non-participation as patients not accepting the information or advice that they provided, and not being willing to go along with them. Reports researcher observations that the nurses dominated patients during clinic visits. Although they prompted patients to ask questions and gave additional information in response, the interactions and information given were rather standardised, and patients’ values were largely neglected. |
|  | Elliott 2010^38^ | Advanced nurse practitioners  Various long-term conditions  Ireland | To explore how nurses working in chronic and acute care outpatient contexts respond to decision-making concerns in clinical practice  Interviews | Highlights clinical judgement practices that advanced practitioners find useful in decision-making involving person-to-person interaction. Introduces ‘mutual intacting’ as an overarching term for a set of strategies that practitioners used to facilitate an effective co-construction of health problems and to reach treatment decisions acceptable to patients. The set includes ‘intacting a therapeutic relationship’, ‘situated patterning’ (to integrate a patient’s construct of the problem as it related to their lifeworld with the practitioner’s background knowledge of the practice context) and ‘intacting therapy’. Reports that participating nurses moderated patient treatments whilst striving to maintain a therapeutic relationship and, conversely, considered how the relationship affected patient treatment. Illustrates how effective clinical judgement involving person-to-person interaction can incorporate both scientific medical knowledge and practical wisdom in the sphere of human interaction. |
|  | Entwistle 2013^39^ | Various health professionals with known interests in ‘co-producing health’  Various long-term conditions  UK | To look critically at ideas about collaborative approaches to care and consider how well they reflect the complexities of practice and what is good about collaborative approaches in practice  Knowledge exchange discussions | Suggests that while current ideas about ‘patient activation’ and summary characterisations (models) of collaborative approaches to health care offer useful general pointers to forms of care that respect patients’ autonomy and promote effectiveness, efficiency and sustainability in health services, they have a number of limitations. These limitations include: a tendency to emphasise equality of patients’ and health professionals’ knowledge and contributions to care, while neglecting both the (variably) significant differences between patients and health professionals in these domains and the importance of equality in senses associated with mutual humanity and respect; a strong focus on explicit goal-setting by patients, without addressing the tensions this can raise or the valued aspects of care it can obscure; and the promotion of an idea of patient activation that incorporates strong cognitive, individualistic and biomedically normative assumptions and neglects the material and social realities of many people’s lives. Proposes shifts in thinking about these to reflect ideas relational theories about autonomy and capabilities approaches to thinking about quality of life. |
|  | Ferrante 2009^40^ | Family physicians  Obesity  USA | To assess family physicians’ practices and attitudes regarding care of extremely obese patients and factors associated with them  Questionnaires | Reports that a high proportion of family physicians, and particularly younger physicians with less experience of obesity, endorse statements that characterise working with extremely obese patients as difficult, and in part because of patients’ attitudes. For example, many physicians agreed or strongly agreed that: dealing with obesity and weight loss is frustrating (66%), treatment for obesity is often ineffective (51%), and they were pessimistic that patients could be successful in losing weight (34%). Many physicians reported frequently or almost always encountering the following challenges when discussing weight loss with obese patients: patients lacked discipline to lose weight (78%), patients want an easy way out (71%), patients do not have time to exercise (62%), patients have psychological problems (57%), patients deny having poor eating habits (54%), patient cannot exercise due to their weight (54%), patients are not motivated to lose weight (52%). Recommends educating physicians about bariatric surgery and community resources for extremely obese people but does not consider alternative ways of supporting people who are obese. |
|  | Ford 2006^41^ | GPs  Various long-term conditions  England | To examine skill differences between doctors who vary in how well they meet patients’ preferences for involvement in  Cross-sectional study of consulting behaviours and patients’ experiences | Observes that decisions about self-care and lifestyle are frequent in general practice, and judges most of these to have been doctor-led. Reports that some doctors had a relatively high proportion of patients report perceptions of involvement that matched their preferences, and shows that these doctors had a broader range of scores on a communication style coding scheme. Suggests that flexibility of consulting style is associated with (facilitates) more ‘congruence’ with patient preference for involvement than ‘rigidity’ in consultation approach. |
|  | Fox 2010^42^ | Dietitians, nurses, medical doctors, social workers, psychologists  Diabetes  Canada | To investigate how health professionals responded to evidence about intensive management of diabetes in the context of their day-to-day practice  Interviews | Reports a consensus among participants that there is sound scientific evidence that ‘tight’ blood glucose control reduces or delays the onset of diabetes complications, but also that diabetes management must be individualised to meet each patient’s circumstances, need and preference. Illustrates a range of responses to the tensions this can generate, including: a view that the research evidence obligated them to be ‘a bit more pushy’ about glycaemic control; a differentiation between theoretical ideals and what was achievable in practice – especially with commitments to be person-oriented and not just diabetes oriented, and an inclination to inform patients about the benefits of intensive management and leaving them to make decisions. Suggests that participants did not adopt either evidence based medicine or patient focused care at the expense of the other and surmises “that health professionals function within a sea of multiple discourses that both shape and are shaped by their experiences”. |
|  | Furler 2011^43^ | GPs, diabetes nurse educators  Diabetes  Australia | To explore views about starting insulin in general practice  Interviews | Reports that health professionals held different views of the main aim of primary care in relation to diabetes, and illustrates how these views had practical implications for the initiation of insulin. Health professionals who saw the main aim as to control blood glucose and tended to see initiating insulin as a simple protocolised process were more inclined to initiate insulin ‘early’. In contrast, health professionals who saw the main aim as nurturing an ongoing relationship with the patient in order to manage diabetes and other conditions tended to think that initiating insulin added complexity to an already overwhelming clinical picture. |
|  | Gambling 2010^44^ | Telecare providers and diabetes specialist nurse  Diabetes (type 2)  UK | To explore how patient-centred care is realised within the ‘Proactive, Call Centre Treatment Support’ intervention  Interviews (telephone) | Identifies four stages that telecare providers described working through with each patient: 1) Building a picture (scene setting) of the patient’s knowledge and perceptions of their diabetes and setting the ground rules; 2) Building a picture (person, condition and management) and understanding the patient in his/her unique psycho-social context; 3) Sharing and finding common ground; and 4) Implementing a sustainable plan (involving patients in choices to the degree they wish). Recognises that rapport and relationships need to be built up over time and comments that adoption of a patient centred approach ‘requires care providers to be flexible and recognise that patients vary in their knowledge, skills and illness trajectory’. Neglects to discuss the challenges of reaching shared understanding and involving patients in choices if health professionals are inflexibly committed to the pursuit of biomedical targets. |
|  | Garrett 2003^45^ | Pharmacists, diabetes educator and managers  Diabetes  USA | To ascertain views of what contributed to the success of a self-management program  Focus groups and interviews (professionals and patients) | Views program success in terms of improvement in glycosylated haemoglobin (HbA_1c_) concentrations, increased patient satisfaction with pharmacy services, and decreased costs of medical care for patients with diabetes. Reports several factors that study participants identified as contributing to the success of the program, noting that all emphasised that waived co-payments for diabetes medication and related supplies had incentivised patients to participate. Reports that patients valued participation because it brought an opportunity to establish ongoing relationships with caring and knowledgeable health care professionals, and helped them feel more in control of their lives. Providers saw patients’ success in the program as dependent on their viewing their health as a priority and on health professionals being willing to take time. |
|  | Gillibrand 2004^46^ | Practice nurses  Diabetes  England | To explore perceptions of care in the context of national guidelines and strategies  Interviews | Reports that the participants all identified education as the most important aspect of diabetes care. They discussed this at length and saw a need to improve access to resources and increase the range of educational materials. Notes that nurses recognised the emotional demands of diabetes and said they focused on underlying emotional problems when these appeared to affect self-management, although the authors state that it was not clear *how* they addressed emotional or psychological problems. |
|  | Goodrich 2011^47^ | Physicians and medical assistants  Various long-term conditions  USA | To develop and evaluate an online interface for referrals to an internet-mediated walking program  Interviews and review of program use | Provides an example of how web-based technologies can facilitate ongoing interaction and co-ordination between health professionals and patients. Reports that while providers were willing to refer patients to the program, they rarely monitored patients’ activity using it. Explains this in part by noting that the monitoring facility was not connected to the patients’ electronic medical records. |
|  | Graham 2012^48^ | Podiatrists  Rheumatoid arthritis  UK | To explore the nature and content of foot health education for people with rheumatoid arthritis  Focus group | Reports that participating podiatrists saw patient education for people with rheumatoid arthritis as important primarily for facilitating foot health self-management and enabling informed consent for foot health interventions. Also notes that the information podiatrists give is not necessarily specific to podiatry and that some of the education they engage in is a means of engaging the patient in their healthcare (more broadly).  Health education cannot be overly prescriptive in its content and that timing needs to take into account the patient’s defined need. This suggests that self-managing a long-term condition is understood to incorporate seeking advice relating to it, responding to patients’ questions is an important aspect of support for self-management. |
|  | Granger 2009^49^ | Cardiologists  Heart failure  USA | To explore patient and physician perspectives on adherence and experiences of exchanging information about heart failure management  Interviews | Illustrates how health professionals can lack insight into the ways that patients who understand *what* needs to be done in terms of condition and medication management can have difficulties in terms of *how to* achieve that. While patients saw their symptom exacerbations and problems with depression as making self-care more difficult, cardiologists thought it was patients’ lack of understanding of what needed to be done that contributed to a lack of self-care that in turn exacerbated symptoms. So health professionals tended to repeat their ‘what’ instructions to patients who already knew what, but needed help with ‘how’. Notes that the cardiologists also lacked awareness of the extent to which patients depended on family members to help with the how (e.g. checking salt content while doing the shopping, sorting medications when patients were too fatigued), and of patients’ experiences of the ‘work’ involved in following the regimen. |
|  | Greiner 2000^50^ | N/A  Various long-term conditions  USA | To develop an argument linking respect in patient-physician relations to the social determinants of health  Opinion piece | Starts from a recognition that physician-patient relationships can impact on the course and outcomes of chronic illness, and that successful partnerships require flexibility, continuity and mutual respect. Notes that ‘difficult patients’ and a view of medicine that tends to exclude social conditions are often identified as barriers to such partnerships. Observes that the difficult patient problem is usually considered in terms of differences between patients’ and providers’ viewpoints and values, with providers typically seeing the patient’s views and desires as counterproductive for restoring health. Notes that acceptance of this provider-driven conception of a difficult patient creates a number of problems, including making it difficult for those patients who most need to negotiate with healthcare providers to do so (as they are likely to generate more animosity). Suggests that the label of difficult should be removed from the lexicon of medical discourse about patients, and that clinicians need a deeper understanding of the social inequities that foster chronic disease. |
|  | Grimaldi 2012^51^ | N/A  Diabetes  France | To discuss how caregivers can best help people with diabetes to self-manage  Opinion piece | Argues that if health professionals really want to help support patients to self-manage, they need to demonstrate real emotional empathy. Suggests this involves: renouncing the desire to influence or manipulate; showing emotions; and relating to the patient as an individual person. Argues that empathy cannot be reduced to a few communication techniques (open-ended questions, reflection, synthesis etc.), and that in order to support patients, caregivers must get to know them and respect their freedom and self-determination. |
|  | Grimmer 2009^52^ | Primary care and community workers  Various long-term conditions  Australia | To explore experiences of leading chronic disease self-management programmes for vulnerable and disadvantaged people  Focus groups | Seeks to respond to suggestions that the Stanford model of chronic disease self-management, which involves a peer-led participative training programme for patients, is generally unsuitable and ineffective for vulnerable/disadvantaged people because it is insensitive to their diverse identities and circumstances. Reports the development of nine guiding principles for tailoring self-management programmes for vulnerable/disadvantaged people (including attention, for example, to learning styles, cultural sensitivities, group leadership requirements and language and literacy levels). Offers little justification for, or discussion about the implications of the guiding principles. |
|  | Hajos 2011^53^ | GPs, diabetologists, endocrinologists  Diabetes (type 2)  France, Germany, Italy, Netherlands, Spain, Sweden, UK, USA | To assess physicians’ understandings of patients’ perspectives on diabetes  Questionnaires (doctors and patients) | Presents findings from samples of patients and doctors to identical questions about their own or their patients’ thoughts about their diabetes. Reports that relative to patients’ responses, physicians on average tended to underestimate how seriously patients perceived diabetes to be, and to overestimate how distressing diabetes was to patients. Suggests these findings may signal that physicians lack confidence in or understanding of patients’ health awareness and coping abilities, which in turn might contribute to more paternalistic, rather than more collaborative professional-patient relationships. |
|  | Hale 2005^54^ | Physiotherapists  Stroke  New Zealand | To examine physiotherapists’ home interventions for people with stroke  Interviews | Reports that physiotherapists saw the primary aim of rehabilitation in the home environment as preparation for life after stroke, recognising that people who had been discharged from hospital after stroke were often fatigued, frustrated, depressed, and scared. The physiotherapists sought to promote optimal independent functioning by building patients’ confidence, self- responsibility, and problem-solving skills, while ensuring patient safety. They worked to patients’ priority goals and expressed frustration with ‘validated’ measures of their effectiveness, citing problems with ceiling effects, poor responsiveness, and a failure to assess what they were trying to achieve in terms of building people’s confidence and self-responsibility. |
|  | Harrison 2007^55^ | Nurses and policymakers  Heart failure  Canada | To assess the relevance and acceptability of a resource to support self-management  Questionnaires and focus groups (nurses) and interviews (policymakers) | Reports various elements of an evaluation of a resource that comprised; a Resources Guidebook (seven module educational approach for patients/families); an Education Map (teaching plan and documentation tool for practitioners); and a Resource Manual (structured abstracts of relevant studies and copies of key papers for practitioners). Reports that both nurses and policy makers were positive about the content of the resource and the way it helped the various people involved in a patient’s care to ‘talk the same language’ and work to similar standards. Most nurses endorsed statements to the effect that the resource streamlined the approach to support, made better use of limited teaching, was a reliable method to help learning, permitted selective teaching, and improved their satisfaction with practice. Policy makers also noted that it promoted consistency of information provision for patients, especially when staffing patterns were in flux. |
|  | Heldal 2009^56^ | Physicians and nurses  Blood conditions, including leukaemia, myeloma  Norway | To investigate how healthcare professionals relate to patients with different levels of knowledge and involvement in their disease and treatment  Interviews | Categorises participants’ descriptions of patients as ‘passive’ (not wanting or not being able to deal with information or involvement), ‘uncooperative’ (bringing in external information that they didn’t understand, refusing or quarrelling about recommended treatment), ‘withdrawn’ (informed but reluctant to take part) and ‘expert’ (informed about biomedical issues and cooperatively involved). Notes that health professionals’ perceptions of patients’ propensity to master medical knowledge affects how healthcare professionals involve them in decision making, and that professionals’ tend to see relationships with patients as asymmetric and stable. Expresses concern that professionals’ attitudes and behaviours may inhibit patients’ ability to involve themselves in their healthcare decisions. Suggests a need to raise professional awareness that and how biased views of patients influence sharing of decision making. |
|  | Holley 2007^57^ | N/a  Various chronic illness, rehabilitation  N/a | To provide practical guidance to rehabilitation nurses to prevent and relieve isolation among chronically ill people  Opinion piece | Acknowledged the significance of social contact for full and meaningful lives and the potential of loneliness to make the daunting tasks of managing health in chronic disease unbearable. Summarises evidence that functional limitations, personality changes, loss of social roles and transport difficulties can all contribute to social isolation in people who are chronically ill. Advocates roles for nurses in (1) assessing social isolation (including by attending to patients’ own perceptions of their situations), and (2) intervening, e.g. by (a) enhancing spirituality (being present and sharing the patient’s burden, and promoting feelings of control, self-esteem, meaning and purpose in life); (b) therapeutic use of self (providing caring, genuine high quality contacts); and (c) encouragement of peer counselling, support groups and internet use. |
|  | Holmstrom 2004^58^ | Physicians and nurses  Diabetes  Sweden | To investigate a communication skills intervention to help professionals understand their encounters with patients  Consultation recordings | Recognises [in introduction] that health services sometimes obstruct rather than support self-care and learning, in part because of the ethics of individualism (patient blaming), the privileging of expert over lay, and one-way information flows.  Categorises the educational models observed in recorded consultations as either prescriptive (based on medical facts), reflective (based on patients’ life experiences) or combined. Provides excerpts of conversations within consultations to illustrate these. Suggests supported reflections on recorded consultations could help encourage health professionals to adopt more patient-centred consultation patterns. |
|  | Hopp 2007^59^ | Nurses and nurse practitioners  Diabetes  USA | To obtain information on how a Monitoring and Messaging Device system is used for diabetes care  Qualitative interviews | Reports that providers had refined their decisions about which patients to offer and enrol in the system as they gained experience with it. Notes that the Monitoring and Messaging Device system added to staff workloads (at set up and with the ongoing need to monitor and respond to patient input and alerts). Identifies a number of clinical and non-clinical issues that informed nurses’ judgements about enrolling particular patients (e.g. level of diabetes control, need to adjust medications, health conditions that might make use of the system difficult, patient interest in the system and willingness to work on their diabetes control, availability of telecommunication services). Reports providers’ recognition that some patients answer monitoring questions selectively and/or monitor their results without changing other self-management practices (e.g. diet). Notes that nurses see some benefits and some issues with the system but stress it is just one tool they can use. |
|  | Hörnsten 2008^60^ | Nurses  Diabetes  Sweden | To describe nurses’ experiences of encounters with patients in diabetes care  Focus groups | Identifies four themes that describe conflicts (tensions) in nurses’ encounters with patients: 1) Implementing guidelines or being patient-centred; 2) Relying on medical knowledge or patient’s lived experience; 3) Being distanced and judging or close and emphatic; and 4) Being comfortable in an expert role or uncomfortably equal. Illustrates how professional training and professional and system norms tend to promote (or render nurses more comfortable with) conformity to guidelines, privileging of medical knowledge, judgemental attitudes (especially when talking about patients generally) and a powerful professional as expert role. |
|  | Horsburgh 2010^61^ | Health and social care workers trained in the Flinders program  Various long-term conditions primary / community care  New Zealand | To ascertain use of the Flinders Program of Chronic Condition Self-Management and identify barriers and enablers to its use  Questionnaires | Reports that despite extensive training, the health and social care staff made limited use of the various tools and processes associated with the Flinders Program. Of 152 respondents, 104 reported having completed six of fewer client assessments using the Flinders tools. Barriers to implementation were identified as: the time needed for structured appointments; funding; resistance from colleagues; lack of space; and insufficient on-going support. Suggests that without structured support for quality improvement initiatives and self-management programmes, health professionals’ ability to implement learned skills and complex interventions is limited. |
|  | Hughes 2009^62^ | GPs  Primary care  UK | To examine the experience of chronic fatigue syndrome or myalgic encephalomyelitis in primary care settings  Questionnaires (GPs and patients) | Recognises [in introduction] a range of ways that chronic fatigue impacts on patients’ lives, and notes that while many of the implications will be similar to those of people with other chronic illnesses, people with chronic fatigue syndrome or myalgic encephalitis have to cope with the additional burden of the stigma and controversy surrounding their condition. Reports that in an open ended question about which interventions they had found most helpful/unhelpful, patients frequently commented on GPs’ acknowledgement of the condition and its implications. The survey of GPs asked which of a range of interventions they offered to people with chronic fatigue or myalgic encephalitis, but acknowledgement of the condition and its implications was not among them. |
|  | Hunt 2001^63^ | Clinicians  Diabetes (type 2)  USA | To present an analytical framework for contrasting patient and provider goals, strategies and evaluation criteria  Case studies (anthropological approach) | Encourages clinicians to recognise different kind of goals and strategies so they can better understand how treatment behaviours are implemented and evaluated. Illustrates how providers and patients had similar ideas about the cause and course of type 2 diabetes but strikingly discrepant ideas about the long term enactment of self-care regimens. Providers' goals, strategies, and evaluations were grounded in a clinical context, in which the most salient factors are metabolic processes, clinical indicators, and prescribed behaviours. Patients appeared to know and be motivated to do what was needed to control the condition, but were rarely able to follow recommendations fully, in part to their impracticality - especially for people with limited economic and social power. Patients generated their goals, strategies and evaluations not within the circumscribed world of clinical meanings and measures, but in constant interaction with the unbounded world of everyday life action. They sought practical ways to apply specific behaviours and to identify links between behaviours and observed outcomes. Suggests ‘noncompliance’ is too simplistic a term when complex, long term treatment regimens are involved, and that providers' strategies to educate and motivate patients to be more cooperative seem misplaced. Proposes providers could attempt to identify and address differences between their clinical recommendations and assessments and what is, in fact, possible and practical from patients' perspectives, given the specific circumstances of their lives. |
|  | Hussain 2015^64^ | Primary care providers (doctors, nurse practitioners, physician assistant)  Hypertension  USA | To identify the functions that care managers could perform that primary care providers would find most valuable in hypertension treatment  Focus group discussions | Reports that while providers saw their own roles as indispensable for hypertension care, in disadvantaged populations particularly they experienced many practical challenges to providing the personalised treatment, education and teaching of self-management that they saw as essential for effective chronic disease control. Lists the functions that providers thought could be delivered by care managers within a PARTNER framework: Partner with patients, providers and the community; Arrange follow up care; Resolve barriers to adherence; Track treatment response and progress; Navigate the health system with patients; Educate patients and Engage them in self-management; Relay information between patients and/or providers.  Notes that ‘Providers described how collaborating with care managers who perform PARTNER functions would allow for increased patient centred care and more rapid blood pressure control’ but does not reflect critically on that belief. Reports that providers recognised e.g. that people might need help to translate recommendations about low sodium diets when faced with food labels or restaurant menus. [Appears strongly oriented to managing hypertension as a condition according to biomedical guidelines]. |
|  | Jallinoja 2007^65^ | Physicians and nurses  Obesity, high blood pressure, diabetes, dyslipidemia, primary care  Finland | To explore physicians’ and nurses’ views on patient and professional roles in the management of lifestyle-related diseases and their risk factors  Questionnaires | Reports that most physicians and nurses saw patients’ unwillingness to change their habits as more of a barrier to the treatment of lifestyle-related conditions than insufficient knowledge. Most also agreed that informing, motivating and supporting patients in lifestyle change were part of their tasks, but only about half thought they had sufficient skills in these. Identifies a dilemma in that the patient was recognised as central in lifestyle-related disease management but was also, if reluctant to change, a major potential barrier to treatment. Suggests that unrealistic and even idealistic expectations in respect of lifestyle change contribute to professionals’ rather negative judgements about patients’ willingness to change. Comments that these expectations can lead to attributions of total failure even if some lifestyle changes are achieved, and that this might lead some professionals to reject lifestyle counselling and rely on pharmacotherapy. |
|  | Jeffrey 2014^66^ | Diverse professionals  Various  Australia | To explore the implementation of health coaching training in practice  Questionnaires | Reports a low response rate to a survey sent to staff who had attended a specific kind of training course in health coaching. Although most respondents reported implementing the training to some extent in their practice, the majority also rated their confidence levels in using health coaching as low, and half saw their low confidence as a barrier to using health coaching with clients. The time required to use health coaching was also seen as a significant barrier. Suggests staff who identify as having low-level confidence may benefit from peer mentoring in the workplace. |
|  | Jeffrey 2012^67^ | Physical therapists  Nonspecific low back pain  UK | To understand how physical therapists’ personal experiences and feelings might influence their treatment decision making  Interviews | Reports that physiotherapists believe that non-specific low back pain has an underlying mechanical nature and that their role is to educate and empower patients to understand, to exercise and to self-manage their pain and functional problems. Notes that physiotherapists experienced tension when patients’ beliefs and attitudes were not consistent with their advice. Illustrates the challenges physiotherapists experience when (as they see it) patients don’t understand or accept that they need to take some responsibility for managing their back problem themselves. Identifies a sense of conflict that could arise when health professionals want to be working with patients but feel they are working against their wishes to get them to buy in to an empowerment and self-management agenda. Suggests that better communication training might help improve health professionals’ working relationships and encourage a more consistent approach to managing low back pain. |
|  | Johnson 2005^68^ | Diabetologists, vascular surgeons, GPs, nurses, orthotists, podiatrists, dietician  Diabetes  UK | To investigate how patients and professionals view the role of advice in diabetes foot care  Interviews (with vignette prompts) | Reports similarities between issues raised by patients and professionals, with differences in emphasis. Both recognised the importance of patients understanding diabetes and its implications from an early stage and both identified challenges in achieving this (including, for health professionals, organisational barriers and patient non-attendance at appointments). Patients noted that positive relationships with health professionals could allow a shared understanding to develop over time (and encourage participation in self-care). Professionals sought to pass on the essence of evidence-based guidance on specific foot care practices in consultations, but (as some professionals recognised at least on some occasions) this could seem unreasonable or impossible for patients if it did not reflect their needs and circumstances. Both patients and professionals saw patients as having some responsibility to follow advice, but health professionals were also concerned with their own and their organisations’ responsibilities to maintain effective communication with patients. |
|  | Johnson 2006^69^ | Diabetologists, vascular surgeons, GPs, nurses, orthotists, podiatrists, dietician  Diabetes  UK | To identify and discuss views on use of footwear for people with diabetes related foot complications  Interviews (with vignette prompts) | Reports that health professionals had differing expectations and perspectives on the difficulties in fitting footwear for people with diabetes-related complications in their feet. Recognises that people have difficulty wearing shoes that meet guidelines, particularly when their feet are changing (even within a day, e.g. with swelling) and when the available therapeutic shoes may not meet all specific needs and have negative implications for people’s identities and exposure to risk of known social stigma and embarrassment. Suggests that shoe design and professional practice need to shift to better take patients’ requirements and values into account to promote optimal use of therapeutic footwear. |
|  | Johnson 2008 ^70^ | Home care nurses  Various long-term conditions  USA | To explore factors that shaped the integration of web-based support for home care patients into nursing practice  Focus groups | Reports experiences of nurses participating in a trial of web-based support technology. Recognizes that the integration of this technology into home care required people to do tasks differently and make accommodations in the home for devices and computers. Identifies a need to be mindful of the existing expectations and skills of both nurses and patients when attempting to introduce such technology. For example, not all home care nurses are amenable to using home care technology, and patients’ abilities to play the more active roles it requires may be limited by pain, medication use, sensory limitations and economic considerations, as well as personal values and opinions. |
|  | Johnston 2011^71^ | Physicians, nurses, nurse practitioners, educators, managers  Various long-term conditions  Canada | To investigate health professionals’ perspectives of existing self-management support resources  Interviews | Reports that most participants requested a definition of self-management support before sharing information about programs they offered, and that while most programs offered patient education, they often lacked other elements of self-management as the project defined it (including action planning, problem solving, skill-building and longer term follow-up and support). Notes that participants reported the limited ability of existing programs and systems to address the needs of people with co-morbidities, and recognised that financial and transportation issues, as well as educational/literacy and cultural concerns prevented some people from participating in programs. Also notes awareness that the burden of self-management is greater for people on a low income and for new immigrants. |
|  | Jones 2000^72^ | Asthma nurses, GPs  Asthma  Wales | To explore the views held by GPs, practice nurses and patients about guided self-management plans in asthma care  Focus groups | Reports that neither health professionals nor patients were enthusiastic about guided self-management plans, although for different reasons. Highlights a gulf between professionals' concept of the ‘responsible asthma patient’ and patients' views. Most patients thought the plans were largely irrelevant to them. Most nurses thought patient education and ongoing monitoring were best achieved by patients attending nurse led asthma clinics. All claimed to give some kind of written self-management plan (typically ‘just a few pointers’ or ‘two or three instructions’) but only to patients they deemed to have accepted and understood their condition and be using drugs correctly. GPs were also unenthusiastic about standardised plans, sometimes disparaging their patients' capacity to take on information and self-management, and stressing the need for continuing education and dialogue. Suggests ‘most patients were managed by monitoring or policing’. |
|  | Jones 2013^73^ | GPs, practice nurses and a healthcare assistant  High blood pressure  England | To explore the views of primary health care professionals participating in a trial of patient self-monitoring with self-titration of antihypertensive medication  Interviews | Highlights a diversity in professional responses to the kind of extension to patients’ self-monitoring and associated self-adjustment of medication that has been made possible by developments in the portability, user interface and availability of monitoring technologies (in this case for hypertension). Reports that health professionals vary (1) in the advice and help they give to people about buying and using condition-monitoring devices that do not need a prescription and (2) in whether and how they integrate patients’ readings into their own record keeping and patient assessment/treatment adjustment (some ‘adjust’ for home readings, but in inconsistent ways). Also notes that health professionals were surprised how well some people whom they would not have thought suitable for self-monitoring got on when they were recruited into the trial, allocated to the self-monitoring arm and given training. Reports that health professionals were more enthusiastic about self-monitoring than about self-adjusted dose titration. They expressed concern about the need for patient training for medication adjustment, and about whether and how patients would choose not to increase their medication when their blood pressure readings were borderline and/or rose for a short time*.* |
|  | Jowsey 2009^74^ | Nurses, GPs, specialist physicians, allied professionals  Multimorbidity  Australia | To explore patients, carers’ and professionals’ views of the problems faced by people with multiple conditions  Interviews and focus groups | Identifies three ways that health professionals, as well as patients, report that co-morbidity can diminish patients’ abilities to manage: 1) by limiting ability to act on risk factors; 2) by making it harder to identify signs and symptoms of an exacerbation of an index condition; and 3) by interfering with capacity to manage medications and adhere to medication regimens.  Briefly notes that health professionals mentioned that they sometimes question patients’ honesty and accuracy of recall in relation to issues of compliance with medication regimes. Also mentions that if people with multiple conditions are more likely either to withdraw from, or require longer time in, rehabilitation programs for particular conditions, providers may be reluctant to refer them to these programs because of cost considerations. |
|  | Junius 2010^75^ | GPs, researchers  Multimorbidity  7 European countries | To advance a consultation approach based on shared priority setting  Workshop | Summarises the findings of a study previously published in German that highlights similarities and differences between what patients and health professionals consider important. Notes that health professionals rated as unimportant problems for which they had no solutions and problems that they saw as being someone else’s business to deal with. Emphasises the complexity of the challenge of sharing priority setting in general practice with older people with multiple morbidities. Questions the assumption that the reasons a patient offers for their encounters reflect the patient’s priorities. |
|  | Jutterström 2012^76^ | Diabetes specialist nurses  Diabetes  Sweden | To describe what specialist nurses perceive as conditions for ‘good care’ in type 2 diabetes  Focus groups | Reports discrepancies between the features of care that nurses considered be ‘ideal’ (counselling to empower patients; taking a comprehensive view of patients; nurse led; and built on quality) and the ‘real conditions’ of the care they described (counselling to govern patients; taking a biomedical view; doctor led; and standardised and/or delivered by staff who lacked skills to work effectively with non-compliant patients, so effectively unequal and poor in quality). Highlights the complexity of delivering the ideals of diabetes care, noting issues with conflicting paradigms and power relations that are perhaps particularly acute when patients are not complying with recommended self-management approaches. |
|  | Kendall 2007^77^ | N/A (review)  Various long-term conditions  [England and Australia] | To critically appraise the principles and use of the Chronic Disease Self-management Programme in national self-care policy  Opinion piece | Considers national policies relating to the adoption of the (self-management model associated with the) Chronic Disease Self-management Programme in the light of pre-existing models of self-management - including those of compliance with professional advice and of self-help based on mutual lay support and experiential learning. Critiques the tendency of the Programme’s focus on self-efficacy to attribute too much importance to individual level behaviour change and to cultivate a deficit view of people while neglecting their social contexts and lived experiences (including experiences of making sense of and representing their illness to themselves and others). |
|  | Kennedy 2002^78^ | Specialists and GPs  Ulcerative colitis  UK | To seek health professionals’ and patients ‘opinions on a guidebook designed to support self-care and mediate doctor-patient interaction  Interviews | Reports various reasons why both health professionals and patients were appreciative of the guidebook, but also notes that health professionals’ confidence in the guidebook as useful did not translate into their integrating it in to practice. Health professionals recognised that provision of a guidebook that patients could read and use in their own time to address their complex information needs was useful, especially because it could reduce the pressure on time in consultations. They also saw it as a useful tool to help patients take on a greater role in managing their condition and to confirm correct self-management actions. However, health professionals generally did not record patients’ test results in their guidebooks (some patients wanted this, but not all took their guidebooks to consultations). Suggests that some doctors seemed in some senses keen to retain their usual model of care. |
|  | Kennedy 2005^79^ | Consultant gastroenterologists  Ulcerative colitis or Crohn’s disease  UK | To investigate patient-centred consultations as an approach to improve patients’ ability to self-manage chronic illness  Mixed methods, including interviews within an RCT | Reports that specialists were willing to be trained in patient-centred communication as part of an intervention to establish guided self-management in patients with chronic inflammatory bowel disease, and that such training could be effective. The RCT evaluation showed the training led to a reduction in numbers of outpatient appointments and improvement in patients’ self-perceived coping abilities (enablement score). The interviews showed that both patients and consultants found the approach improved the clarity of communication and gave patients more confidence and resources to self-manage. Reports, however, that there were variations in the degrees to which consultants accepted patients’ opinions in particular domains, and that the development of mutually acceptable self-management plans was easier for patients with a history of successful treatment of relapses with standard drugs than for patients with more complicated and less predictable disease. |
|  | Kennedy 2007^80^ | N/A  Various long-term conditions  UK | To argue that effective self-care requires fundamental changes in professional attitudes and the way health care is delivered  Opinion piece | Challenges the assumption that training is all that is required to increase professional support for patient self-care. Acknowledges that training can equip professionals with useful strategies (e.g. for motivational interview­ing) but argues that there is also a need to understand and address the contexts in which health professionals work and the values they espouse. Notes that although professionals generally value self-care, there can be tensions between patient autonomy and pro­fessional responsibility for the delivery of evidence based care. These tensions are reflected in professionals’ expressed needs to monitor patients with long-term conditions and to ensure professional input into lay led courses. Notes that professionals sometimes put boundaries on patient participation and do not engage with aspects of self-care outside what they see as their professional perspective. Suggests that the potential of support for self-care to improve patients’ health and reduce their use of health services may be enhanced by intervention approaches that target health professionals and healthcare organisations as well as patients. |
|  | Kent 2010^81^ | Diabetes educators, behavioural science and mental health professionals  Diabetes  USA | To discuss ‘healthy coping’ in diabetes with a multidisciplinary expert panel  Workshop | Recognises that having diabetes brings psychological as well as physical challenges and discusses ‘healthy coping’ both as a response and as a self-care behaviour essential for effective diabetes management. Defines ‘healthy coping’ as ‘responding to a psychological and physical challenge by recruiting available resources to increase the probability of favourable outcomes in the future’. Notes that healthy coping is ‘a complex, qualitative behaviour that cannot be easily quantified’ and suggests indicators of healthy coping include: a person accepting that he or she has diabetes and integrating the reality of diabetes into his or her life; willingness to work with a diabetes educator; and willingness to set goals and make changes. Identifies numerous potential barriers to healthy coping and suggests a team approach is critical to address these. Recognises that ‘coping’ can mean different things in different cultures and suggests this should be reflected in treatment planning. Does not seem to question the prioritisation of metabolic control or the tying of the idea of healthy coping to particular behavioural norms. |
|  | Kirby 2012^82^ | Hospital and community clinicians involved in treating chronic disease  Various long-term conditions  Australia | To identify clinician, patient and service factors involved in uptake of chronic disease self-management services  Interviews (clinicians and patients) | Focuses on a particular hospital with a policy to refer people admitted with (exacerbations of) chronic disease to a self-management service and GP services within the same region. Notes that all the chronic disease self-management services were outside the hospital. Identifies various practical barriers to the making and use of referrals, including administrative burden, lack of resources for staff to follow up with patients and encourage them to act on referrals, and waiting lists for self-management services. Reports that although all clinicians supported the idea that readmissions could be reduced if more patients were self-managing, clinicians’ sense of their particular roles could limit their own involvement in support for self-management (e.g. hospital clinicians saw their role as to stabilise and manage patients to a point at which they can be discharged from hospital). Notes that while hospital clinicians and GPs thought some patients were resistant to self-management, the self-management service clinicians reported finding ways of winning trust, building rapport and so eventually negotiating some behaviour change. |
|  | Kosmala-Anderson 2010^83^ | Clinicians  Diabetes, COPD, musculoskeletal pain and depression  UK | To explore what factors facilitate and impede clinicians’ engagement in self-management support for long-term conditions  Questionnaires | Reports (from a cross-sectional survey) that (of the factors considered), feelings of competence, autonomy and internalised regulation to support self-management were the strongest predictors of clinicians providing support for self-management. Reports (from before and after surveys conducted among clinicians who attended a training programme) that attendance at a self-management training programme had a significant, positive impact on clinicians’ engagement in clinical self-management and patient centred-ness, as well as their overall confidence to support self-management. Recommends that organisations develop cultures that value self-management, offer training to clinicians to enhance their sense of competence to effectively deliver self-management, and support clinicians in finding their own way of supporting self-management. |
|  | Kremer 2004^84^ | Physicians providing HIV care  HIV  USA | To examine physicians’ reactions to people’s decisions to decline antiretroviral therapy against medical advice, and to examine how physicians conceptualise compliance  Interviews | Reports that people living with HIV who refused antiretroviral therapy sought to preserve quality of life, held critical attitudes toward allopathic medicine and feared anticipated side effects. Some also articulated preferences for alternative medicine or expressed moral objections. Characterises some physicians’ responses to patients’ decisions to refuse antiretroviral therapy as coercive and not consistent with the patients’ perspectives. Reports gender differences in providers’ conceptualisations of compliance. Female providers tended to view compliance in terms of collaboration, whereas male physicians tended to view compliance more in terms of patients’ capacity to adhere to the prescribed treatments. Suggests a need to focus on collaboration between physician and patient and better consideration of patients’ worldviews and healthcare beliefs. |
|  | Lake 2010^85^ | Health professionals working in diverse settings including community, acute and emergency  Various long-term conditions  Australia | To elicit health professionals’ views on their use of self-management techniques in their work place and to consider the influence of formal self-management training on clinicians’ practice  Interviews | Reports that professionals in diverse settings implement some form of support for self-management, often despite lacking formal training. Describes an ‘informed eclecticism’ in professional approaches, and notes that even the professionals who had received formal training in self-management models reported making selective use of particular elements rather than delivering a complete package. Recognises that health professionals can draw on different strategies for supporting self-management in different situations. Identifies five main elements: 1) collaborative care (emphasising partnership in therapeutic relationships; 2) encouragement of patients’ taking self-responsibility; 3) attention to the particularities of individuals’ situations and circumstances; 4) the use of structured support materials; and 5) linkage with community agencies and across professions/institutions. Highlights professional concern about the appropriateness of some self-monitoring devices. Identifies a conflict between health professionals’ support for the principles of patient self-responsibility and their feelings of professional responsibility for healthcare delivery in situations when they are unsure how well patients might manage complex medication regimens. |
|  | Langer 2014^86^ | GPs, practice nurses, health care assistants, administrative staff  COPD  UK | To examine patients’ experiences of a brief intervention by Liaison Health Workers and staff views of how this intervention was incorporated into primary care  Interviews | Explains that Liaison Health Workers (LHWs) offered up to four sessions with patients, usually in their own homes, to address psychological and social needs by a variety of means. Reports that patients were mostly extremely appreciative of the interventions, attributing diverse kinds help to the LHWs, commenting on how they listened, were interested, responded to their particular needs and invested in them in ways that recognised and motivated their own capacity for improvement. Reports that practice staff were uniformly positive about the LHWs, valuing their holistic approach and potential to help people to ‘take the reins’ and do more for themselves. Observes that practice staff referred people to the LHWs and saw the LHWs as addressing people’s social and psychological needs in ways that they themselves could not within the constraints of short consultations. Interprets this to mean that staff saw the areas of care the LHWs provided as peripheral to normal GP and nurse practice. Notes that LHWs did not significantly affect other professionals’ roles or workloads. Concludes that LHWs were accommodated by – rather than integrated into – practices. |
|  | Langstrup 2008^87^ | GPs, nurses  Asthma  Denmark | To describe the use of an online asthma monitoring system in primary care  Observations and interviews | Illustrates how e-health interventions do not always work to support patients’ self-management as intended and highlights issues relevant to professional support for patient self-monitoring more generally. The intention was that people with asthma could enter daily readings via a web portal, that they and their GPs could monitor these, and that the system would provide daily status messages and standardised asthma management guidance based on the readings. The system was not well used and was eventually withdrawn. Some GPs saw the system as a tool for patients rather than a shared platform for doctors and patients. They did not want to monitor patients’ entries or to use the system’s decision support facilities. Some GPs asked nurses to connect patients to the system. In these cases, the daily status messages were sometimes used by nurses rather than patients (or patients used the system for the nurses) and nurses sometimes drew on other information to justify not following all the system-generated recommendations e.g. about medication dose adjustment. Reflects that by choosing not to be continuously connected to patients via the system, GPs could keep their routines intact and bracket out their interest and responsibility for patients outside the clinical setting. They could also grant patients the liberty of not having to continuously be connected with and responsible to a healthcare practice. Patients did not seem to gain agency in relation to their treatment by using the system. |
|  | Lemay 2012^88^ | Community health workers  Diabetes  USA | To explore how encounter forms can be used by community health workers to guide and collect data on interactions with patients  Completed forms, work logs, interviews | Reports that community health workers thought the structured encounter form could help trigger appropriate questions to ask people with diabetes. Self-management goals were recorded for 62% of the documented encounters. 26% of these goals related to making or keeping appointments. Both community health workers and their supervisors within primary healthcare teams thought the forms were helpful both to guide and to monitor support for diabetes self-management. Notes that the form is unlikely to be appropriate beyond the setting it was designed for (community health workers supporting people living with diabetes). Does not comment on the tendency of the form to lead to a focus on encouraging system-recommended behaviours. Does not mention the possibility that if workers’ performance is judged via the forms, they might be discouraged from responding flexibly to patients’ priorities. |
|  | Löfman 2003^89^ [FINNISH] | Nurses, physiotherapists  Rheumatoid arthritis  Finland | To investigate staff and patients’ views about self-determination and how it might be actualised in healthcare situations  Focus groups | Reports that staff thought population-wide education (more than information provision to patients) was needed to ensure service users knew about their options and were encouraged to exercise agency in their health care. Indicates as well that staff were aware that patients who exercised agency and self-determination in health care contexts were currently sometimes perceived as difficult and demanding, especially if their goals conflicted with professional treatment goals. Suggests a need for culture change in health care to facilitate greater patient self-determination. |
|  | Lundh 2006^90^ | Community nurses and general nurses with interests in lung disease  COPD  Sweden | To report nurses’ perceptions of caring for patients with COPD  Interviews | Reports that nurses felt frustrated, powerless and insecure in their encounters with people with moderate to severe COPD, saying, for example, that they had little to offer these people, or that they had failed as nurses if the people did not stop smoking. Identifies two main orientations to care: 1) task-oriented nurses concentrated on performing examinations carefully. They gave patients the best available information to which they considered them entitled, and did not follow up patients on their own initiative; 2) individual-oriented nurses began with the patients and their problems, took their concepts and values into account, tried to ensure they understood their disease and treatment, and followed them up according to their needs or to a planned structure. Particularly on an individual orientation, nurses worked to create conditions for patients’ commitment and participation by avoiding expressions that might arouse feelings of guilt, establishing a relationship, creating a sense of security and inspiring hope, and respecting patients’ decisions. The educational strategies of nurses working with an individual orientation were more two-way and responsive than the advice and instruction-giving associated with a task orientation. Suggests a task orientation is encouraged when technical procedures are highly valued, organisational conditions discourage spending time on getting to know and following up, and/or staff lack knowledge and experience. |
|  | Macdonald 2008^91^ | Practice nurses  Various long-term conditions  UK | To explore practice nurses’ involvement in facilitating self-management for long-term conditions  Interviews | Shows that practice nurses’ accounts of working with people with long-term conditions support a differentiation of what they do and experience with people in the early, middle and later stages of living with a condition. Reports that nurses were most confident and secure in their roles around the time of diagnosis when they saw a need to educate patients about their condition and its management. Subsequently, they could find it harder to motivate patients, sometimes getting stuck with patients who seemed not to comply or make progress, sometimes persisting with the repetition of didactic messages. Observes that practice nurses categorised patients as good or bad (often from the point of diagnosis, anticipating self-management behaviours) and that their categorisations guided the ways they worked with people. |
|  | MacNeela 2010^92^ | GPs  Chronic low back pain, primary care  Ireland | To examine how GPs applied professional knowledge of chronic low back pain, especially in relation to psychosocial care  Medical records review and interviews | Notes different emphases in what is said about patients in medical records and in interviews. Illuminates tensions GPs can experience when patients might be working at variance with them, particularly when work and legal situations gave patients an interest in demonstrating serious and ongoing pain. Observes that GPs had no difficulties giving researchers personalised accounts of patients’ struggles for normality, of unhappiness and depression, but that in practice they experienced doubt/uncertainty about patients’ accounts and motivations, and were not entirely comfortable with psychosocial therapeutic communication and counselling. Reflects that while the GPs had clinical interests in understanding patients’ motivations to help them make sense of patients’ presentations, the therapeutic relationships they formed were ‘in the mould of medical sovereignty, a regulatory, biomedical orientation’ as they sought to work as guides (encouraging and motivating patients to act to manage the pain), gatekeepers (ensuring medications, specialist services and disability benefits were not abused) and moral guardians (encouraging return to work and social roles). |
|  | Malone 2005^93^ | Various  Diabetes  Northern Ireland | To discuss a service plan to address psychological needs in diabetes  Workshop report | Reports that when shown the findings of a survey of psychological symptoms among people with diabetes, health professionals were concerned at the levels of psychological distress experienced. They were often surprised at the levels of anxiety, and some had previously believed that poor adherence resulted from patients not being anxious enough. Notes a clear consensus that the health professionals involved in diabetes care in this setting did not currently have the capacity to meet the psychological needs of their patient group. Suggests integrating a psychological service within diabetes care provision. |
|  | McCann 2004^94^ | Community mental health nurses  Schizophrenia  Australia | To examine how community mental health nurses promote self- determination with clients experiencing an early episode of schizophrenia  Interviews and observations | Suggests that the promotion of self-determination depends on nurses both educating clients about their illness and wellbeing, and actively fostering self-control (self-control cannot just be prescribed, it needs to be supported). Identifies several guiding principles of education oriented to support self-determination, including: the use of an open, participatory and equitable approach, rather than a nurse-centred or expert-led one; being responsive to clients’ variable readiness to receive information; and supporting the development of understanding to help clients cope with the illness and to recognise and manage situational stressors and potential signs of relapse. Considers how nurses can foster and enable clients’ participation in decision-making and taking control of their recovery, recognising that nurses may need to take a lead in the early phases of acute episodes and facilitate a transition, for example by seeking opinion, listening, working together and encouraging. Suggests the development of a reciprocal relationship, or alliance, between nurses and clients is implicit in advancing clients’ self-determination. |
|  | McDonald 2008^95^ | Practice nurses  Long-term conditions, primary care  England | To examine nurses’ knowledge and role in encouraging self-care, perceived barriers, and attitudes towards the ‘Expert Patients Programme’  Interviews | Recognises and explores the identity work that practice nurses undertake in a context in which they are expected to work to targets framed in terms of patients’ behaviours and biomedical markers. Highlights nurses’ dependence on patients in the construction of their professional identities. The nurses studied saw themselves as more expert than patients and above patients in a hierarchy. Thus patients who were compliant and grateful tended to bolster these nurses’ identities, while patients who did not comply tended to undermine these nurses’ identities. Notes that these nurses’ identity work was not consistent with the promotion of an agenda of patient empowerment. Also notes that there are some repertoires or aspects of nursing identity that put more emphasis on intimacy and partnership with patients, but that these were not particularly evident in this study. |
|  | McIntosh 2003^96^ | GPs  Low back pain  UK | To ascertain experiences and expectations of information in low back pain  Interviews (clinicians and patients) | Illustrates issues that can make fulfilment of patients’ information needs far from straightforward. Recognises that patients and GPs both vary in terms of what information they consider useful. Notes that (honest) communication of diagnostic uncertainty and a lack of medical explanation for experienced pain can leave patients uncertain and frustrated, and can also tend to medicalise a problem that medicine cannot deal with. Notes the challenges of informing patients that medical advice has changed, especially if their self-management has included following medical recommendations for practices now considered harmful. Draws attention to the importance of ‘tone’ in information resources. |
|  | McLane 2003^97^ | Surgeons, nursing care managers, cooperative care nurses  Head and neck cancer  USA | To describe the effect of cooperative care on the treatment of head and neck cancer by exploring the perspectives of patients, care partners and health professionals  Interviews | Recognises the extensive self-care demands that patients and their care partners face on discharge from hospital. Reports generally positive responses to a co-operative care model within a hospital that introduced patients and care-partners to self-care by making care partners responsible for the patient’s routine care within a home like setting in the hospital while providing education and professional support. Notes that while medical professionals recognised some benefits to the cooperative care approach, including that patients and care partners became more informed and confident and less anxious, and called staff less often after discharge from hospital, some staff were less aware or somewhat dismissive of the psychosocial benefits that patients and care partners experienced (e.g. relating to autonomy, dignity and the development of a sense of community with other patients and care partners). |
|  | Mirzaei 2013^98^ | Community and hospital based nurses, allied health and medical staff  Chronic heart disease, COPD, diabetes  Australia | To explore and describe patients’, family carers’ and health professionals views of the challenges people with complicated chronic illness face when interacting with health services  Focus groups | Reports that healthcare professionals recognised the concerns patients and family carers raised, including about poor communication/ information provision, insufficient facilitation of self-care and lack of patient/carer involvement in decision-making about condition management. Notes that they suggested needs to improve health literacy, patients’ focus on management of conditions, and co-ordination and accountability between health care providers. Notes that they identified some scope to enhance communication between health professionals and patients (e.g. to ask people about tolerance and compliance issues before prescribing a particular medicine) and recognised that a more holistic approach would be better than narrowly focused clinical encounters, but saw a shortage of healthcare practitioners as a barrier to this. |
|  | Moffat 2007^99^ | GPs, practice nurses  Asthma  Scotland | To explore why asthma guidelines and self-management plans are not widely adopted in general practice  Interviews | Reports that participants expressed mainly positive attitudes to guidelines and self-management plans, but did not always consider them suitable for use with/by individual patients (e.g. because of severe/difficult asthma management, non-compliance and/or non-medical issues, including poor socio-economic circumstances) and sometimes judged professional and organisational issues (e.g. lack of role clarity, poor teamwork) as impeding their use. Notes that the most popular approach for dealing with patient asthma control issues was asthma-related education, although some professionals reported using persuasion or negotiation, and some accepted they were unable to influence patient issues. Suggests that guidelines were seen to provide the ‘why’ of helping patients self-manage but not the ‘what’ or ‘how to’ for particular individual situations. |
|  | Mulder 2015^100^ | Practice nurses  Diabetes (type 2)  Netherlands | To determine whether and how nurses applied five key elements of self-management support in standard care.  Analysis of audio-recordings of consultations | Reports that: nurses consistently discussed health behaviours with patients, but generally ignored knowledge, beliefs and emotions in their assessments; nurses provided a lot of general information and advice based on biomedical assessments, but did not give much specific or personalised advice; when patients mentioned barriers to performing health behaviours, nurses confirmed these were problematic but did not suggest strategies for overcoming them; nurses routinely arranged next appointments for patients but rarely arranged follow up to review strategies for overcoming difficulties. Reflects that issues within each of the 5As (assess, advise, agree, assist arrange) impeded their being used in an integrated way. Concludes overall that there are structural gaps in support for self-management. |
|  | Nam 2011^101^ | N/A  Diabetes  N/A | To summarise knowledge about barriers to diabetes management from the perspectives of patients and clinicians  Literature review | Categorises identified barriers to diabetes management as either patient factors (adherence; attitudes and beliefs; knowledge; culture/ethnicity/language) or health care provider factors (beliefs, attitudes and knowledge; patient-provider interaction and communication; health care system). Notes that many health professionals recognise that they lack effective communication, counselling and shared decision-making skills, and suggests it may be difficult for clinicians to change their communication style. Highlights the challenges primary care clinicians face in devoting time to people’s behavioural, psychosocial and emotional issues when they have short consultation times and must complete multiple preventive activities and prescribing/referral tasks. |
|  | Nasmith 2004^102^ | Family physicians  Diabetes  Canada | To assess a pilot project based on a care delivery model that sought among other things to foster self-care  Focus groups, interviews, participant observation, document analysis | Reports that both patients and physicians appreciated the benefit of having access to services such as nutrition, nursing and foot counselling that were not available to them outside the project. Notes the high costs within the project of conducting individualised assessments and of developing personalised education plans for patients then re-evaluating and documenting progress. Highlights the particular logistical challenges of accommodating multi-ethnic diversity in educational programmes. Recognises that the addition of services does not guarantee the integration of services. |
|  | Nelson 2013^103^ | GPs  Psoriasis  UK | To compare and contrast the perspectives of people with psoriasis and of GPs on the challenges of managing psoriasis in primary care  Interviews | Highlights discrepancies between patients’ and GPs’ perspectives, noting in particular that some GPs were inclined to view psoriasis primarily as a skin complaint (rather than more systemically) and to assume that patients did not want more information about, and were coping well with, their psoriasis. Notes that there was little evidence in GPs’ accounts that they routinely sought to share management of psoriasis with patients, and that some GPs noted that systematic follow-up of people with psoriasis was not incentivised in the same way that it was for other long-term conditions. Identifies missed opportunities for GPs to review patients’ condition or to engage in discussions about lifestyle advice and about coping with the social and emotional challenges of living with psoriasis. |
|  | Newton 2011^104^ | Experienced primary care professionals with strategic roles in diabetes care  Diabetes (type 2)  UK | To establish how healthcare professionals perceive patient empowerment and how this understanding informs their day to day practice  Interviews | Indicates significant variability in the ways health professionals understand and use a concept of patient empowerment. Reports that all participants rejected paternalism, but they did so to different degrees. Identifies that the concept could be understood in terms of (or used to inform): the development of a new patient-professional ethos; the skilling of patients to manage their diabetes (and perhaps an associated balancing of patients’ quality of life concerns against clinical aims); and a means of using limited resources expediently. Reflects that patient-empowerment can be seen in more professional or more patient-oriented terms, or as a way of balancing these concerns, as well as a means of improving quality of care and furthering social justice issues. Suggests that although empowerment as a concept is closely related to self-management and support for self-management, empowerment is broader in scope. Notes that despite being asked to work towards a patient empowerment agenda, health professionals are often limited in their ability to do this (at least in some senses) because the system they work within uses biomedical targets to assess performance. |
|  | Norris 2014a^105^ | Therapists (physio- and occupational-)  Stroke  UK | To explore the experience of stroke therapists trained in and currently using a professionally facilitated self-management approach  Interviews | Develops a contrast between what the therapists somewhat retrospectively understood as their previously usual practice as ‘benign dictators’ (committed to help their patients, but most comfortable when in control  ) and the ‘reluctant democracy’ that they worked in following adoption of the self-management approach (challenging themselves to empower stroke survivors to take control of their own recovery, but having to confront a variety of internal and external challenges in the resulting transition of power). Illustrates the significance of service contexts and cultures (various features of community rehabilitation settings render them more facilitative of patient engagement than acute stroke wards). Notes a number of factors that create tensions as professionals try to transfer control away from themselves, including questions about the acceptability of risks to the patient, how to respond when patients cannot clearly articulate goals or say they don’t want to work on anything, when and how to stress longer term implications that patients might not understand, and how to work with patients who just want to be fixed. Identifies obstacles to partnerships working as falling into three broad categories: professional models, practices and expectations; institutional demands and time; and the perceived wishes of stroke survivors themselves. |
|  | Paterson 2001^106^ | N/A  Diabetes  Canada | To investigate self-care decision making in diabetes  Interviews and self-recorded audio ‘think alouds’ (people deemed expert in self-managing) | Identifies, from patients’ perspectives, several covert and subtle ways in which health professionals contradict their stated intentions to empower people with diabetes. Illustrates particularly how health professionals frequently discounted the experiential knowledge of diabetes over time, used the possibility of events such as nocturnal hypoglycaemia to frighten people off anything other than strictly following their advice, and did not provide the resources people needed to make informed decisions. Mentions that some patients recognise that health professionals want to be empowering, but that empowerment is hard to do in practice. |
|  | Peyrot 2008^107^ | Primary care physicians and endocrinologists  Diabetes  USA | To determine what factors affect physicians’ recommendations and patients’ use of insulin pens  Questionnaires | Reports associations between more physician-reported initiation and/or use of insulin pens among patients and physician-reported: greater involvement in clinical practice; early adoption of clinical innovations; less preference to delay initiating insulin until absolutely necessary; more preference for patients to mix their own insulin; stronger perceptions of pens as efficacious and facilitating self-care. Illustrates how physicians’ support for patients’ adoption of particular self-management technologies can be influenced by their broader personal therapeutic philosophies and practices, as well as their perceptions of the technologies more narrowly construed. |
|  | Peyrot 2008 ^108^ | Diabetes educators and primary care physicians  Diabetes  USA | To examine issues relating to patients’ access to diabetes self- management education  Focus groups (by telephone) | Reports that while physicians generally want to ensure patients receive self-management support, they did not always refer patients to available self-management education programs or services. Identifies various possible reasons for this, including: a belief that they could provide sufficient education themselves; concerns that the costs, locations or language of delivery of some services would render them inappropriate for some patients; concerns about the quality of the education provided; concerns (sometimes based on prior experiences) that the education programs would advise patients differently (and especially work to lower targets for glycaemic control) to how they would. |
|  | Peytremann-Brideaux 2012^109^ | Various health professionals involved in diabetes care  Diabetes  Switzerland | To explore opinions on current diabetes care and the development of a regional diabetes program  Focus groups | Reports that health professionals mentioned their responsibilities in raising patients’ awareness of self-management but spoke of challenges with staff shortages, difficulties in multidisciplinary/team work, time management and administrative burdens. They associated difficulties motivating patients with the burden of treatment and proposed more self-management education classes (including for diet and physical activity) should be offered as part of a regional diabetes program. Also notes that some health professionals linked difficulties following up some patients to those patients’ personalities. |
|  | Philips 2014^110^ | Hospital and community clinicians  Chronic disease  Australia | To investigate clinicians’ perspectives on self-management, and which formal self-management  support initiatives they use and why  Interviews and questionnaires | Summarises clinicians’ perspectives on support for self-management primarily in terms of the provision of a holistic approach to health care that encompassed: patient involvement; positive interactions (between patient and clinician, among patients, and among clinicians; and support from clinicians and community providers). Notes clinicians’ recognition that patients vary in their understandings and expectations of self-management, as well as their abilities to self-manage, and reports some concern about the pressures that demands for self-management place on people. Reports that this sample of clinicians identified 54 formal initiatives to support self-management, including paper based (e.g. education materials, records) and interaction based (e.g. courses, goal and care planning, community based groups) initiatives, and reported a range of reasons for making particular selections between them. |
|  | Pitt 2008^111^ | GPs  Osteoarthritis  Australia | To examine barriers to, and drivers of, referral of people with osteoarthritis to self-management programmes  Focus groups | Reports that some GPs lacked knowledge about what self-management programmes offer to people with osteoarthritis and/or thought osteoarthritis less conducive to self-management interventions than conditions such as diabetes or asthma. Some GPs questioned the clinical benefit of the programmes. Notes that GPs were less likely to refer if they felt confident in their own capability to manage and address the needs of people with osteoarthritis. Several GPs indicated they would refer patients to these programmes as an adjunct to GP care particularly when they experienced difficulty managing the patient themselves and identified a need for a multidisciplinary approach. |
|  | Pooley 2001^112^ | Various health professionals involved in diabetes care  Diabetes, primary care  UK | To explore issues perceived as central to effective diabetes management, focusing on patient-practitioner relationships and implications for patient empowerment and  self-management  Interviews (professionals and patients) | Identifies five main themes in the analysis of interviews with patients as well as health professionals: (1) the importance of sufficient time for consultations; (2) the significance of continuity of care through a named individual; (3) the need for patients to have opportunities to ask questions; (4) the extent to which patients feel that they are listened to by health professionals; and (5) the variability of individual experiences of living with diabetes. Implicitly reports diversity (and some health professionals’ awareness of differences) in the ways health professionals comment or agree on why and what kind of patient question-asking, professional listening and attention to individuality are important (e.g. some were more concerned with patient education and compliance than with developing management plans in concordance with patients). Notes that health professionals feel constrained in how responsive they can be by available time and resources. |
|  | Proot 2002^113^ | Various health and social care professionals  Stroke, rehabilitation  Netherlands | To pinpoint dimensions of autonomy identified by care providers and to examine what influences patient autonomy during rehabilitation  Interviews | Reports professionals’ views that stroke can greatly diminish patients’ autonomy and that rehabilitation increases patients’ autonomy via a learning process. Identifies three interlinked dimensions of autonomy: (1) self-determination (freedom of choice and making choices); (2) independence (being able to do things without bothering other – including planning, organising and taking responsibility for one’s own life); and (3) self-care (including managing the activities of daily living). Participants stressed teamwork, attentiveness and respect among the strategies they used to increase patients’ autonomy during rehabilitation. |
|  | Ratanawongsa 2012a^114^ | Primary care physicians and nurse practitioners  Diabetes (type 2)  USA | To explore primary care providers’ perceptions about barriers to initiating insulin among patients  Questionnaires | Confirms that health care providers sometimes do not initiate insulin for Type 2 diabetes although guidelines would recommend it. Reports patient refusal or resistance, and concerns that patients couldn’t manage the regime, as key reasons for not initiating insulin. Neglects to consider when health professionals might be right to ‘accept’ patients’ expressed reluctance and/or deem them unable to self-manage a potentially helpful but challenging treatment regime, or when and how they might need to try harder/do better to educate patients. |
|  | Ratanawongsa 2012b^115^ | Primary care physicians, resident physicians, nurse practitioners, or physician assistants  Diabetes  USA | To compare (in a three-arm trial) group medical visits, automated telephone support and usual care for people with poorly controlled diabetes  Questionnaires | (Notes that health professionals were informed which arm of the trial patients were enrolled in before they answered questions about those patients and their care). Reports that health professionals could identify at least one barrier to good diabetes care that had been overcome for 68% of people receiving automated telephone self-management and 60% of people attending group medical visits. Reports that health professionals considered patients in the two intervention groups of the trial more likely than those in the usual care arm to have engaged in health-related goal-setting and to have played an active role in caring for their diabetes. Suggests that automated telephone self-management support may have been more useful than the group medical visits for people with low literacy or limited English proficiency. |
|  | Richards 2006^116^ | Various health and social care professionals  Depression, primary care  England | To explore stakeholders’ views a collaborative model of care involving mental health workers proactively initiating supportive contact with patients with depression.  Interviews, focus groups (professionals and patients) | Reports that while both health professionals and patients thought regular support for people with depression was valuable, health professionals put more emphasis on information and techniques and patients on worker warmth and emotional involvement and support with pharmacological treatment. Notes the “fine balance to be struck between offering support to patients and disempowering them” and identifies several tensions. For example, accepting help for depression in particular requires patients to acknowledge that they can’t cope alone, which may compound feelings of low mood and a sense of loss of role function. Also, “While accepting that activation is a powerful way of recovering health, some patients found the structured techniques simplistic and potentially infantilising”. Stresses that contacts by health professionals that are intended to be supportive “must be handled in such a way as to minimise the potential for further patient disempowerment”. Suggests the quality (manner) of communication is crucial. |
|  | Ring 2011^117^ | N/A  Asthma  UK, Australia, USA, New Zealand, Singapore | To synthesise primary qualitative studies to gain a better understanding of what helps or hinders asthma action plan use  Literature review (meta-ethnography) | Finds variable support among health professionals for asthma action plans for use by patients. Suggests that the promotion and use of asthma plans is currently limited because health professionals and patients/carers have different explanatory models of asthma, its management and their respective roles in the management process. Patients/carers often perceive themselves as capable managers of their asthma, but health professionals do not always share this view, and sometimes see themselves as experts with authority to ‘allow’ patients (or not) to take responsibility for their condition. Argues that medically focused action plans that do not fit with patients’/carers’ views will continue to be under-utilised, and that a partnership based approaches to plan development is needed to recognise patients’/carers’ experiential knowledge and ensure the plan is suited to their needs. |
|  | Ritholz 2014^118, 119^ | Physicians  Diabetes (type 2)  USA | To explore perceptions of barriers and facilitators to diabetes self-care communications during medical appointments.  Interviews (including with patients) | Reports that both physicians and patients described some patients’ reluctance to discuss self-care behaviours or share difficulties with self-care during medical appointments, and attributed this reluctance to both individual psychosocial and interpersonal (patient-physician) factors, including fear of being judged, guilt and shame. Both also stressed the importance of clinicians’ non-judgemental acceptance and of efforts to build an atmosphere of trust to encourage open and honest communication. Notes that physicians did not mention depression as potentially contributing to patients’ reluctance and points out that physicians described a dearth of strategies to overcome patients’ reluctance to share self-care information. Also, while physicians tried to communicate positively and promote hope, their accounts of how they went about this seemed somewhat narrower than the range of patients’ suggestions about fostering hope. |
|  | Robinson 2008^120^ | Community nurses  COPD  Australia | To report on the provision of training and support for community nurses to adopt a mentoring role for people self-managing COPD in the community  Feedback discussions | Reports that nurses who were trained and supported to mentor patients in a partnership role while developing their self-management strategies experienced a transformation in their construction of COPD and in their practice. Recognises that nurses sometimes held pessimistic, fatalistic attitudes to COPD, saw patients more as their disease than as people, and found them difficult to manage because there seemed limited scope for improvement. Reports that consideration of the mentoring role helped the nurses to develop new insights into patients’ perspectives and to see scope to improve their quality of life with the disease. Also reports shifts in practice from didactic to collaborative approaches oriented to enhancing patients’ self-efficacy, and from a biomedical, task oriented approach to one that incorporated more holistic caring. |
|  | Rogers 2002^121^ | GPs  Low back pain  Australia | To explore GPs’ attitudes towards respect for patient autonomy  Semi-structured interviews | Reports that GPs varied but were also internally (individually) inconsistent in their attitudes to patients exercising choice and/or GPs exercising control across four decision contexts that raise ethical and practical dilemmas (narcotic analgesia for pain management, where GPs have legal obligations and there is a danger of addiction/abuse; use of X-rays where research evidence suggests they will not be useful; use of complementary therapies, which patients can do themselves and GPs do not always see it as within their role to advise about; and duration of medical certification for time off work). Notes that GPs were sometimes in a position of having to accept patients’ statements (e.g. about the pain they were experiencing and their capacity to work) even when they did not believe them. |
|  | Rogers 2005^122^ | Consultant gastroenterologists  Inflammatory bowel disease, secondary care  England | To illuminate the findings of an RCT of a self-management program for people with inflammatory bowel diseases  Interviews | Confirms and helps explain patients’ reports of: consultants being reluctant to open up consultations and engage fully in dialogue; self-management being interpreted narrowly as compliance with medical instructions; and negotiations about self-management focusing solely on medication regimens. Reports that most consultants believed they had positive relationships with patients and already worked according to the principles of the self-management intervention. However, their interviews revealed that they seemed wedded to a belief that patients needed – and consultants were there to give – instructions. They saw written plans as medical instructions to be adhered to, and cut off options for patients to negotiate these, more openly because e.g. they saw patients as having too complex conditions, being too inexperienced, lacking knowledge or wanting directive advice, but also because they did not perceive diet as a priority in medical management and tended ‘to acquiesce to patients’ freedom to make dietary decisions, even though it was clear that for many people, diet was viewed as an important aspect of managing and controlling their condition’. |
|  | Rohde 2012^123^ | Speech-language pathologists  Aphasia (after stroke)  Australia | To compare the rehabilitation goals of people with aphasia and their speech-language pathologists  Interviews | Reports that the goals of people with aphasia related (1) specifically to improving communication and (2) to returning to previously valued activities (work, hobbies, travel etc.). The speech and language pathologists had goals relating to communication that could be seen as matched with those of the patients (although the professionals targeted language functions at an impairment level as well as in everyday life), but they did not share the goals relating to people’s return to valued activities. Identifies three key reasons why these broader goals are not shared: 1) the person’s impaired communication; 2) the service-delivery approach (a medicalised environment; policies about length of treatment); and 3) a belief that clients’ goals were outside the speech-language pathologists’ scope of practice. Highlights a need to improve professional understanding of clients’ goals and suggests a need to re-examine some professional beliefs. |
|  | Ross 2010^124^ | Physicians and neurologists  Epilepsy  France, Germany, Italy, Norway, Spain, Sweden, United Kingdom | To assess satisfaction with epilepsy management and the level of concordance in treatment goals between people with epilepsy and their physicians  Questionnaires | Reports discordance between patients’ and physicians’ perspectives on epilepsy management. Patients reported high levels of side effects than physicians estimated, and patients generally placed more importance on the balance between seizure control and the limitation of adverse effects than physicians did. Physicians rated patients’ knowledge lower than the patients themselves. Significant proportions of patients wanted to be more educated about treatment options and more involved in discussions about management. Suggests a need to improve patient-physician interactions and support for self-management among people with epilepsy. |
|  | Roy 2011^125^ | Primary care nurses  Various long-term conditions  New Zealand | To explore experiences of the Flinders Program^TM^ of self-management within an evaluation study  Focus groups (nurses and patients) | Reports that nurses appreciated the client-centredness of the approach of the Flinders Program^TM^ and the way it helped them to learn more about their clients. However, they reflected that they had been motivated and ready for change when introduced to the program, and that in the context of daily practice, constrained by existing health care structures, they could not use the Program in its entirety with every client. They talked about making adaptations on a client by client basis to manage time challenges. |
|  | Rutter 2011^126^ | Nurse independent prescribers  Various long-term conditions  England | To ascertain nurse independent prescribers’ receptivity to medicine deregulation  Questionnaires | Reports that nurse independent prescribers were generally supportive of recent and proposed changes to non-prescription status for medicines for acute conditions, agreeing that this would allow patients quicker and easier access to these medicines. However, they were less supportive of proposals to shift medicines for long-term conditions, e.g. obesity, asthma, and hypertension) from prescription only status, in part because they thought patients would not be adequately monitored and patient safety could be compromised. |
|  | Rychetnik 2013^127^ | Surgical oncologists, dermatologists, melanoma unit physicians  Melanoma    Australia | To describe melanoma clinicians’ perspectives on the functions of follow-up, factors that inﬂuence follow-up intervals, and important considerations for extending intervals  Interviews | Reports that follow-up was conducted for early detection of recurrences or new primary melanomas, to manage patient anxiety, support patient self-care (by educating about self-monitoring), and as part of shared care. Clinical guidelines recommend certain follow up intervals, but clinicians also wanted to take account of each patient’s clinical risk proﬁle, level of anxiety, education requirements and capacity to engage in skin self-examination, and clinicians also varied in terms of how they preferred to manage any suspicious lesions. Reports that clinicians emphasised the importance of professional-patient relationships based on trust and good rapport to facilitate patient education and support patient self-care. Suggests that extended follow-up intervals for early stage melanoma are more likely to be adopted after the ﬁrst year when patients are less anxious and sufficiently prepared to conduct self-examination. |
|  | Scambler 2012^128^ | Front-line diabetes staff from a variety of specialities  Diabetes  UK | To explore health care professionals’ views about patient empowerment in the context of diabetes  Interviews | Reports that health professionals saw various benefits of empowerment, including for patients’ learning, control (of condition management and condition) and satisfaction, and for the conservation of health professionals’ time and health service resources. Perceived barriers to empowerment included practical issues such as the limited availability of structured educational programmes and individual appointment times for patients, and lower levels of commitment and skills among some members of health service teams (which could mean patients were given mixed messages). Geographic variations in service availability led some professionals to talk about ‘selective empowerment’ referring either to a focus on sub-groups of patients who might benefit, or to the use of a limited set of empowerment strategies rather than none at all. Recognises that biological variation could mean people with ‘good empowerment’ had poor glycaemic control and suggests problems can arise when health professionals see empowerment as a means of achieving biochemical targets and judge empowerment by achievement of such targets. |
|  | Schaevers 2012^129^ | Multi-disciplinary transplant team (doctors, nurses, allied health professionals)  Lung transplant  Belgium | To report on the implementation of a standardised education program to prepare lung transplant patients for successful discharge from hospital  Service development report | Argues that lung transplant should be regarded as a chronic condition, because the surgery, as well as the underlying condition, has a significant long term impact including on life roles, and patients have ongoing consultations and relatively frequent hospitalisations. Describes and reflects on a structured pre-discharge education programme that was integrated with patients’ electronic medical records to support ‘education tracking’. Reports that this seemed to lead to more uniform delivery of information to patients, was appreciated by health professionals, and resulted in most patients feeling confident enough to go home. Notes that the programme focused on knowledge rather than skills. Perhaps illustrates a tendency for some supportively intended interventions to focus primarily on medical management issues, and to be didactic and somewhat inflexible. |
|  | Schold 2013^130^ | Diabetes specialist nurses  Diabetes  Sweden | To investigate opinions of self-monitoring of blood glucose  Interviews (nurses and patients, by telephone) | Reports that both patients and nurses were in favour of patients self-monitoring their blood glucose on the basis that it allowed patients to self-modify their treatment. Nurses also saw it as a means by which patients could learn more about the management of their condition. Reports that nurses took patients’ age and the status of their condition into account In deciding whether or not to prescribe self-monitoring of blood glucose. Also reports that they relied on their own experience and knowledge of the patient, rather than clinical practice guidelines, to make this decision. |
|  | Seto 2010^131^ | Heart failure clinicians  Heart failure  Canada | To assess attitudes toward the use of mobile phone-based remote monitoring  Interviews (clinicians and patients) | Reports that using a mobile phone-based remote monitoring system was acceptable for both patients and clinicians, but only where the system was easy to use and offered clear benefits. Suggests that the system should promote positive communications between patients and clinicians, but increased clinical workload would need to be addressed. Clinicians expressed concerns about the lack of remuneration for telephone interactions and medico-legal implications, worrying they could be legally liable if they did not respond immediately and the patient’s health then worsened. Clinicians recommended that a viable solution would be a method for documenting their actions. |
|  | Seto 2012^132^ | Cardiologists and nurse practitioners  Heart failure  Canada | To provide insight into the effects of tele-monitoring on self-care and clinical management  Interviews | Reports that clinicians who had participated in a trial of a tele-monitoring system (in which patients sent daily weight, blood pressure and symptom readings by mobile phone, information was stored and made accessible to patients and clinicians, and alerts were triggered by signs of deterioration) recognised that the system could help people learn how their medication, diet and exercise impacted features of their condition, and that they were enthusiastic about their ability to engage with patients in a timely manner when the system alerted them to signs of deterioration. Suggests some clinicians may have been more confident about introducing an additional medication that requires careful monitoring because of the monitoring system. Notes that both the clinicians and patients had some concerns on using the tele-monitoring system long term. For clinicians these included operational funding for the system, potentially increased clinical workload, and the sharing of monitoring responsibilities to cover all hours. Reports that some patients did not want to be watched persistently over the long term. Highlights some characteristics of the system that were perhaps important for its success: (1) provision of immediate self-care feedback to patients, (2) provision of immediate clinical feedback as necessary (with options to set thresholds for alerts on an individual basis), (3) ease of use and portability, and (4) perceived benefits of the tele-monitoring system for patients’ continued adherence to medication and other self-care behaviours. |
|  | Sharma 2013^133^ | Clinician and lay tutors on self-management courses  COPD  UK | To evaluate the co-delivery styles of lay and clinician co-tutors  Observations (including using rating scales) interviews | Identifies benefits to the combination of clinician and lay tutors on self-management courses for patients, but notes that the co-tutors raised concerns about delivery and power dynamics, and saw a need for more training for lay people. Reports that observational rating scores for providing structure and engaging participants were higher than scores for supporting autonomous decision-making and involvement. Notes that the manual based nature of the courses limited the flexibility for tutors to modify their approaches, and may also have limited the ways and extent to which they shared knowledge and experience with participants. |
|  | Sheares 2007^134^ | Medical pulmonologists and allergists  Asthma, specialist care  USA | To characterise the attitudes, beliefs and self-reported practice behaviours regarding the use of written treatment plans for asthma  Interviews | Reports that physicians varied in terms of whether, why and with which patients they reported using written treatment plans (e.g. some required patients to understand the disease and medications before they would give written plans, while others used them particularly for patients who had difficulty understanding and repeating verbal instructions). The scope their plans gave patients to adjust medication were limited, and some plans instructed patients either to call the physician immediately about changes in symptoms or peak flow assessment or to make medication changes but then call the physician. Some physicians believed written plans improved self-management and clinical outcomes. Some physicians who did not use written treatment plans explained they did not trust patients with them, and some worried that patients might rely on their plans and not keep follow-up appointments. |
|  | Shortus 2013^135^ | GPs, allied health professionals and endocrinologists.  Diabetes, primary care  Australia | To investigate provider perspectives on the role of patient involvement in the context of ongoing provider-patient relationships during routine care  Interviews | Reports that health care providers described a conflict between their responsibilities to deliver evidence-based diabetes care and to respect patients’ rights to make decisions. All sought to do the right thing for their patients with diabetes, but some prioritised ‘treat to target’ while others prioritised a ‘personalised care’ approach. Those preferring to treat to target ‘managed’ patient involvement to limit scope for patients to distract from the pursuit of biomedical ideals. Those practicing ‘personalised care’ were more accepting of patients’ priorities. Suggests that questions about patients’ involvement in particular (single) treatment decisions offer only a limited view of what matters for quality. Suggests that quality care for people with chronic diseases depends on them being both well cared for and respected. |
|  | Simm 2011^136^ | Community matrons (senior nurses)  Various long-term conditions  UK | To evaluate the usefulness of a small initiative to train community matrons in solution-focused approaches  Interviews | Notes that matrons’ prior hopes for solution focused training included ‘to learn an approach which might help us to work with ‘fix me’ patents whom we can’t make medically better’. Reports that a year after the training, four of five participants had experienced shifts in the location of expertise in their matron-patient dynamics (the fifth suggested her personality led her to be more directive and assertive). Illustrates how those who had shifted now talked differently with patients, particularly that they noticed and drew out patients’ existing skills in living well despite their difficulties, and that they found ways of enabling patients to take some responsibility for resolving their own concerns. Reports that the matrons had found the new approach unusual – and sometimes difficult – at first, but noted that it could ultimately save time, improve patient wellbeing and improve their job satisfaction. |
|  | Singleton 2000^137^ | Nurses  Rehabilitation unit  USA | To explore nurses’ perspectives of encouraging clients to care for themselves  Focused ethnography: observations, interviews | Illustrates how nurses working in a task-oriented, hierarchical, medical model culture found ways of addressing clients’ bio-psycho-social-spiritual needs and encouraged clients to care for themselves. Identifies 5 categories of activity explicitly associated with such encouragement: 1) co-ordinating and involving others in carrying out the client’s plan of care; 2) talking and communicating with clients; 3) assessing clients; 4) teaching clients staff and families; and 5) reinforcing with clients. Observes that, through their actions, the nurses created legitimate ways to interact with clients and develop relationships with them. Suggests that it was through these relationships that they encouraged care of self, and identifies three ‘mediating themes’ that represent ways in which nurses work through the conundrum presented by the mismatch between their organisational environment and their broader plan: 1) taking time (including to understand clients’ experiences, build relationships); 2) engaging dialogue of presence (securing clients’ attention and involvement); and 3) ‘day trips towards restoration’ (sensitively re-orienting, encouraging and reinforcing but not rushing or pushing). |
|  | South 2010^138^ | Trainers and staff from organisations hosting self-care training.  Various, community  England | To examine the feasibility, relevance and acceptability of a community based self-care training initiative,  Interviews and focus groups | Reports that a range of organisations agreed or sought to host the ‘Self Care for People’ training course and that they cited a variety of reasons, but generally did not emphasise demand management in primary care. Some organisations chose to re-name the course to avoid the term self-care. The course was delivered in both workplace and community group settings, including in organisations supporting socially excluded groups. Flexibility in delivering the course was seen as important, and skilled facilitation was considered essential because participants frequently raised sensitive issues. |
|  | Steinmann 2011^139^ | Physicians (primary care, general internal medicine, cardiology, nephrology)  Hypertension  USA | To assess health care professionals’ knowledge, attitudes and practices regarding home blood pressure monitoring  Questionnaires | Reports that health professionals used home blood pressure monitoring for both diagnostic and therapeutic purposes. When asked about barriers to greater use, they endorsed a range of physician and patient factors. The most consistently endorsed reason for patients not using home blood pressure monitoring was that their healthcare provider had not asked them to. Reports that health professionals also agreed that use of home blood pressure monitoring was limited by patients’ concerns about instrument accuracy, physical difficulties in using the monitors, not wanting to be preoccupied with or worried by readings, and preferring health professionals to do the checking. |
|  | Steurer-Stey 2006^140^ | GPs and pulmonologists  Asthma  Switzerland | To investigate physicians’ knowledge and implementation of self-management  Questionnaires | Reports that most physicians agreed that patient education offered a range of benefits (including increased knowledge, improved quality of life, reduced emergency visits or hospitalisations and lower costs) and that it was useful in most people with asthma. Notes that fewer respondents regarded patient education as something they personally should be doing (there was a preference for this to be provided in a specialist centre). Reports that more physicians said they provided information than that they checked inhaler techniques, instructed patients how to take peak flow measurement at home, or provided action plans for situations in which peak flow or symptoms deteriorated. |
|  | Sunaert 2011^141^ | GPs  Diabetes, primary care  Belgium | To explore why GPs were (initially) reluctant to refer patients to a self-management education program  Interviews | Notes a number of reasons for GPs’ initial reluctance to refer people to a self-management education program, including concerns about whether the program respected their role in care, whether it would further fragment the care people received, and whether it would add value to existing care – and for which patients. Reports that several GPs had become more willing to refer patients when they had seen a patient who participated in the program and understood how it helped. Highlights a need to attend to questions about how educational programs intended to support self-management are integrated with formal health care provision. |
|  | Thille 2014^119^ | Dieticians, physicians and other family health practitioners  Diabetes  Canada | To illuminate variations in clinical approaches to self-management dialogue  Discourse analysis | Stresses a ‘norm’ that the goals of self-management should be patient-driven and exclusively behavioural. Uses a detailed consideration of the way a dietician and a physician communicate with a particular patient to illustrate how, in practice, they activate some concepts of self-management support but also shift the focus away from behavioural goals to biomedical outcome goals (including body weight). Shows how measurements that are “assumed to signal the ‘truth’” about a disease can come to dominate conversations between clinicians and patients and – especially when combined with efforts to stress patients’ responsibility – can put patients into a “failure position”. Suggests that when behaviour changes are seen as means to biomedical ends, they can undermine attempts to frame patients as experts and clinician-patient interactions as partnerships, and might also impede the development of self-efficacy. |
|  | Thille 2010^142^ | Family physicians  Chronic illness (multiple conditions)  Canada | To apply a critical discursive psychology methodology to analyse discourse about the care of people living with multiple illnesses  Interviews | Reports that physicians all constructed effective chronic illness management as being dependent on patient involvement, but that there were competing constructions of ‘effective chronic illness management’ and ‘patient involvement.’ Notes that most physicians were consistent in the story they told, but a few expressed elements of both stories, at times in seeming tension with each other. Uses critical fictions to highlight the two main constructions. One construction integrated individual patients’ responsibility for their health with primacy of ‘evidence,’ resulting in a conceptualisation consistent with paternalistic care. The other constructed effective care as involving active partnership of physician and patient, implying a need to foster the ability of both practitioners and patients to respond to complex challenges as they arose. Comments that the former pattern is inconsistent with visions of family medicine and chronic illness management, whereas the latter embodies it. Concludes that collaborative, patient-centred approaches to chronic illness care require discourses that can bridge evidence and patient centeredness. |
|  | Thomson 2008^143^ | Physiotherapists  Chronic pain, inpatient rehabilitation program  England | To describe and interpret interactions between therapists and participants in a three week intensive rehabilitation program for people with chronic pain  Ethnography: observations and interviews | Explains that the program focused on collaboratively set functional goals, and that goal attainment was the benchmark of the program’s success. Observes that therapists worked from patients’ initial general goal statements (e.g. I want to go back to work’) to develop exercise and action plans, ensured patients understood key chronic pain issues then focused on working collaboratively with patients to facilitate their achievement of their goals. Identifies three different positions that patients adopted (and held quite consistently) in their interactions with therapists: 1) resistant to the success of the programme 2) unable to synthesise insights gained on the programme for use in life outside the hospital or 3) indicative of use of the programme to change and improve their lives. Suggests resistance may have arisen in some patients when the program made some people realise they had more to gain from their current role than from confronting fears and making changes. Suggests inability to synthesise insights for change may have reflected interactions within the hospital that appeared collaborative but were in fact paternalistic (and that for some patients the status quo was not sufficiently unbearable or the risk of change was too high to support change at a particular time). Observes that successful interactions often involved patients expressing their thoughts and feelings openly, even if this contradicted those of their therapists, and that the therapists handled these negotiations in ways that ensured neither party exercised power over the other. Reflects that even the most able therapists could not create a collaborative framework with patients who were unwilling to grasp the opportunity that the program offered. Comments that the program might have positive outcomes for patients beyond goal-achievement (e.g. improved relationships with family and friends, more acceptance of their condition), but that while therapists were perhaps amenable to this idea, they were committed to using a Goal Attainment Scale. |
|  | Tisler 2006^144^ | Primary care physicians  High blood pressure  Hungary | To obtain views of primary care physicians about home blood pressure monitoring  Questionnaires | Reports that home blood pressure monitoring was very popular among respondents, with 90% physicians saying they recommended its use often or almost all the time and 75% saying the results were of considerable or extreme importance. The most frequently endorsed reasons for use were: white coat hypertension; assessing 24 hour drug effects; improving compliance; suspicion of hypotension; and resistant hypertension. The most frequently endorsed concerns were the use of non-validated devices and patient pre-occupation with blood pressure. Reports that 65% of respondents said they reviewed data from home blood pressure monitoring only to obtain a ‘general picture’ (rather than analysing the data to determine values and trends). |
|  | Upshur 2006^145^ | Family practice doctors, nurse practitioners, physician assistants  Chronic pain, primary care  USA | To assess satisfaction with training and current practice in management of chronic pain in community clinic settings.  Questionnaires | Reports that the majority of attending physicians and over 40% of residents rated their medical school education and residency training about chronic pain as insufficient. Mean ratings of satisfaction with treating patients with chronic pain were also low. Notes that the top four problems endorsed by providers as frequently or always limiting their ability to achieve optimal pain control were patient related (patient self-management problems, patient psychological factors, patient compliance with treatment recommendations and patient occupational factors). Provider and practice system problems such as difficulty co-ordinating or adding on chronic pain management with other chronic diseases, lack of evidence based guidelines and time/tracking for regular follow up, were all less frequently endorsed. |
|  | Urowitz 2012^146^ | Various medical, nursing and allied health staff  Diabetes  Canada | To evaluate the experience of patients and providers using an online diabetes management portal for patients  Questionnaires | Identifies some concerns from health professionals about the value of an online site that, among other things, provided a portal for patients to log diabetes-related health metrics that health professionals could monitor. Reports that providers thought patients (and especially newly diagnosed diabetics) could benefit from being able to view trends/graphs of key indicators, but also thought the portal was used mainly by patients who already monitored their condition, so did not think it would promote uptake of self-management. Also reports that providers expressed concern that patients might rely unduly on the portal to the detriment of professionally provided care, especially if they assumed (wrongly) that health professionals were constantly monitoring patients through the portal. |
|  | Van den Berg 2005^147^ | Paediatricians and GPs  Asthma  Netherlands | To get insight into physicians’ views on patients’ asthma management  Structured questionnaires | Reports that (comparing the findings from this survey to the findings from a survey of patients that used parallel questions) both paediatricians and GPs overestimate the proportion of people with asthma who consider their asthma to be completely controlled, and underestimate the extent to which they experience symptoms of asthma, activity limitation, and a need to take time off school or work. |
|  | VanHooft 2015^148^ | Nurses  Chronic conditions  Netherlands | To reveal distinctive perspectives of nurses toward self-management support in  chronic care  Q-sort exercise with interviews | Identifies four clusters of nurses’ perspectives based on the ways they ranked their levels of agreement with 37 statements about self-management support (e.g. you should only support the patient if he asks for it; you must unconditionally accept the choice of the patient, even if this deviates from your perception of good care; you should always provide options for the patient) within a structured exercise. The four perspectives were labelled: Coach, Clinician, Gatekeeper and Educator. They reflected different understandings of patients’ and nurses’ roles, characterisations of nurse–patient relationships, and goals of self-management support. Coach type nurses focus on the patient’s daily life activities, whereas Clinician types aim to achieve adherence to treatment. The goal of self-management support from the Gatekeeper perspective is to reduce health care costs. Finally, Educator types focus on instructing patients in managing the illness. |
|  | Verlinde 2012^149^ | N/A  Various long-term conditions  N/A | To provide a systematic review of literature on the social gradient in doctor- patient communication  Literature review | Reports that studies consistently show a social gradient in features of doctor-patient communication that patients from lower social classes (measured by income, education or occupation) receive less socio-emotional talk, a more directive and a less participatory consulting style. (A less participatory consulting style is characterized by, for example: less involvement in treatment decisions; a higher percentage of biomedical talk and physicians’ question asking; lower patient control over communication; less diagnostic and treatment information and more physical examination). Considers how both doctors and patients influence these differences, but nonetheless suggests that patients’ social class and socio-economic status may affect practice of support for self-management. |
|  | Vetter-Smith 2012^150^ | Physicians, advanced practice nurses or family nurse practitioners  Diabetes  USA | To identify the roles nurse partners play in diabetes self-management support in rural primary care  Interviews and observations | Reports on a project that funded a ‘nurse partner’ post for support for diabetes self-management in five rural clinics in the USA. Identifies a range of ways in which nurse partners with relatively limited qualifications could make a useful contribution. Notes that the educational levels of the nurse partners, the ways established staff served as gatekeepers between them and patients, and the availability of time and space within clinics, influenced their ability to provide self-management support. Provides a list of tasks (labelled ‘roles’) that the nurse partners undertook and indicates how they could work to the benefit or detriment of self-management support (e.g. some clinical and administrative tasks provided opportunities for the nurse partners to build rapport, learn about patients, address questions and check understanding). Exemplifies a tendency for projects to promote self-management to be assessed in terms of biomedical outcomes (here blood glucose and lipids). |
|  | Visse 2010^151^ | Medical doctors, nurses, therapists and managers  COPD  Netherlands | To reflect on questions about professionals’ and patients’ roles and responsibilities in the context of an evaluation of a program that supports people struggling to accept or manage with COPD  Interviews | Argues and illustrates that the provision of (successful) support for self-management is complex and requires health professionals to engage in relational, narrative and communication work. Describes how staff on the program studied started with attention to patients’ life stories, tried to integrate considerations of mind and body, saw self-management as involving knowing what decisions to take in one’s own interest, and encouraged learning from fellow patients. Reflects that, rather than focusing on independence and control as some ideas about support for self-management might suggest, the program involved a new division of responsibilities, in which patients and professionals needed to develop new roles and relationships. Suggests that an understanding of relationality is key to effective support for self-management, and stresses the importance of values like equality, autonomy, and genuine involvement (which replace values of hierarchy and control). Suggests health professionals need ‘to develop skills like empathy, giving support and listening’. |
|  | Warren 2013^152^ | Eating disorder treatment providers  Eating disorders  USA | To examine the experiences of treatment providers with a personal history of eating pathology  Questionnaires | Highlights how health professionals’ own experiences of a chronic condition (or symptoms related to a chronic condition) can affect the ways in which they treat and relate to their patients with the same/similar condition. Reports that the large majority of participants (94%) believed that their own eating disorder history had a positive influence on their treatment of patients (e.g., increased empathy, greater understanding of the disorder, more positive personal outlook), while only 8% identified potential negative consequences (e.g. feeling personally triggered*,* over-identifying with patients). |
|  | Watts 2009^153^ | Nurse practitioners, pharmacists, medical doctors  Diabetes, heart disease, hypertension  USA | To describe the roles of nurse practitioners in disease-specific shared medical appointments  Case studies (data collection unclear) | Identifies several roles that nurse practitioners can play in shared medical appointments (group consultations), including bringing a ‘holistic perspective’ to information provision and self-management education. Suggests that nurse practitioners’ roles in self-management support are ‘highly dependent on communication and integration skills’. Nonetheless reflects a strong focus on professionally prioritised biomedical goal, and perhaps illustrates a limited shift to collaborative approaches (e.g. stresses the importance of information exchange rather than information provision, but does not mention any possibility of tension in the ‘collaborative development of self-care plans with each patient that reflect both treatment indications and patient preference’). |
|  | Wilkin 2006^154^ | Mental health practitioner  Mental health  England | To describe and illustrate an approach to therapy that integrates self-healing capacity into models of collaborative caring  Opinion piece | Offers observations and reflections on therapeutic processes oriented to recovery in mental health contexts that are useful for support for self-management. Highlights the ‘paradox of disabling caring’ in which expert-provided medication, therapy or hospital admission reinforce patients’ self-perceptions of not coping. Notes that clinicians tend to ‘trade reciprocally’ (offering clever therapies to those who play the role of a ‘not-knowing’ patient) rather than encourage patients ‘to find their own way out’. Suggests that even within target-led organisations ‘the unrivalled intimacy of the therapeutic relationship affords [clinicians] the opportunity to offer other possibilities to people’. Presents ‘an account of therapy delivered from outside the parameters of a health-illness model of caring’, identifying four main ways of ‘being’ that enable the clinician to engage: negative capability (a receptive pause in which the clinician ‘holds’ the patient well enough and tolerates their own not knowing while gradually coming to be guided by the patient’s signals); disinterestedness (setting aside the clinician’s own agenda to be able to hear the patient’s); imagination (allowing spontaneity and intuition to operate in combination with and transcend the evidence base that underpins clinical practice); and empathy (a reciprocal being alone together in which the patient becomes able to trust the therapist as understanding). |
|  | Willems 2005^155^ | Doctors  Various long-term conditions  N/A | To explore whether patients’ socio-economic status influences doctor–patient communication  Literature review | Summarises evidence from twelve studies that together show that doctors adopt more directive and less participatory consulting styles, and give less information, when working with people from lower social classes. Suggests patients from lower social classes are often disadvantaged because their doctors misperceive their desire and need for information and their ability to take part in the care process. |
|  | Williams 2011^156^ | GPs and practice-based asthma nurses  Asthma, primary care.  Scotland | To explore and compare the asthma goals of health professionals and people with asthma, and to identify barriers to achieving shared goals  Interviews | Distinguishes between ‘end state’ and ‘mediating goals’ and identifies differences between health professionals’ and patients’ views of these. Reports that while health professionals who focused on a medical model of asthma sometimes acknowledged asthma control as a mediating goal, their views of ‘normal life’ end state goals tended to emphasise freedom from physical activity constraint due to asthma. Patients, in contrast, tended to emphasise participation in family and social life. Notes that both patients and health professionals saw non-pharmacological asthma management issues as outside the medical domain and some professionals were reluctant to advise on lifestyle issues (e.g. pets) for fear of upsetting patients. Offers useful reflections on tensions between patients’ private lives and public selves in consultations, and on the constraints roles can place on the expression and acceptance of goals. |
|  | Wilson 2006^157^ | Nurses, doctors, physiotherapists  Various long-term conditions  England | To explore how patient expertise is viewed, interpreted, defined and experienced by patients and health professionals.  Focus groups, interviews and observation | Observes that nurses (other than nurse specialists) were generally more anxious about expert patients than other health professionals. Reports that nurses and other professionals relied heavily on physical measurements (e.g. of patients’ blood glucose levels) to judge patients’ expertise, and suggested patients often appeared to know more than they did. Notes that nurses were particularly concerned about the time implications of patients who sought and wanted to discuss information, and less likely than other health professionals to think that an early investment of time could reduce later time demands. Reports that nurses were the only professionals in this study to feel uncomfortable with and challenged by knowledgeable patients, and that nurses were particularly concerned that they would be held legally accountable for patients’ self-management behaviours. Suggests that nurses were more effective in responding to patients’ emotional and psychological needs, but notes that only nurse specialists articulated this as one of their skills, and that some thought “it doesn’t say much for nursing” if emotional support is all nurses are seen as good at. |
|  | Wright 2012^158^ | Doctors and nurses working in cancer care  Cancers  UK | To examine views about roles and responsibilities in the management of patients’ social difficulties  Interviews (clinicians and patients) | Groups social difficulties into three main categories (everyday living, money matters, self and others). Reports that most staff considered difficulties with everyday living within their remit and notes that doctors looked for clinical explanations and solutions, while nurses took a more holistic approach that included attention to social context. Reports that staff were less confident broaching difficulties with money matters or the psychosocial and family relationship issues considered under ‘self and others’. Notes that most patients also thought psychosocial issues were not for discussion in routine hospital consultations unless they related to ‘treatable’ symptoms such as pain. Outlines a hierarchy of interventions relating to social difficulties, ranging from general discussion, through encouraging mobilisation of commonly available resources, providing information, supportive intervention (clinical or advocacy), to referral for specialist help. |
|  | Yawn 2001^159^ | Family physicians  Diabetes, other chronic conditions, acute illnesses  USA | To compare the content of outpatient visits for different conditions  Observations of consultations | Reports differences in what physicians did in visits (consultations) for diabetes and visits for both other chronic diseases and acute illnesses. Compared with consultations for other chronic disease, consultations for diabetes included more time spent on nutrition counselling, health education and feedback on test results and less time on chatting (small talk, humour, etc. not related to the current visit, but perhaps useful to build rapports). Compared with visits for acute illness, visits for diabetes were longer and involved more dietary advice, assessment of compliance and negotiation. |
|  | Yen 2011^160^ | Doctors, nurses, allied health professionals, pharmacists  COPD, diabetes, heart failure  Australia | To investigate health professionals’ reactions to findings from a study of patients’ experiences of chronic illness  Focus groups and interviews | Reports that the health professionals generally agreed that people with chronic illness experienced problems with competing demands in self-management, financial pressures and co-morbidity, but that they interpreted these problems differently and had different ideas about potential solutions. Notes that health professionals interpreted patients’ problems mainly in terms of (a) compliance (apparently assuming that patients had full control over the management of their condition and sometimes viewing a lack of compliance as a matter of moral failure) and (b) service fragmentation. Comments that the health professionals did not factor information from what they were told about patients’ experiences into their views about systems improvement. Reports that health professionals thought solutions depended on additional resources being allocated to their own activities. |
|  | Yohannes 2012^161^ | GPs  COPD, primary care  England | To investigate GPs’ views and experiences in recognising and treating depression in patients with COPD  Questionnaires | Shows that GPs recognise they have limited scope to provide some of the kinds of psychological support that would be useful for self-management. Reports that 96% of GPs who responded to the questionnaires either agreed or strongly agreed that ‘depression impairs self-management of COPD’. 43% either agreed or strongly agreed that ‘It is frequently difficult to convince patients with COPD that their depression needs treatment’. GPs also commented on long waiting lists for psychological treatments for depression as a barrier to treatment*.* |
|  | Young 2012^162^ | Nurses (including learning disability specialists), GPs, psychiatrist  Cardiovascular disease in people with learning disabilities  Scotland | To investigate the perceptions of people with learning disabilities, carers and care staff, and health professionals on supported self-management of cardiovascular disease  Interviews (with vignettes) | Reports that health professionals, consistently with service users and carers, recognised the challenges of adopting health-promoting lifestyle changes, and considered it important to facilitate the involvement of people with learning disabilities in decision-making about how they manage their conditions. Health professionals recognised the importance of support from carers and care staff who know and work closely with people with learning disabilities. For their own part, they commented on support for self-management in people with learning disabilities in terms of involving people in decisions and outcomes, reinforcing decisions, encouraging people (rather than directing them), using accessible information, and taking extra time to explain. |
|  | Zakrisson 2010^163^ | Asthma/COPD nurses  COPD  Sweden | To describe nurses’ experiences of educating patients in primary health care  Interviews | Reports findings primarily in terms of relationships between nurses feeling supported (or not) and their feeling secure in the development and delivery of patient education. Mentions that nurses can feel important to the patient because of their ability to be available, to listen, and to co-ordinate care. Describes nurses’ efforts to meet patients’ worries and fears, relieve patients’ sense of guilt, support self-care by reflecting on patient education and adapting to individual patients; acknowledges that nurses could experience difficulties with these, including frustration when patients did not stop smoking. Notes the discouraging effects of negative attitudes from colleagues, both towards the patients and towards their own roles. |
|  | Zantar 2012^164^ {FRENCH) | Doctors  Diabetes  France | To consider how the management of fasting by Muslim diabetic patients who wish to participate in Ramadan can be assisted by doctors | Reports thinking based on efforts to define new approaches to fasting to minimise complications. Outlines various recommendations about fasting based on type of diabetes, case details, complications or recent episodes of hypoglycaemia, etc. Reflects an assumption that doctors’ roles might be to ‘authorise’ participation. Also notes that questions about fasting present a good opportunity for doctors to motivate patients to correct imbalances and to reinforce the risks of eventual complications. |

**REFERENCES**

1. Abbott S, Burns J, Gleadell A, Gunnell C. Community nurses and self-management of blood glucose*. Br J Community Nurs* 2007;**12:**6-11.

2. Abbott PA, Davison JE, Moore LF. Challenges and benefits of implementing a chronic disease self-management program in an aboriginal community controlled health setting*. Aust J Prim Health* 2007;**13:**35-9.

3. Adolfsson ET, Smide B, Gregeby E, Fernstrom L, Wikblad K. Implementing empowerment group education in diabetes*. Patient Educ Couns* 2004;**53:**319-24.

4. Albert NM. Promoting self-care in heart failure: state of clinical practice based on the perspectives of healthcare systems and providers.*. J Cardiovasc Nurs* 2008;**23:**277-84.

5. Aliotta SL, Grieve K, Giddens JF, Dunbar L, Groves C, Frey K, et al. Guided care: a new frontier for adults with chronic conditions*. Prof Case Manag* 2008;**13:**151-8.

6. Appiah B, Hong Y, Ory MG, Helduser JW, Begaye D, Bolin JN, et al. Challenges and opportunities for implementing diabetes self-management guidelines*. JABFM* 2013;**26:**90-2.

7. Asimakopoulou K, Newton P, Sinclair AJ, Scambler S. Health care professionals' understanding and day-to-day practice of patient empowerment in diabetes; time to pause for thought? *Diabetes Res Clin Pract* 2012;**95:**224-9.

8. Bancroft M. Promoting patient power*. Nurs Standard* 2008;**22:**22-3.

9. Bergsten U, Bergman S, Fridlund B, Arvidsson B. "Delivering knowledge and advice": Healthcare providers' experiences of their interaction with patients' management of rheumatoid arthritis*. Int J Qual Stud Health Well-being* 2011;**6**.(4).

10. Beverly EA, Ritholz MD, Brooks KM, Hultgren BA, Lee Y, Abrahamson MJ, et al. A qualitative study of perceived responsibility and self-blame in type 2 diabetes: reflections of physicians and patients*. J Gen Intern Med* 2012;**27:**1180-7.

11. Bhattacharyya OK, Rasooly IR, Naqshbandi M, Estey EA, Esler J, Toth E, et al. Challenges to the provision of diabetes care in first nations communities: results from a national survey of healthcare providers in Canada*. BMC Health Serv Res* 2011;**11:**283.

12. Bieber C, Muller KG, Blumenstiel K, Schneider A, Richter A, Wilke S, et al. Long-term effects of a shared decision-making intervention on physician-patient interaction and outcome in fibromyalgia. A qualitative and quantitative 1 year follow-up of a randomized controlled trial*. Patient Educ Couns* 2006;**63:**357-66.

13. Blakeman T, Macdonald W, Bower P, Gately C, Chew-Graham C. A qualitative study of GPs' attitudes to self-management of chronic disease*. Br J Gen Pract* 2006;**56:**407-14.

14. Blakeman T, Bower P, Reeves D, Chew-Graham C. Bringing self-management into clinical view: a qualitative study of long-term condition management in primary care consultations*. Chronic Ill* 2010;**6:**136-50.

15. Bower P, Macdonald W, Harkness E, Gask L, Kendrick T, Valderas JM, et al. Multimorbidity, service organization and clinical decision making in primary care: a qualitative study*. Fam Pract* 2011;**28:**579-87.

16. Brez S, Rowan M, Malcolm J, Izzi S, Maranger J, Liddy C, et al. Transition from specialist to primary diabetes care: a qualitative study of perspectives of primary care physicians*. BMC Fam Pract* 2009;**10:**39.

17. Brierley S, Eiser C, Johnson B, Young V, Heller S. Working with young adults with Type 1 diabetes: Views of a multidisciplinary care team and implications for service delivery*. Diab Med* 2012;**29:**677-81.

18. Brown A. Chronic leg ulceration in the community: changing the focus*. Br J Commun Nurs 2010 Suppl S6, S8, S10 passim*

19. Carbone ET, Rosal MC, Torres MI, Goins KV, Bermudez OI. Diabetes self-management: perspectives of Latino patients and their health care providers*. Patient Educ Couns* 2007;**66:**202-10.

20. Cardol M, Rijken M, van Schrojenstein Lantman-de V. Attitudes and dilemmas of caregivers supporting people with intellectual disabilities who have diabetes*. Patient Educ Couns* 2012;**87:**383-8.

21. Cass S, Ball L, Leveritt M. Australian practice nurses' perceptions of their role and competency to provide nutrition care to patients living with chronic disease*. Aust J Prim Health* 2014;**20:**203-8.

22. Catalano T, Kendall E, Vandenberg A, Hunter B. The experiences of leaders of self-management courses in Queensland: exploring Health Professional and Peer Leaders' perceptions of working together*. Health Soc Care Community* 2009;**17:**105-15.

23. Cavan DA. Structuring diabetes services to support self-management*. Pract Diab Int* 2010;**27:**164-5.

24. Chin MH, Cook S, Jin L, Drum ML, Harrison JF, Koppert J, et al. Barriers to providing diabetes care in community health centers*. Diab Care* 2001;**24:**268-74.

25. Ciccone MM, Aquilino A, Cortese F, Scicchitano P, Sassara M, Mola E, et al. Feasibility and effectiveness of a disease and care management model in the primary health care system for patients with heart failure and diabetes (Project Leonardo)*. Vasc Health Risk Manag* 2010;**6:**297-305.

26. Clark M, Hampson SE. Comparison of patients' and healthcare professionals' beliefs about and attitudes towards Type 2 diabetes*. Diab Med* 2003;**20:**152-4.

27. Clark NM, Stoll S, Youatt EJ, Sweetman M, Derry R, Gorelick A. Fostering epilepsy self management: The perspectives of professionals*. Epilepsy Behav* 2010;**19:**255-63.

28. Cleanthous S, Newman SP, Shipley M, Isenberg DA, Cano SJ. What constitutes uncertainty in systemic lupus erythematosus and rheumatoid arthritis? *Psychol Health* 2013;**28:**171-88.

29. Collins S, Drew P, Watt I, Entwistle V. 'Unilateral' and 'bilateral' practitioner approaches in decision-making about treatment*. Soc Sci Med* 2005;**61:**2611-27.

30. Coventry PA, Fisher L, Kenning C, Bee P, Bower P. Capacity, responsibility, and motivation: a critical qualitative evaluation of patient and practitioner views about barriers to self-management in people with multimorbidity*. BMC Health Serv Res* 2014;**14**:536.

31. Day M, McCarthy G, Leahy-Warren P. Professional Social Workers' Views on Self-Neglect: An Exploratory Study*. Br J Social Work* 2012;**42:**725-43.

32. Day JL, Coles C, Walford S. Self-management in diabetes: training implications for professional carers*. Clin Med* 2003;**3:**338-41.

33. Delea D, Shrader S, Phillips C. A week-long diabetes simulation for pharmacy students*. Am J Pharmaceut Educ* 2010;**74:**130.

34. Denford S, Frost J, Dieppe P, Britten N. Doctors' understanding of individualisation of drug treatments: A qualitative interview study*. BMJ Open* 2013;**3**:5.

35. Detaille SI, Haafkens JA, Hoekstra JB, van Dijk FJ. What employees with diabetes mellitus need to cope at work: views of employees and health professionals*. Patient Educ Couns* 2006;**64:**183-90.

36. Dures E, Hewlett S, Ambler N, Jenkins R, Clarke J, Gooberman-Hill R. Rheumatology clinicians' experiences of brief training and implementation of skills to support patient self-management*. BMC Musculoskel Disord* 2014;**15:**108.

37. Eldh AC, Ehnfors M, Ekman I. The meaning of patient participation for patients and nurses at a nurse-led clinic for chronic heart failure*. Eur J Cardiovasc Nurs* 2006;**5:**45-53.

38. Elliott N. 'Mutual intacting': a grounded theory study of clinical judgement practice issues*. J Advanc Nurs* 2010;**66:**2711-21.

39. Entwistle VA, Cribb A. Enabling people to live well: fresh thinking about collaborative approaches to care for people with long term conditions. London: The Health Foundation, 2013.

40. Ferrante JM, Piasecki AK, Ohman-Strickland PA, Crabtree BF. Family physicians' practices and attitudes regarding care of extremely obese patients*. Obesity* 2009;**17:**1710-6.

41. Ford S, Schofield T, Hope T. Observing decision-making in the general practice consultation: who makes which decisions? *Health Expect* 2006;**9:**130-7.

42. Fox A. Intensive diabetes management: negotiating evidence-based practice*. Can J Diet Pract Res* 2010;**71:**62-8.

43. Furler J, Spitzer O, Young D, Best J. Insulin in general practice - barriers and enablers for timely initiation*. Aust Fam Physician* 2011;**40:**617-21.

44. Gambling T, Long AF. The realisation of patient-centred care during a 3-year proactive telephone counselling self-care intervention for diabetes*. Patient Educ Couns* 2010;**80:**219-26.

45. Garrett DG, Martin LA. The asheville project: participants' perceptions of factors contributing to the success of a patient self-management diabetes program*. JAPhA* 2003;**43:**185-90.

46. Gillibrand W, Taylor J, Hughes JG. Practice nurses' views of their diabetes care*. Pract Nurs* 2004;**15:**144-9.

47. Goodrich DE, Buis LR, Janney AW, Ditty MD, Krause CW, Zheng K, et al. Integrating an internet-mediated walking program into family medicine clinical practice: a pilot feasibility study*. BMC Med Inf DecisMak* 2011;**11:**47.

48. Graham AS, Hammond A, Williams AE. Foot health education for people with rheumatoid arthritis: the practitioner's perspective*. J Foot Ankle Res* 2012;**5:**2.

49 Granger BB, Sandelowski M, Tahshjain H, Swedberg K, Ekman I. A qualitative descriptive study of the work of adherence to a chronic heart failure regimen: patient and physician perspectives*. J Cardiovasc Nurs* 2009;**24:**308-15.

50. Greiner KA. Patient-provider relations--understanding the social and cultural circumstances of difficult patients*. Bioethics Forum* 2000;**16:**7-12.

51. Grimaldi A. How to help the patient motivate himself? *Diab Metabol* 2012;**38:**S59-S64.

52. Grimmer-Somers K, Guerin M, Luker J, Jones D, Zucco M. Guiding principles for chronic disease management for vulnerable and disadvantaged people: pilot study findings*. Internet J Allied Health Sci Pract* 2009;**7:**8p.

53. Hajos TRS, Polonsky WH, Twisk JWR, Dain MP, Snoek FJ. Do physicians understand Type 2 diabetes patients' perceptions of seriousness; the emotional impact and needs for care improvement? A cross-national survey*. Patient Educ Counsel* 2011;**85:**258-63..

54. Hale LA, Piggot J. Exploring the content of physiotherapeutic home-based stroke rehabilitation in New Zealand*. Arch Phys Med Rehabil* 2005;**86:**1933-40.

55. Harrison MB, Graham ID, Logan J, Toman C, Friederg E. Evidence to practice: pre-post-implementation study of a patient/provider resource for self-management with heart failure*. Int J Evid Based Healthc* 2007;**5:**92-101.

56. Heldal F, Steinsbekk A. Norwegian healthcare professionals' perceptions of patient knowledge and involvement as basis for decision making in hematology*. Oncol Nurs Forum* 2009;**36:**E93-E8.

57. Holley UA. Social isolation: a practical guide for nurses assisting clients with chronic illness*. Rehabil Nurs* 2007;**32:**51-6.

58. Holmstrom I, Larsson J, Lindberg E, Rosenqvist U. Improving the diabetes-patient encounter by reflective tutoring for staff*. Patient Educ Couns* 2004;**53:**325-32.

59. Hopp FP, Hogan MM, Woodbridge PA, Lowery JC. The use of telehealth for diabetes management: a qualitative study of telehealth provider perceptions*. Implement Sci* 2007;**2:**14.

60. Hörnsten Å, Lundman B, Almberg A, Sandström H. Nurses' experiences of conflicting encounters in diabetes care*. Eur Diab Nurs* 2008;**5:**64-9.

61. Horsburgh MP, Bycroft JJ, Goodyear-Smith FA, Roy DE, Mahony FM, Donnell EC, et al. The Flinders ProgramTM of chronic condition self-management in New Zealand: survey findings*. J Prim Health Care* 2010;**2:**288-93.

62. Hughes JL. Chronic Fatigue Syndrome and Occupational Disruption in Primary Care: is there a Role for Occupational Therapy? *Br J Occup Therap* 2009;**72:**2-10.

63. Hunt LM, Arar NH. An analytical framework for contrasting patient and provider views of the process of chronic disease management*. Med Anthropol Q*  2001;**15:**347-67.

64. Hussain T, Allen A, Halbert J, Anderson CAM, Boonyasai RT, Cooper LA. Provider Perspectives on Essential Functions for Care Management in the Collaborative Treatment of Hypertension: The P.A.R.T.N.E.R. Framework*. J Gen Intern Med* 2015;**30:**454-61.

65. Jallinoja P, Absetz P, Kuronen R, Nissinen A, Talja M, Uutela A, et al. The dilemma of patient responsibility for lifestyle change: perceptions among primary care physicians and nurses*. Scand J Prim Health Care* 2007;**25:**244-9.

66. Jeffery V, Ervin K. Early intervention in chronic disease--four years on: barriers to implementing self-management strategies*. J Allied Health* 2014;**43:**e1-e3.

67. Jeffrey JE, Foster NE. A qualitative investigation of physical therapists' experiences and feelings of managing patients with nonspecific low back pain*. Phys Therap* 2012;**92:**266-78.

68. Johnson M, Newton P, Jiwa M, Goyder E. Meeting the Educational Needs of People at Risk of Diabetes-Related Amputation: A Vignette Study with Patients and Professionals*. Health Expect* 2005;**8:**324-33.

69. Johnson M, Newton P, Goyder E. Patient and professional perspectives on prescribed therapeutic footwear for people with diabetes: a vignette study*. Patient Educ Couns* 2006;**64:**167-72.

70. Johnson K, Valdez RS, Casper GR, Kossman SP, Carayon P, Or CK, et al. Experiences of technology integration in home care nursing*. AMIA Ann Symp Proceed* 2008*:389-93*

71. Johnston SE, Liddy CE, Ives SM. Self-management support: a new approach still anchored in an old model of health care*. Can J Public Health* 2011;**102:**68-72.

72. Jones A, Pill R, Adams S. Qualitative study of views of health professionals and patients on guided self management plans for asthma*. BMJ* 2000;**321:**1507-10.

73. Jones MI, Greenfield SM, Bray EP, Hobbs FR, Holder R, Little P, et al. Patient self-monitoring of blood pressure and self-titration of medication in primary care: the TASMINH2 trial qualitative study of health professionals' experiences*. Br J Gen Pract* 2013;**63:**e378-e85.

74. Jowsey T, Jeon YH, Dugdale P, Glasgow NJ, Kljakovic M, Usherwood T. Challenges for co-morbid chronic illness care and policy in Australia: a qualitative study*. Aust NZ Health Policy* 2009;**6:**22.

75. Junius-Walker U, Voigt I, Wrede J, Hummers-Pradier E, Lazic D, Dierks ML. Health and treatment priorities in patients with multimorbidity: report on a workshop from the European General Practice Network meeting 'Research on multimorbidity in general practice'*. Eur J Gen Pract* 2010;**16:**51-4.

76. Jutterstrom L, Graneheim U, Isaksson U, H+ƒ-¶rnsten ƒ. Ideal versus Real Conditions for Type 2 Diabetes Care: Diabetes Specialty Nurses' Perspectives*. Internet J Adv Nurs Pract* 2012;**11:**1-.

77. Kendall E, Rogers A. Extinguishing the social?: state sponsored self-care policy and the Chronic Disease Self-management Programme*. Disabil Soc* 2007;**22:**129-43.

78. Kennedy AP, Rogers AE. Improving patient involvement in chronic disease management: the views of patients, GPs and specialists on a guidebook for ulcerative colitis*. Patient Educ Counsel* 2002;**47:**257-63.

79. Kennedy A, Gask L, Rogers A. Training Professionals to Engage with and Promote Self-Management*. Health Educ Res* 2005;**20:**567-78.

80.Kennedy A, Rogers A, Bower P. Support for Self Care for Patients with Chronic Disease*. BMJ* 2007;**335:**968-70.

81. Kent D, Haas L, Randal D, Lin E, Thorpe CT, Boren SA, et al. Healthy coping: issues and implications in diabetes education and care*. Pop Health Manag* 2010;**13:**227-33.

82. Kirby SE, Dennis SM, Bazeley P, Harris MF. What distinguishes clinicians who better support patients for chronic disease self-management? *Aust J Primary Health* 2012;**18:**220-7.

83.Kosmala-Anderson JP, Wallace LM, Turner A. Confidence matters: a Self-Determination Theory study of factors determining engagement in self-management support practices of UK clinicians*. Psychol Health Med* 2010;**15:**478-91.

84. Kremer H, Bader A, O'Cleirigh C, Bierhoff HW, Brockmeyer NH. The decision to forgo antiretroviral therapy in people living with HIV compliance as paternalism or partnership? *Eur J Med Res* 2004;**9:**61-70.

85. Lake AJ, Staiger PK. Seeking the views of health professionals on translating chronic disease self-management models into practice*. Patient Educ Couns* 2010;**79:**62-8.

86. Langer S, Chew-Graham CA, Drinkwater J, Afzal C, Keane K, Hunter C, et al. A motivational intervention for patients with COPD in primary care: qualitative evaluation of a new practitioner role*. BMC Fam Pract* 2014;**15**.

87. Langstrup H. Making connections through online asthma monitoring*. Chronic Ill* 2008;**4:**118-26.

88. Lemay CA, Ferguson WJ, Hargraves JL. Community health worker encounter forms: a tool to guide and document patient visits and worker performance*. Am J Public Health* 2012;**102:**e70-5.

89. Löfman P, Pelkonen M, Pietilä A. Evaluation of self-determination for patients with rheumatoid arthritis: starting points for the development of nursing practice [Finnish]*. Hoitotiede* 2003;**15:**264-76.

90. Lundh L, Rosenhall L, Tornkvist L. Care of patients with chronic obstructive pulmonary disease in primary health care*. J Adv Nurs* 2006;**56:**237-46.

91. Macdonald W, Rogers A, Blakeman T, Bower P. Practice nurses and the facilitation of self-management in primary care*. J Adv Nurs* 2008;**62:**191-9.

92. Macneela P, Gibbons A, McGuire B, Murphy A. "We need to get you focused": general practitioners' representations of chronic low back pain patients*. Qual Health Res* 2010;**20:**977-86.

93. Malone A, Davies M, Dempster M. Providing psychological services for people with diabetes*. Pract Diab Int* 2005;**22:**244-8.

94. McCann TV, Clark E. Advancing self-determination with young adults who have schizophrenia*. J Psychiat Mental Health Nurs* 2004;**11:**12-20.

95. McDonald R, Rogers A, Macdonald W. Dependence and identity: Nurses and chronic conditions in a primary care setting*. J Health, Organ Manag* 2008;**22:**294-308.

96. McIntosh A, Shaw CF. Barriers to patient information provision in primary care: patients' and general practitioners' experiences and expectations of information for low back pain*. Health Expect* 2003;**6:**19-29.

97. McLane L, Jones K, Lydiatt W, Lydiatt D, Richards A. Taking away the fear: a grounded theory study of cooperative care in the treatment of head and neck cancer*. Psycho-Oncol* 2003;**12:**474-90.

98. Mirzaei M, Aspin C, Essue B, Jeon YH, Dugdale P, Usherwood T, et al. A patient-centred approach to health service delivery: improving health outcomes for people with chronic illness*. BMC Health Serv Res* 2013;**13:**251.

99. Moffat M, Cleland J, van der Molen T, Price D. Poor communication may impair optimal asthma care: a qualitative study*. Fam Pract* 2007;**24:**65-70.

100. Mulder BC, van B, Lokhorst AM, van W. Quality assessment of practice nurse communication with type 2 diabetes patients*. Patient Educ Counsel*  2015;**98:**156-61.

101. Nam S, Chesla C, Stotts NA, Kroon L, Janson SL. Barriers to diabetes management: patient and provider factors. *Diab Res Clin Pract* 2011;**93:**1-9.

102. Nasmith L, Cote B, Cox J, Inkell D, Rubenstein H, Jimenez V, et al. The challenge of promoting integration: Conceptualization, implementation, and assessment of a pilot care delivery model for patients with type 2 diabetes*. Fam Med* 2004;**36:**40-5.

103. Nelson PA, Barker Z, Griffiths CE, Cordingley L, Chew-Graham CA, Team I. 'On the surface': a qualitative study of GPs' and patients' perspectives on psoriasis*. BMC Fam Pract* 2013;**14:**158.

104. Newton P, Sasha S, Koula A. Marrying contradictions: healthcare professionals perceptions of empowerment in the care of people with Type 2 Diabetes*. Patient Educ Counsel* 2011;**85:**e326-e9.

105. Norris M, Kilbride C. From dictatorship to a reluctant democracy: stroke therapists talking about self-management*. Disabil Rehabil* 2014;**36:**32-8.

106. Paterson B. Myth of empowerment in chronic illness*. J Adv Nurs* 2001;**34:**574-81.

107. Peyrot M, Rubin RR. Physician perception and recommendation of insulin pens for patients with type 2 diabetes mellitus*. Curr Med Res Opin* 2008;**24:**2413-22.

108. Peyrot M, Rubin RR. Access to diabetes self-management education*. Diab Educ* 2008;**34:**90-7.

109. Peytremann-Bridevaux I, Lauvergeon S, Mettler D, Burnand B. Diabetes care: Opinions, needs and proposed solutions of Swiss patients and healthcare professionals: a qualitative study*. Diab Res Clin Pract* 2012;**97:**242-50.

110. Phillips K, Wood F, Kinnersley P. Tackling obesity: the challenge of obesity management for practice nurses in primary care*. Fam Pract* 2014;**31:**51-9.

111. Pitt VJ, O'Connor D, Green S. Referral of people with osteoarthritis to self-management programmes: barriers and enablers identified by general practitioners*. Disabil Rehabil* 2008;**30:**1938-46.

112. Pooley CG, Gerrard C, Hollis S, Morton S, Astbury J. 'Oh it's a wonderful practice... you can talk to them': a qualitative study of patients' and health professionals' views on the management of type 2 diabetes*. Health Soc Care Community* 2001;**9:**318-26.

113. Proot IM, Abu-Saad HH, Van Oorsouw GG, Stevens JJ. Autonomy in stroke rehabilitation: the perceptions of care providers in nursing homes*. Nurs Ethics* 2002;**9:**36-50.

114. Ratanawongsa N, Bhandari VK, Handley M, Rundall T, Hammer H, Schillinger D. Primary care provider perceptions of the effectiveness of two self-management support programs for vulnerable patients with diabetes*. J Diab Sci Technol* 2012;**6:**116-24.

115. Ratanawongsa N, Crosson JC, Schillinger D, Karter AJ, Saha CK, Marrero DG. Getting under the skin of clinical inertia in insulin initiation: the Translating Research Into Action for Diabetes (TRIAD) Insulin Starts Project*. Diab Educ* 2012;**38:**94-100.

116. Richards DA, Lankshear AJ, Fletcher J, Rogers A, Barkham M, Bower P, et al. Developing a U.K. protocol for collaborative care: a qualitative study*. Gen Hosp Psychiatry* 2006;**28:**296-305.

117. Ring N, Jepson R, Hoskins G, Wilson C, Pinnock H, Sheikh A, et al. Understanding what helps or hinders asthma action plan use: a systematic review and synthesis of the qualitative literature*. Patient Educ Couns* 2011;**85:**e131-e43.

118. Ritholz MD, Beverly EA, Brooks KM, Abrahamson MJ, Weinger K. Barriers and facilitators to self-care communication during medical appointments in the United States for adults with type 2 diabetes*. Chronic Ill* 2014;**10:**303-13.

119. Thille P, Ward N, Russell G. Self-management support in primary care: enactments, disruptions, and conversational consequences*. Soc Sci Med* 2014;**108:**97-105.

120. Robinson A, Courtney-Pratt H, Lea E, Cameron-Tucker H, Turner P, Cummings E, et al. Transforming clinical practice amongst community nurses: mentoring for COPD patient self-management*. J Clin Nurs* 2008;**17:**370-9.

121. Rogers WA. Whose autonomy? Which choice? A study of GPs' attitudes towards patient autonomy in the management of low back pain*. Fam Pract* 2002;**19:**140-5.

122. Rogers A, Kennedy A, Nelson E, Robinson A. Uncovering the limits of patient-centeredness: Implementing a self-management trial for chronic illness*. Qual Health Res* 2005;**15:**224-39.

123. Rohde A, Townley-O'Neill K, Trendall K, Worrall L, Cornwell P. A comparison of client and therapist goals for people with aphasia: A qualitative exploratory study*. Aphasiology* 2012;**26:**1298-315.

124 Ross J, Stefan H, Schauble B, Day R, Sander JW. European survey of the level of satisfaction of patients and physicians in the management of epilepsy in general practice*. Epilepsy Beh* 2010;**19:**36-42.

125. Roy D, Mahony F, Horsburgh M, Bycroft J. Partnering in primary care in New Zealand: clients' and nurses' experience of the Flinders Program in the management of long-term conditions*. J Nurs Healthc Chronic Ill* 2011;**3:**140-9.

126. Rutter P, Tsang G. Nurse independent prescribers' views on recent medicine switches*. Nurs Prescrib* 2011;**9:**195-9.

127. Rychetnik L, McCaffery K, Morton RL, Thompson JF, Menzies SW, Irwig L. Follow-up of early stage melanoma: specialist clinician perspectives on the functions of follow-up and implications for extending follow-up intervals*. J Surg Oncol* 2013;**107:**463-8.

128. Scambler S, Newton P, Sinclair AJ, Asimakopoulou K. Barriers and opportunities of empowerment as applied in diabetes settings: a focus on health care professionals' experiences*. Diab Res Clin Pract* 2012;**97:**e18-e22.

129. Schaevers V, Schoonis A, Frickx G, Verleden G, Jans C, Rosseel C, et al. Implementing a standardized, evidence-based education program using the patient's electronic file for lung transplant recipients*. Prog Transplant* 2012;**22:**264-70.

130. Schold AK, Ylikivela R, Lindstrom K, Ostgren CJ, Grodzinsky E. The options of the management of self-monitoring of blood glucose in primary health care centres by the diabetes nurses and patients*. Primary Care Diab* 2013;**7:**July.

131. Seto E, Leonard KJ, Masino C, Cafazzo JA, Barnsley J, Ross HJ. Attitudes of heart failure patients and health care providers towards mobile phone-based remote monitoring*. J Med Internet Res* 2010;**12:**e55.

132. Seto E, Leonard KJ, Cafazzo JA, Barnsley J, Masino C, Ross HJ. Perceptions and experiences of heart failure patients and clinicians on the use of mobile phone-based telemonitoring*. J Med Internet Res* 2012;**14:**e25.

133. Sharma S, Wallace LM, Kosmala-Anderson J, Turner A. A process evaluation using a Self Determination Theory measure of the co-delivery of self management training by clinicians and by lay tutors*. Patient Educ Couns* 2013;**90:**38-45.

134. Sheares BJ, Du Y, Vazquez TL, Mellins RB, Evans D. Use of written treatment plans for asthma by specialist physicians*. Ped Pulmonol* 2007;**42:**348-56.

135. Shortus T, Kemp L, McKenzie S, Harris M. 'Managing patient involvement': provider perspectives on diabetes decision-making*. Health Expect* 2013;**16:**189-98.

136. Simm R, Hastie L, Weymouth E. Is training in solution-focused working useful to community matrons? *Br J Community Nurs* 2011;**16:**598-603.

137. Singleton JK. Nurses' perspectives of encouraging clients' care-of-self in a short-term rehabilitation unit within a long-term care facility*. Rehabil Nurs J* 2000;**25:**23-30.

138. South J, Darby F, Bagnall A, White A. Implementing a community-based self care training initiative: a process evaluation*. Health Soc Care Community* 2010;**18:**662-70.

139. Steinmann WC, Chitima-Matsiga R, Bagree S. What are specialist and primary care clinicians' attitudes and practices regarding home blood pressure monitoring for hypertensive patients? *Missouri Med* 2011;**108:**443-7.

140. Steurer-Stey C, Fletcher M, Vetter W, Steurer J. Patient education in asthma: a survey of physicians' knowledge of the principles and implementation of self management in practice*. Swiss Med Week* 2006;**136:**561-5.

141. Sunaert P, Vandekerckhove M, Bastiaens H, Feyen L, Bussche PV, De MJ, et al. Why do GPs hesitate to refer diabetes patients to a self-management education program: a qualitative study*. BMC Fam Pract* 2011;**12:**94.

142. Thille PH, Russell GM. Giving patients responsibility or fostering mutual response-ability: family physicians' constructions of effective chronic illness management*. Qual Health Res* 2010;**20:**1343-52.

143. Thomson D. An ethnographic study of physiotherapists' perceptions of their interactions with patients on a chronic pain unit*. Physio Theory Pract* 2008;**24:**408-22.

144. Tisler A, Dunai A, Keszei A, Fekete B, Othmane TH, Torzsa P, et al. Primary-care physicians' views about the use of home/self blood pressure monitoring: nationwide survey in Hungary*. J Hypertension* 2006;**24:**1729-35.

145. Upshur CC, Luckmann RS, Savageau JA. Primary care provider concerns about management of chronic pain in community clinic populations*. J Gen Intern Med* 2006;**21:**652-5.

146. Urowitz S, Wiljer D, Dupak K, Kuehner Z, Leonard K, Lovrics E, et al. Improving diabetes management with a patient portal: a qualitative study of diabetes self-management portal*. J Med Internet Res* 2012;**14:**e158.

147.van den Berg NJ, of ten Have WH, Nagelkerke AF, Bindels PJ, van der Palen J, van Aalderen WM. What general practitioners and paediatricians think about their patients' asthma*. Patient Educ Couns*  2005;**59:**182-5.

148. van Hooft SM, Dwarswaard J, Jedeloo S, Bal R, van Staa A. Four perspectives on self-management support by nurses for people with chronic conditions: A Q-methodological study*. Int J Nurs Studies* 2015;**52:**157-66.

149. Verlinde E, De LN, De MS, Deveugele M, Willems S. The social gradient in doctor-patient communication*. Int J Equity Health* 2012;**11:**12.

150. Vetter-Smith M, Lemaster J, Olsberg J, Kruse R, Day T, Mehr D. Providing diabetes self-management support in rural primary care clinics with nurse partners*. Western J Nurs Res* 2012;**34:**1023-42.

151. Visse MA, Teunissen T, Peters A, Widdershoven GA, Abma TA. Dialogue for air, air for dialogue: towards shared responsibilities in COPD practice*. Health Care Anal* 2010;**18:**358-73.

152. Warren CS, Schafer KJ, Crowley MEJ, Olivardia R. Treatment Providers With a Personal History of Eating Pathology: A Qualitative Examination of Common Experiences*. Eating Disord* 2013;**21:**295-309.

153. Watts SA, Gee J, O'Day ME, Schaub K, Lawrence R, Aron D, et al. Nurse practitioner-led multidisciplinary teams to improve chronic illness care: The unique strengths of nurse practitioners applied to shared medical appointments/group visits*. J Am Acad Nurse Pract* 2009;**21:**167-72.

154.Wilkin P. In search of the true self: a clinical journey through the vale of soul-making*. J Psychiatric Mental Health Nurs* 2006;**13:**12-8.

155. Willems S, De MS, Deveugele M, Derese A, De MJ. Socio-economic status of the patient and doctor-patient communication: does it make a difference?*. Patient Educ Couns* 2005;**56:**139-46.

156. Williams AM, Dennis S, Harris MF. How Effective Are the Linkages Between Self-management Programmes and Primary Care Providers, Especially for Disadvantaged Patients? *Chronic Ill* 2011;**7:**20-30.

157. Wilson PM, Kendall S, Brooks F. Nurses' responses to expert patients: the rhetoric and reality of self-management in long-term conditions: a grounded theory study*. Int J Nurs Studies* 2006;**43:**803-18.

158. Wright P, Bingham L, Taylor S, Hanif N, Podmore E, Velikova G. Managing social difficulties: roles and responsibilities of patients and staff*. Psycho-Oncol* 2012;**21:**20-8.

159. Yawn B, Zyzanski SJ, Goodwin MA, Gotler RS, Stange KC. Is diabetes treated as an acute or chronic illness in community family practice? *Diab Care* 2001;**24:**1390-6.

160. Yen L, Gillespie J, Rn YH, Kljakovic M, Anne BJ, Jan S, et al. Health professionals, patients and chronic illness policy: a qualitative study*. Health Expect* 2011;**14:**10-20.

161. Yohannes AM. General practitioners views and experiences in managing depression in patients with chronic obstructive pulmonary disease*. Expert Rev Respir Med* 2012;**6:**589-95.

162. Young AF, Naji S, Kroll T. Support for self-management of cardiovascular disease by people with learning disabilities*. Family Pract* 2012;**29:**467-75.

163. Zakrisson AB, Hagglund D. The asthma/COPD nurses' experience of educating patients with chronic obstructive pulmonary disease in primary health care*. Scand J Caring Sci* 2010;**24:**147-55.

164. Zantar A, Azzoug S, Belhimer F, Chentli F. Diabetes and Ramadan. [French]*. Presse Medicale* 2012;**41**:1084-8.
